# Downregulation of miR-130b~301b cluster is mediated by aberrant promoter methylation and impairs cellular senescence in prostate cancer

**DOI:** 10.1186/s13045-017-0415-1

**Published:** 2017-02-06

**Authors:** João Ramalho-Carvalho, Inês Graça, Antonio Gomez, Jorge Oliveira, Rui Henrique, Manel Esteller, Carmen Jerónimo

**Affiliations:** 1 Cancer Biology & Epigenetics Group—Research Center (CI-IPOP), Portuguese Oncology Institute of Porto (IPO Porto), Rua Dr António Bernardino de Almeida, 4200-072 Porto, Portugal; 2 Cancer Epigenetics and Biology Program, Bellvitge Biomedical Research Institute, Barcelona, Catalonia Spain; 3 Biomedical Sciences Graduate Program, Institute of Biomedical Sciences Abel Salazar, University of Porto (ICBAS-UP), Porto, Portugal; 4 School of Allied Health Sciences (ESTSP), Polytechnic of Porto, Porto, Portugal; 5 Department of Urology, Portuguese Oncology Institute of Porto (IPO Porto), Porto, Portugal; 6 Department of Pathology, Portuguese Oncology Institute of Porto (IPO Porto), Porto, Portugal; 7 Department of Pathology and Molecular Immunology, Institute of Biomedical Sciences Abel Salazar, University of Porto (ICBAS-UP), Porto, Portugal; 8 Institucio Catalana de Recerca i Estudis Avançats (ICREA), Barcelona, Catalonia Spain; 9 Department of Physiological Sciences II, School of Medicine, University of Barcelona, Barcelona, Catalonia Spain; 10 Currently at the Gene Regulation, Stem Cells and Cancer Programme, Centre for Genomic Regulation (CRG), The Barcelona Institute of Science and Technology, Barcelona, Spain

**Keywords:** miR-130b, miR-301b, microRNA, Senescence, Senescence-associated secretory phenotype, Prostate cancer

## Abstract

**Background:**

Numerous DNA-damaging cellular stresses, including oncogene activation and DNA-damage response (DDR), may lead to cellular senescence. Previous observations linked microRNA deregulation with altered senescent patterns, prompting us to investigate whether epigenetic repression of microRNAs expression might disrupt senescence in prostate cancer (PCa) cells.

**Methods:**

Differential methylation mapping in prostate tissues was carried using Infinium HumanMethylation450 BeadChip. After validation of methylation and expression analyses in a larger series of prostate tissues, the functional role of the cluster miR-130b~301b was explored using in vitro studies testing cell viability, apoptosis, invasion and DNA damage in prostate cancer cell lines. Western blot and RT-qPCR were performed to support those observations.

**Results:**

We found that the miR-130b~301b cluster directs epigenetic activation of cell cycle inhibitors required for DDR activation, thus stimulating the senescence-associated secretory phenotype (SASP). Furthermore, overexpression of miR-130b~301b cluster markedly reduced the malignant phenotype of PCa cells.

**Conclusions:**

Altogether, these data demonstrate that miR-130b~301b cluster overexpression might effectively induce PCa cell growth arrest through epigenetic regulation of proliferation-blocking genes and activation of cellular senescence.

**Electronic supplementary material:**

The online version of this article (doi:10.1186/s13045-017-0415-1) contains supplementary material, which is available to authorized users.

## Background

MicroRNAs (miRNAs) are small, non-coding RNAs that act as sequence-specific guides for Argonaute (AGO) proteins, which mediate post-transcriptional silencing of target mRNA [1]. miRNAs are transcribed from individual genes containing their own promoters or are originated intragenically from spliced segments of other genes [2]. They contain upstream regulatory elements and promoter regions, indicating that miRNAs might endure CpG promoter methylation via DNA methyltransferase (DMNT), histone modifications, as well as other regulatory alterations [1, 3]. Importantly, whereas miRNA genes transcription-start sites (TSS) are occasionally 5–10 kb away from the pre-miRNA sequence [4], promoter regions may be up to 50 kb apart, which may preclude the elucidation of transcriptional regulation of particular miRNAs [1]. Functional miRNAs result from sequential processing of pri-miRNAs by RNase III family enzymes DROSHA (nucleus) and DICER (cytoplasm). Unlike their protein-coding counterparts, however, miRNAs function as guides for identifying target mRNAs for repression [5].

MiRNAs are involved in development, homeostasis, cell cycle, apoptosis and in diverse pathological condition in nearly all vertebrate tissues [6]. Importantly, aberrant miRNA expression levels have been associated with promotion or arrest of tumorigenesis, through its ability to control the expression of a myriad of protein-coding and non-coding genes [7]. Concordantly, deregulation of miRNA expression has been reported in several malignancies, including prostate cancer (PCa) [3]. PCa is currently the most common non-cutaneous malignancy in developed countries and the second leading cause of death from cancer in men in the USA and in Europe, accounting for one in nine of all newly diagnosed cancers in men [8]. Nonetheless, altered miRNA expression patterns in PCa have been significantly understudied compared to other cancers, despite evidence suggesting a global downregulation of miRNA expression in both tumorigenesis and treatment resistance [9, 10].

Here, we examined how epigenetic alterations might contribute to miRNAs deregulation in PCa, focusing on the role of miR-130b~301b cluster. We found that miR-130b~301b cluster displays tumour-suppressive functions in vitro, influencing cell cycle, cell viability, apoptosis and invasion. Interestingly, an unprecedented effect of miR-130b~301b cluster on cellular senescence, which prevents cancer cell proliferation, was disclosed, suggesting that impairment of cellular senescence might underlie the deleterious effects of miR-130b~301b cluster downregulation in prostate carcinogenesis.

## Methods

### Patients and sample collection

Primary tumour tissues from 111 patients harbouring clinically localized PCa were prospectively collected, after diagnosis and primary treatment with radical prostatectomy at Portuguese Oncology Institute of Porto, Porto, Portugal (Additional file 1: Table S1). A set of 14 morphologically normal prostate tissues (MNPT) was procured from prostatic peripheral zone of bladder cancer patients submitted to cystoprostatectomy and which did not harbour concomitant PCa. All tissue specimens were promptly frozen after surgery. Upon histological confirmation of tumour or normal prostate tissue, fresh-frozen tissue fragments were trimmed to enhance yield of target cells (>70%). Histological slides from formalin-fixed paraffin-embedded tissue fragments were also routinely obtained from the surgical specimens and assessed for Gleason score and TNM stage. Relevant clinical data was collected from clinical charts and informed consent was obtained from all participants, according to institutional regulations. This study was approved by the institutional review board (Comissão de Ética para a Saúde) of Portuguese Oncology Institute of Porto, Portugal (CES-IPOPFG-EPE 205/2013).

### Nuclei acid extractions, bisulfite conversion and cDNA synthesis

DNA from fresh frozen tissue samples and cell lines was extracted using phenol:chloroform (Sigma). RNA was obtained using TRIzol (Invitrogen, Carlsbad, CA, USA) according to manufacturer’s instructions.

Bisulfite conversion of 1000 ng of genomic DNA was accomplished using EZ DNA Methylation Kit (Zymo Research), following manufacturer’s instructions.

Specific-miRNA cDNA was obtained using TaqMan MicroRNA Reverse Transcription Kit from Applied Biosystems (Foster City, CA, USA). Total cDNA synthesis was performed using high-capacity cDNA Reverse Transcription Kit (Applied Biosystems).

### Infinium HumanMethylation450 BeadChip

All DNA samples were assessed for integrity, quantity and purity by electrophoresis in a 1.3% agarose gel, picogreen quantification and nanodrop measurements. All samples were randomly distributed into 96-well plates. Bisulfite-converted DNA (200 ng) were used for hybridization on the HumanMethylation450 BeadChip (Illumina), comprising 25 PCa and 5 MNPT.

HumanMethylation450 BeadChip data were processed using Bioconductor minfi package [11]. The “Ilumina” procedure, which mimics the method of GenomeStudio (Illumina), was performed comprising background correction and normalization taking the first array of the plate as reference. Probes with one or more single-nucleotide polymorphisms (SNPs) with a minor allele frequency (MAF) >1% (1000 Genomes) in the first 10 bp of the interrogated CpG were removed. The methylation level (*β*) for each of the 485,577 CpG sites was calculated as the ratio of methylated signal divided by the sum of methylated and unmethylated signals, multiplied by 100. After normalization step, probes related to X and Y chromosomes were removed. All analyses were performed in human genome version 19 (hg19), and data was deposited in GEO repository under accession number GSE52955.

### Pyrosequencing

Specific sets of primers for PCR amplification and sequencing were designed using a specific software pack (PyroMark assay design version 2.0.01.15). Primer sequences were designed to hybridize, whenever possible, with CpG-free sites, ensuring methylation-independent amplification. PCR was performed under standard conditions with biotinylated primers, and the PyroMark Vacuum Prep Tool (Biotage, Uppsala, Sweden) was used to prepare single-stranded PCR products according to manufacturer’s instructions. Pyrosequencing reactions and methylation quantification were performed in a PyroMark Q96 System version 2.0.6 (QIAGEN) using appropriate reagents and recommended protocols.

### RT-qPCR

MiRNA transcript levels were assessed using TaqMan MicroRNA Assays specific for each miRNA (miR-130b, assay ID: 000456; miR-301b, assay ID: 002392) and normalized with RNU48 (assay ID: 001006; Applied Biosystems).

Real-time quantitative PCR (RT-qPCR) analysis was performed using gene-specific primers (Additional file 1: Table S2) and normalized to the expression of *GUSB* housekeeping gene.

### PCa cell lines

LNCaP cells were grown in RPMI 1640, DU145 cells were maintained in MEM and PC-3 cells were grown in 50% RPMI-50% F-12 medium (GIBCO, Invitrogen, Carlsbad, CA, USA). All basal culture media were supplemented with 10% fetal bovine serum and 1% penicillin/streptomycin (GIBCO, Invitrogen, Carlsbad, CA, USA). Cells were maintained in an incubator at 37 °C with 5% CO2. All PCa cell lines were routinely tested for *Mycoplasma* spp. contamination (PCR Mycoplasma Detection Set, Clontech Laboratories).

To reverse DNA methylation effect in the cell lines, we used 1 μM of the DNA methyltransferases inhibitor 5-aza-2-deoxycytidine (5-Aza-CdR; Sigma-Aldrich, Schnelldorf, Germany) alone or in combination 0.5 μM histone deacetylase inhibitor trichostatin A (TSA; Sigma-Aldrich, Schnelldorf, Germany). After 72 h, cells were harvested and RNA extracted.

### Pre-miRNA and anti-miRNA transfections

To inhibit miR-130b and miR-301b, single-stranded nucleic acids designed to specifically bind and inhibit endogenous miRNA (miR-130b Inhibitor, product ID: AM10777; miR-301b Inhibitor, product ID: AM12929, Ambion) were used. Anti-miR-130b and Anti-miR-301b were transfected as follows: in LNCaP, 25 and 50 nM, respectively; DU145, each at 50 nM; and PC3, 50 and 70 nM, respectively.

MiR-130b and miR-301b overexpression were accomplished through commercially available synthetic precursor miRNAs (pre-miR-130b, product ID: PM10777; pre-miR-301b, product ID: PM12929, Ambion), each transfected at 20 nM. Transfections were performed using Oligofectamine (Invitrogen), per manufacturer instructions.

### Viability assay

Cell viability was evaluated by MTT assay. Briefly, PCa cells were seeded onto 96-well flat bottomed culture plates, allowed to adhere overnight and transfected 24 h later (number of cells plated before transfection: LNCaP: 10000 cells/well; DU145: 4000 cells/well; PC3: 3000 cells/well in 96-well plates). At each time point, 0.5 mg/ml of MTT reagent [3-(4,5-dimethylthiazol-2-yl)-2,5-diphenyl-tetrazolium bromide] was added to each well, and the plates were incubated in the dark for 1 h at 37 °C. Formazan crystals were then dissolved in DMSO and absorbance was read at 540 nm in a microplate reader (FLUOstar Omega, BMG Labtech, Offenburg, Germany), subtracting the background, at 630 nm. Three replicates for each condition were performed, and at least three independent experiments were carried out. Measurements were performed 24, 48 and 72 h post-miRNA manipulation.

### Apoptosis evaluation

Evaluation of apoptosis was performed using APOPercentage apoptosis assay kit (Biocolor Ltd., Belfast, Northern Ireland) according to the manufacturer’s instructions. PCa cells were seeded onto 24-well plates (LNCaP: 50,000 cells/well, DU145 and PC3: 30,000 cells/well) and 24 h later were transfected. Apoptotic cells were assessed at the end of day 3 (72 h after transfection), in a FLUOstar Omega microplate reader at 550 nm and the background subtracted at 620 nm. The results were normalized to number of viable cell determined in MTT assay according to the following formula: OD of apoptosis assay at 72 h/OD of MTT at 72 h.

### Cell cycle analysis

Cell cycle distribution of PC3 cells was determined by flow cytometry. Briefly, 72 h after transfection (150,000 cells/well at day 0, in 6-well plates), 5 × 10^5^ harvested cells were fixed overnight at 4 °C with 70% cold ethanol. After washing with cold PBS, cells were re-suspended in Propidium Iodide Solution (Cytognos S.L, Salamanca, Spain) and incubated for 30 min at room temperature. All cells were then measured on a Cytomics FC500 flow cytometer (Beckman Coulter, Fullerton, CA, USA) and analysed using Modfit LT (Verity Software House, Inc., Topshan, ME, USA).

### Single cell gel electrophoresis (comet assay)

Seventy-two hours after transfection (150,000 cells/well at Day 0, in 6-well plates), 50,000 cells were harvested by trypsinization, washed in PBS and re-suspended in 75 μl of low-melting point agarose (Invitrogen, Carlsbad, CA, USA). This suspension was then applied on top of the base layer consisting of normal-melting point agarose in a slide, after which it polymerized for 10 min at 4 °C. The slides were then immersed in lysis solution (2.5 M NaCl, 100 mM Na_2_EDTA, 10 mM Tris Base and 1% Triton X-100) at 4 °C during 2 h in the dark. To allow DNA to unwind, slides were posteriorly incubated in an alkaline electrophoresis buffer (300 mM NaOH, 1 mM Na2EDTA, pH = 13) for 40 min at 4 °C. Electrophoresis was accomplished on a horizontal electrophoresis platform at 4 °C for 20 min at 15 V. Subsequently, they were incubated in a neutralization buffer (Tris–HCl; pH = 7.5) for 10 min. After fixation with 100% ethanol, slides were stained with Sybr Green® (Life Technologies, Foster City, CA, USA) and DNA damage was evaluated under a fluorescent microscope. At least three independent experiments were performed for each condition. The DNA damaging effect in terms of DNA fragmentation was determined by measuring four parameters, that included tail moment, tail length, percentage of DNA in tail of the comet, and 50 DNA-damaged cells were counted at least, for each condition.

### Cell invasion assay

Cell invasion was determined using BD BioCoat Matrigel Invasion Chamber (BD Biosciences, Franklin Lakes, NJ, USA). Both cell lines were transfected with miRNA molecules for 72 h. Then, 5 × 10^4^ cells/mL of PC3 cells were added to the upper chamber. After 44 h (LNCaP) or 20 h (PC3), the membrane bottom containing invading cells was fixed in methanol, washed in PBS and stained with DAPI (Vector Laboratories, Burlingame, CA). All invading cells were counted under a fluorescence microscope. Three independent experiments were performed for each condition.

### Transcriptomic evaluation of altered genes following cluster miR-130b~301b manipulation

Cells (LNCaP: 400,000 cells/well, DU145: 200,000 cells/well and PC3: 150,000 cells/well) were plated in 6-well, in the day before transfection. Cells were collected 72 h post-transfection and RNA was extracted and used as template for cDNA synthesis. RT-qPCR was performed as previously described.

### Western blot

One hundred fifty thousand cells per well were plated before transfection; 72 h post-transfection, cell lysates were separated on 4–20% Mini-PROTEAN TGXPrecast Gel at 120 V and transferred onto PVDF membrane using semi-dry transfer. The membrane was incubated for 1 h in blocking buffer (5% non-fat dry milk) and incubated 2 h, at room temperature, with primary antibodies (Additional file 1: Table S3). Blots were developed using Immun-Star WesternC Chemiluminescent kit (Bio-Rad, Hercules, CA, USA).

### Morphometric analysis

Cell morphology was examined 72 h after transfection using a digital camera connected with Olympus phase-contrast microscope. The cell area and sphericity were determined with the Olympus cellSens Dimension software (Olympus Corporation, Shinjuku, Japan) using the freehand polygon tool.

### TCGA data in prostate cancer patients

Data on mRNA expression and clinical information (when available) from PCa and matched normal patient samples deposited in The Cancer Genome Atlas (TCGA) was retrieved. mRNA expression data from samples hybridized at University of North Carolina, Lineberger Comprehensive Cancer Center, using Illumina HiSeq 2000 mRNA Sequencing version 2, were downloaded from TCGA data matrix (https://gdc-portal.nci.nih.gov/projects/TCGA-PRAD), including 497 PCa and 52 matched normal [12]. To prevent duplicates, when there was more than one portion per patient, median values were used. The provided value was pre-processed and normalized according to ‘level 3’ specifications of TCGA (see https://gdc-portal.nci.nih.gov/ for details). Clinical data of each patient was provided by Biospecimen Core Resources (BCRs). Data is available for download through TCGA data matrix (https://gdc.cancer.gov/gdc-tcga-data-access-matrix-users).

### Statistical analysis

For group comparisons analysis, non-parametric tests (Kruskal-Wallis and Mann-Whitney *U* test) were used. For in vitro assays, comparisons between two groups were performed using the Mann-Whitney *U* test. Data are shown as mean ± s.d., unless otherwise specified. Student’s *t* tests were used for invasion assays. All statistical tests were two-sided. Statistical analysis was carried out using Graph Pad Prism version 5. Significance level was set at *p* < 0.05.

## Results

### Identification of a miRNAs subset targeted by DNA methylation in prostate cancer

**Fig. 1:**
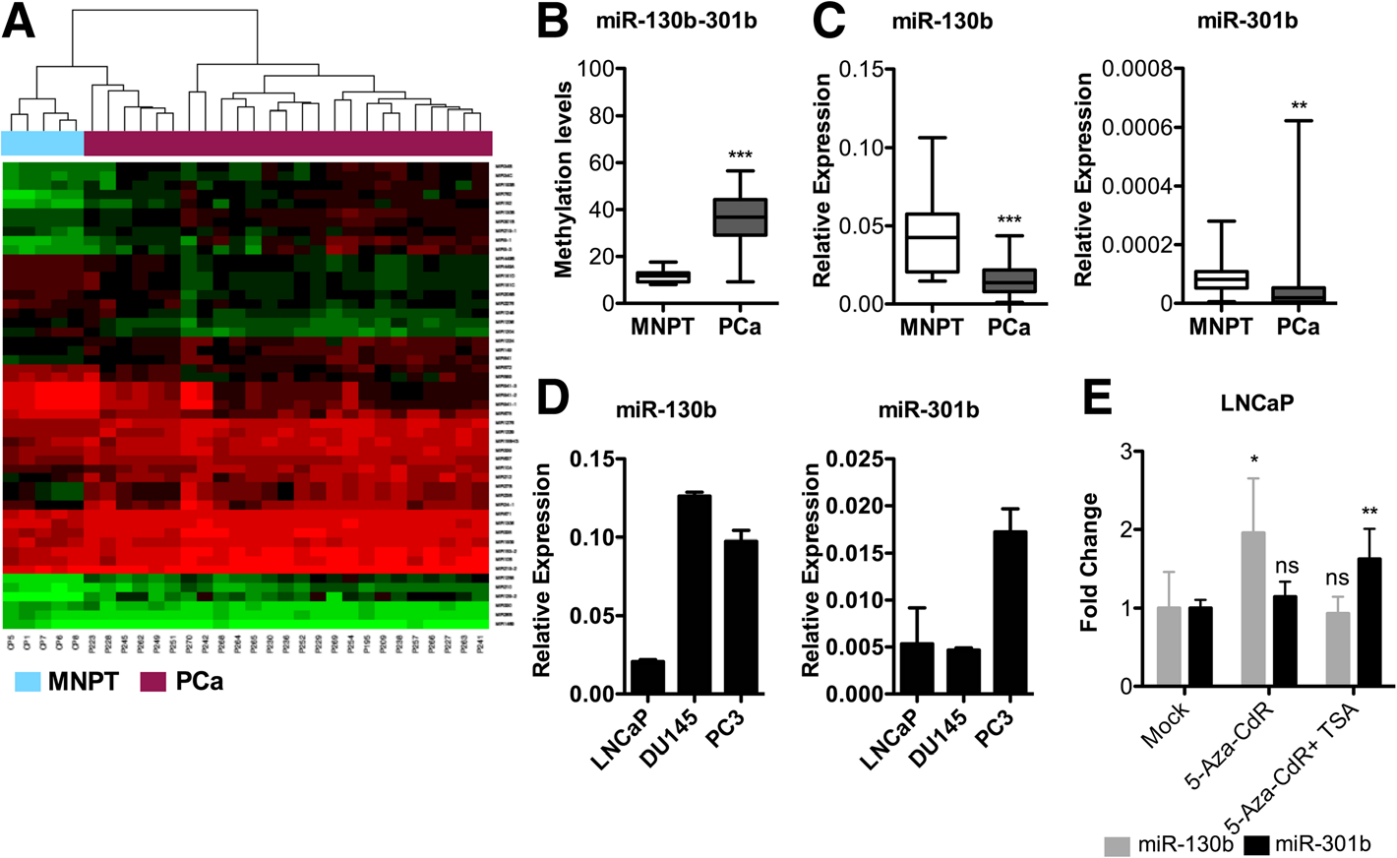
Differentially methylated microRNAs in prostate cancer. **a** Unsupervised hierarchical clustering of microRNAs’ promoters displaying significant alterations in DNA methylation as determined by Infinium HumanMethylation450 BeadChip in 25 prostate cancer (PCa) and 5 morphologically normal prostate tissue (MNPT) samples. Overall, 51 miRNA promoters were differentially methylated in PCa versus MNPT. **b** Validation of miR-130b~301b by pyrosequencing and (**c**) by RT-qPCR in 111 primary PCa and 14 MNPT cases, indicated that promoter hypermethylation was associated with miR-130b~301b downregulation. **d** LNCaP, DU145 and PC3 cell lines retain basal expression of miR-130b and miR-301b. **e** Reversal of DNA methylation in LNCaP cells using 5-aza-2-deoxycytidine (5-AZA-CdR) increased the expression of miR-130b and, in combination, with TSA augmented miR-301b expression. Mann-Whitney *U* test: *p < 0.05, **p < 0.01, ***p < 0.001

We sought to identify specific differentially methylated miRNA loci between PCa and MNPT (Additional file 2: Figure S1A). The DNA methylation analysis was conducted using the Infinium HumanMethylation450 BeadChip (450 k array), a high-density DNA methylation array that interrogates ≈485,000 human CpGs. A total of 439 CpG sites located in miRNA gene promoter regions were found to be differentially methylated and were clustered separately (non-parametric Mann-Whitney and Wilcoxon matched pair test were applied). For all analyses, *p* values inferior to 0.05, after FDR correction, were considered statistically significant (Fig. 1a; Table 1; Additional file 2: Figure S1A). Thus, 51 differentially methylated miRNA promoters in PCa were identified (Fig. 1a, Additional file 2: Figure S1B) and mapped to 19 chromosomes. Chromosomes 19 (*n* = 6), 11 and 7 (*n* = 5) and 2 (n = 4) were the most enriched genomic locations for differential methylation (Additional file 2: Figure S1C). Simultaneously, we identified several hypomethylated candidates, including miR-181c~181d and miR-449a~449b clusters. In the hypermethylated branch, our dataset disclosed previously unreported miRNA-promoters, including miR-130b~301b cluster, miR-149, miR-212, miR-10a, miR-152, miR-210 and miR-129-2. Consistent with previous observations, we confirmed hypermethylation of miR-193b, miR-9 family and miR-34b-34c cluster (Additional file 2: Figure S1B). Gene Ontology (GO, Additional file 1: Table S4) revealed that putative targets of this subset of miRNAs dynamically regulated by DNA methylation are involved in critical pathways including ‘sister chromatid segregation’, ‘regulation of double-strand break repair’, ‘posttranscriptional gene silencing by RNA’, ‘regulation of adaptive immune response’, ‘G1 DNA damage checkpoint’ or ‘DNA-templated transcription’. Strikingly, GO analysis also disclosed that the putative targets of this miRNA panel were also involved in ‘hippo signalling’ and ‘prostate gland growth’, indicating a critical role in normal prostate biology. Based on β-values for DNA methylation levels, miR-130b~301b cluster ranked first (Table 1) and was selected for subsequent validation in a larger cohort.

**Table 1:** Top 10 of differentially methylated miRNAs in prostate cancer

CHR	miRNA	Coordinates (GRCh37.p5)	RELATION CPG ISLAND	miRBase/MirgeneDB [34]	Gene family	Clustered miRNA	Methylation frequency (array)
22	MIR130B;MIR301B	chr22: 22007593-22007674 [+]	S_Shore	Ok/ok	MIR130	MIR301B/130B	0.76
17	MIR152	chr17: 46114527-46114613 [−]	Island	Ok/ok	MIR148		0.72
2	MIR1258	chr2: 180725563-180725635 [−]	S_Shore	Ok/–	MIR1258		0.68
16	MIR762	chr16: 30905224-30905306 [+]	N_Shore	Ok/–	MIR762		0.52
11	MIR34B;MIR34C	chr11: 111384164-111384240 [+]	Island	Ok/ok	MIR34	MIR34B/34C	0.44
15	MIR9-3	chr15: 89911248-89911337 [+]	N_Shore	Ok/ok	MIR9		0.44
1	MIR9-1	chr1: 156390133-156390221 [−]	N_Shore	Ok/ok	MIR9		0.36
11	MIR129-2	chr11: 43602944-43603033 [+]	Island	Ok/ok			0.36
6	MIR219-1	chr6: 33175612-33175721 [+]	N_Shore	Ok/ok	MIR219		0.32
11	MIR210	chr11: 568089-568198 [−]	Island	Ok/ok	MIR210		0.32

### Validation of HumanMethylation450 BeadChip by pyrosequencing

Validation of miR-130b~301b cluster results was accomplished through pyrosequencing, which confirmed that promoter methylation levels were significantly higher in PCa compared to MNPT (Fig. 1b). Likewise, PCa cell lines DU145, LNCaP and PC3 also demonstrated miR-130b~301b promoter methylation (Additional file 2: Figure S1D).

### DNA methylation associates with miR-130b~301b cluster expression

MiR-130b~301b cluster expression levels were evaluated in a series of 125 prostate tissue samples, using RT-qPCR, and were found to be significantly downregulated in PCa (*p* < 0.0001 for miR-130b; *p* = 0.0014 for miR-301b, Fig. 1c) compared to MNPT. Then, the effect of demethylating drugs was tested, as the PCa cell lines still displayed endogenous expression of miR-130b and miR-301b (Fig. 1d). In LNCaP cells, miR-130b was significantly upregulated after exposure to 5-Aza-CdR, whereas miR-301b was only re-expressed upon combined treatment with 5-Aza-CdR and TSA (Fig. 1e).

### Functional impact of miR-130b~301b cluster expression manipulation in vitro

The phenotypic impact of altered miR-130b~301b cluster expression was assessed in PCa cell lines in which miR-130b~301b cluster expression was detected along with promoter methylation: LNCaP, DU145 and PC3 (Additional file 2: Figure S1D).

**Fig. 2:**
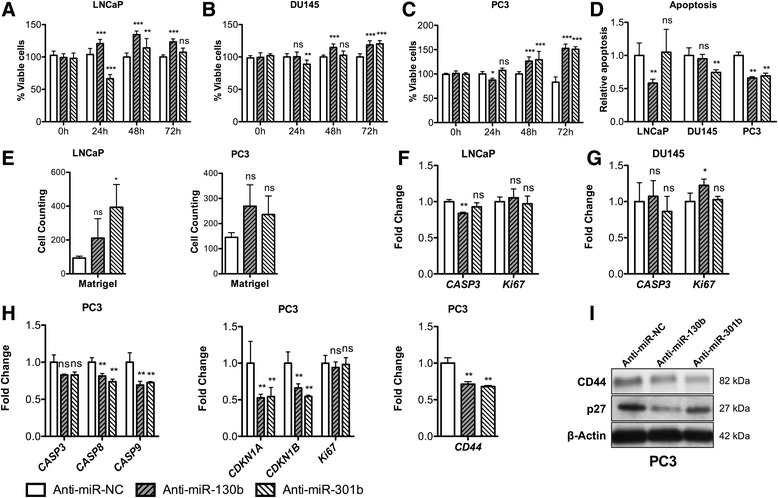
Phenotypic effects induced by blocking endogenous levels of miR-130b or miR-301b in PCa cell lines. **a**–**c** Cell viability measured by MTT assay at different time points and (**d**) apoptosis evaluation 72 h post-transfection for LNCaP, DU145 and PC3 cells, respectively, indicating functional specialization (LNCaP and DU145) or cooperation (PC3) among members of miR-130~301b cluster in PCa cell lines. **e** Invasion assay following anti-miR knockdown of miR-130b or miR-301b using Matrigel coated Boyden chamber assay in LNCaP and PC3 cells, 72 h post-transfection. **f, g** Transcript levels of *CASP3* and *KI67* in LNCaP and DU145 cells, respectively, 72 h after anti-miRNAs transfection. **h** mRNA expression of selected genes involved in cell cycle, apoptosis and invasion in PC3 cells transfected with anti-miRNAs, indicating that both miR-130b and miR-301b knockdown decreased the expression of Caspases (3, 8 and 9) and critical cell cycle check-point regulators. **i** Representative Western blots for CD44 and p27. All data are presented as mean of three independent experiments ± s.d. (*p < 0.05, **p < 0.01, ***p < 0.001)

The impact of endogenous miR-130b~301b blockage was firstly assessed, and the efficiency of silencing was confirmed by RT-qPCR (Additional file 2: Figure S2). In LNCaP cells, anti-miR-130b significantly enhanced growth rate at 72 h (Fig. 2a, *p* < 0.001), whereas anti-miR-301b showed no significant effect. Conversely, at 72 h post-transfection, apoptosis was only decreased in anti-miR-130b transfected LNCaP cells (Fig. 2d, *p* = 0.0043). Importantly, decreased *CASP3* expression levels (Fig. 2f) were consistent with reduced apoptosis. Interestingly, in LNCaP cells, miR-301b knockdown significantly increased invasion capacity. However, for miR-130b silencing, no significant differences were apparent, suggesting that miR-130b is more likely implicated in invasion regulation than miR-301b. In DU145 cells, inhibition of either miRNA significantly increased cell viability (Fig. 2b, *p* < 0.001 for both). Interestingly, the effect of anti-miR-130b was already apparent at 48 h upon transfection (*p* < 0.0001). Although decreased apoptosis was depicted for both conditions, it only reached statistical significance in anti-miR-301b transfected cells (Fig. 2d, *p* = 0.0022). A slight increase in *Ki67* mRNA expression was found upon anti-miR-130b transfection (Fig. 2g, *p* = 0.026). Thus, in DU145 cells, miR-301b seems to be more critical than miR-130b, although the latter might influence cell viability. MiR-130b or miR-301b inhibition in PC3 cells dramatically enhanced cell viability (Fig. 2c, *p* < 0.001). Moreover, increased proliferation was complemented with a significant decrease in apoptosis after anti-miR-130b or anti-miR-301b transfection (Fig. 2d). Remarkably, an apparent effect on cell invasion was observed for miR-130b~301b depleted PCa cells, reaching statistical significance in miR-301b-depleted LNCaP cells (Fig. 2e).

Because phenotypic changes were more apparent in PC3 cells, these were selected for evaluation of expression of several genes involved in relevant signalling pathways. Thus, a significant decrease in *CASP8*, *CASP9, CDKN1A* and *CDKN1B* expression was depicted, whereas *CASP3* and *Ki67* mRNA levels remained unaltered (Fig. 2h). Moreover, a significant reduction in CD44 and p27 expression was also observed, in line with the invasive phenotype induced by anti-miR-130b and anti-miR-301b transfection in PC3 cells (Fig. 2h,i).

### MiR-130b~miR-301b overexpression attenuates the malignant phenotype and promotes MET

**Fig. 3:**
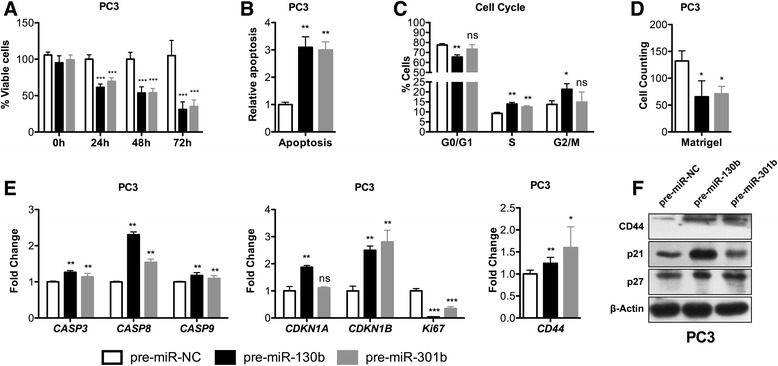
miR-130b and miR-301b overexpression attenuate malignant phenotype of PC3 cells. **a** Cell viability measured by MTT assay indicates that transfection of pre-miR-130b and pre-miR-301b significantly decreased cell viability compared to pre-miR-NC transfected cells. **b** Either pre-miR-130b or pre-miR-301b significantly increased the relative apoptosis levels as determined by the phosphatidylserine-based assay. **c** Cell cycle analysis of PC3 control cells (pre-miR-NC) and PC3 overexpressing pre-miR-130b or pre-miR-301b, indicate that both miR-130b and miR-301b significantly induce cell cycle arrest at S-phase and miR-130b also causes G2/M arrest. **d** Invasion assay in PC3 cells transfected with the pre-miRNAs 72 h before plating in Matrigel-coated Boyden chambers. **e** mRNA expression levels of selected genes involved in apoptosis, cell cycle and invasion support that miR-130b and miR-301b cooperatively reverse the acquisition of malignant features of PC3 cells. **f** Western blot for p21, p27 and CD44 in PC3 cells, depicting selected gene overexpression upon miR-130b or miR-301b overexpression. All data are presented as mean of three independent experiments ± s.d. (*p < 0.05, **p < 0.01, ***p < 0.001)

The phenotypic impact of miR-130b or miR-301b overexpression was tested in PC3 cells. A marked reduction in cell viability (Fig. 3a) and increased apoptosis (Fig. 3b), along with increased caspases expression, especially *CASP8* (Fig. 3e), was observed. Cell cycle analysis by flow cytometry depicted a significant arrest at S phase following miR-130b or miR-301b overexpression and at G2/M phase after miR-130b overexpression (Fig. 3c). These phenotypic alterations were further confirmed by significant decrease in *Ki67* expression and increased *CDKN1A* (p21) and *CDKN1B* (p27) expression, both at mRNA and protein level (Fig. 3e, f). In the TCGA dataset, these findings were confirmed at mRNA level for *CDKN1A* (*p* < 0.01), but not for *CDKN1B*, (Additional file 2: Figure S3), whereas *Ki67* was strongly up-regulated in PCa samples (*p* < 0.0001), as expected. Collectively, these observations indicate that decreased cell viability results from a combined effect of cell cycle arrest and increased apoptosis.

We then hypothesized that miR-130b~301b cluster might inhibit epithelial to mesenchymal transition (EMT) and/or facilitate mesenchymal to epithelial transition (MET) in PCa cells. PC3 cells possess a more mesenchymal-like gene expression profile [13] and phenotype. Moreover, the capacity of cancer cells to migrate and invade is an important requirement for metastasis formation, and both are EMT hallmarks. With this in mind, the effect of miR-130b~301b expression on PC3 cells migration was assessed. Restoration of miR-130b~301b impaired the invasive capacity of PC3 cells (Fig. 3d), whereas the opposite was observed following miR-130b~301b depletion (Fig. 2e). Moreover, miR-130b~301b overexpression was associated with increased CD44 expression, both at mRNA and protein level (Fig. 3e,f), whereas inhibition of miR-130b or miR-301b decreased CD44 expression (Fig. 2h,i). The expression of other genes implicated in EMT was also assessed (Additional file 2: Figure S4) and a differential impact of miR-130b and miR-301b was suggested.

Moreover, miR-130b or miR-301b overexpression caused a shift in PC3 cell morphology towards a more epithelial-like phenotype, compared to wild-type PC3 cells or those with miR-130b or miR-301b depletion, which are more spindled (i.e., more mesenchymal-like, Additional file 2: Figures S4 and S5). These findings suggest that miR-130b and miR-301b facilitate MET, impairing cell migration and invasion.

### Cluster miR-130b~301b induces senescence in PC3 cells

Cellular senescence is a process by which proliferation-competent cells undergo growth arrest, in response to various cellular stresses. Because miR-130b and miR-301b were able to induce cell cycle arrest and decreased cell viability, along with *CDKN1A* and *CDKN1B* overexpression and Ki67 downregulation, a link with cellular senescence was suggested.

**Fig. 4:**
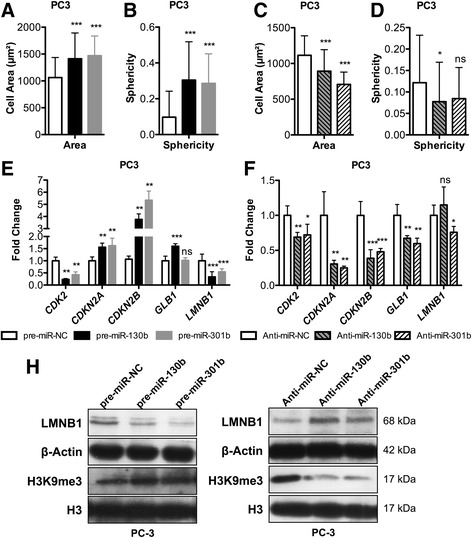
Modulation of miR-130b and miR-301b induces cell senescence. Cell area (**a**) and sphericity (**b**) were increased upon miR-130b or miR-301b overexpression and compared to the pre-miR-NC transfected PC3 cells. Cell area (**c**) and sphericity (**d**) decreased with anti-microRNAs knockdown of miR-130b or miR-301b, indicating a more fibroblast-like phenotype. **e** RT-qPCR confirms transcriptional signature associated with promotion of cellular senescence after forced expression of miR-130b or miR-301b. **f** attenuation of senescent phenotype following inhibition of endogenous levels of each miRNA. **h** Western blot shows that LMNB1 levels are downregulated when miR-130b or miR-301b are overexpressed, concomitantly with locus-specific H3K9me3 increase. All data are presented as mean of three independent experiments ± s.d. (*p < 0.05, **p < 0.01, ***p < 0.001)

Because senescent cells undergo cell size increase, this characteristic was evaluated upon miR-130b or miR-301b re-expression in PC3 cells. Morphometric analysis (Additional file 2: Figure S5) disclosed a significant increase in cell area (approximately 50%), compared to scramble cells (Fig. 4a, *p* < 0.0001), with a significant increase in sphericity, as well (*p* < 0.0001, Fig. 4b). Conversely, a significant decrease in cell area was apparent when endogenous miR-130b or miR-301b were depleted (Fig. 4c; Additional file 2: Figure S6), whereas a significant decrease in sphericity was depicted for miR-130b only (Fig. 4d, Additional file 2: Figure S6). Then, expression of other senescence-associated genes was evaluated. Transfection of miR-130b or miR-301b was associated with significant upregulation of tumour suppressor genes *CDKN2A* (p16) and, more dramatically, *CDKN2B* (p15) (Fig. 4e), alongside with downregulation of *LMNB1*, a marker of cellular senescence (Fig. 4e), which was confirmed at protein level (Fig. 4h). Nevertheless, increased *β-galactosidase* (*GLB1*) mRNA levels were only apparent upon miR-130b expression (Fig. 4e). Globally, the opposite trend was observed after endogenous miR-130b or miR-301b depletion (Fig. 4f–h), although a few exceptions were apparent, including *CDK2* downregulation, at transcript level.

Formation of senescence-associated heterochromatic foci (SAHF), specifically enriched for H3K9me3, has been implicated in cellular senescence. Interestingly, following pre-miR-130b transfection, an increase in H3K9me3 was depicted, whereas anti-miR-130b and anti-miR-301b transfections were associated with H3K9me3 decrease (Fig. 4h).

In TCGA dataset (Additional file 2: Figure S3), overexpression of *LMNB1* (*p* = 3.32 × 10^−10^) and down-regulation of *CDKN2B* (*p* = 0.000218) was depicted in PCa tissue samples, mimicking to some extent the pattern observed following endogenous miR-130b or miR-301b depletion. Nevertheless, whether *LMNB1* reduction is caused by senescence or is promoted by a direct interaction of miR-130b or miR-301b with *LMNB1*-3′ UTR (Additional file 2: Figure S7) remains unanswered.

### miR-130b~301b induces SASP expression

**Fig. 5:**
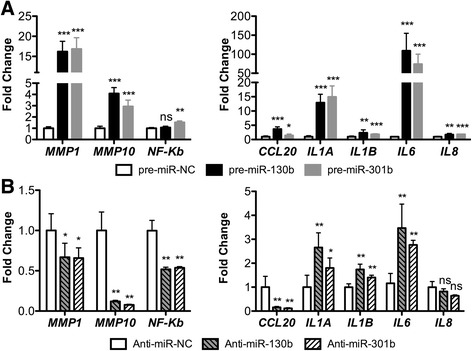
miR-130b and miR-301b overexpression dramatically alters SASP expression in PC3 cells. **a** Comparing miR-130b or miR-301b overexpression with pre-miR-NC control, a global increase in mRNA levels of most SASP-related genes was depicted. This signature suggests that miR-130b or miR-301b-induced SASP reinforces senescence through autocrine mechanisms. **b** Comparison of miR-130b or miR-301b endogenous blockade with anti-miR-NC control revealed a decrease (e.g. *MMP10*) or minimal increase (e.g. *IL1A*) in mRNA expression of some genes. This suggests paracrine activity of SASP when miR-130b or miR-301b are inhibited in prostate cancer cells. All data are presented as mean of three independent experiments ± s.d. (*p < 0.05, **p < 0.01, ***p < 0.001)

The secretome of senescent cells is complex, consisting of a range of cytokines, chemokines and proteases, among others. To further confirm our previous findings, we sought to analyse some elements of the senescence-associated secretory phenotype (SASP), as these constitute phenotypic and molecular markers of senescence [14]. Thus, *MMP1*, *MMP10*, *CCL20*, *IL1A*, *IL1B*, *IL6* and *IL8* expression was assessed. Globally, miR-130b or miR-301b overexpression associated with increased expression of all genes tested, whereas anti-miR-130b or anti-miR-301b transfection associated with decreased *MMP1*, *MMP10* and *CCL20* expression, alongside with *IL1A*, *IL1B* and *IL6* overexpression, although at a much smaller magnitude compared to miR-130b or miR-301b overexpression (Fig. 5a, b).

In TCGA dataset, PCa tissue samples displayed significantly lower *IL1A*, *IL1B* and *IL6* expression levels compared to normal prostate tissues (Additional file 2: Figure S3).

### SASP activation is induced by DNA damage

**Fig. 6:**
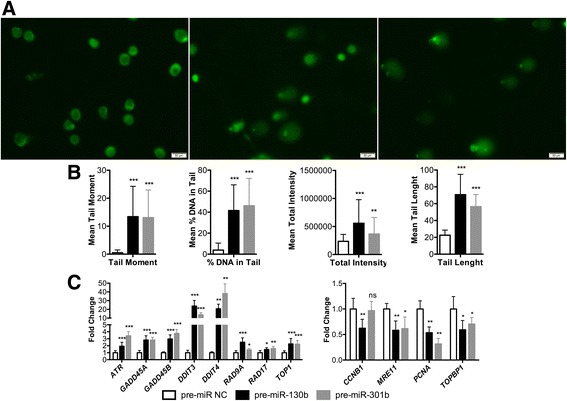
miR-130b and miR-301b impair DNA-damage signalling pathway. **a** Comet assay immunofluorescence images of PC3 cells transfected with pre-miRNAs and counterstained with Syber Green, depicting DNA-damage associated morphology. **b** Graphic representation of parameters analysed in the assay, supporting that both miR-130b and miR-301b overexpression induce DNA damage in PC3 cells. **c** RT-qPCR evaluation of multiple genes involved in DNA-damage response pathway. All data are presented as mean of three independent experiments ± s.d. (*p < 0.05, **p < 0.01, ***p < 0.001)

In senescent cells, the control of secretome is achieved at many levels, from transcriptional regulation to autocrine feedback loops, but persistent DNA damage response (DDR) appears to be critical for regulation of SASP [15]. We thus hypothesized that miR-130b and miR-301b might influence DNA damage responses and genomic instability during senescence. Upon pre-miR-130b or pre-miR-301b overexpression in PC3 cells, a significant increase in DNA damage was depicted, using the comet assay (Fig. 6a, b), especially in tail moment (an index of induced DNA damage) and in the percentage of DNA in the tail. Subsequently, expression of genes involved in DDR was evaluated and a significant increase in two DNA damage inducible transcripts, *DDIT3* (that positively regulates IL6 and IL8) and *DDIT4* was found (Fig. 6c). Moreover, *ATR*, a DNA-damage detector, was also upregulated. Strikingly, the growth arrest and DNA-damage-inducible proteins *GADD45A* and *GADD45B* were significantly overexpressed, as well as *RAD9A* and *RAD17* (Fig. 6c). Conversely, *PCNA* (a cell proliferation marker) was among the downregulated genes.

## Discussion

The intense research on the epigenetics field led to the discovery that genes encoding miRNAs were epigenetically silenced through DNA methylation [1].

Because the miR-130b~301b cluster ranked first among all hypermethylated miRNA promoters in our dataset and, to the best of our knowledge, had not been previously reported in PCa, it was selected for subsequent validation and functional characterization. Pyrosequencing of a large number of primary PCa and normal prostate tissues, confirmed that miR-130b~301b cluster promoter methylation levels were significantly higher in the former, whereas the opposite was apparent for expression levels of both miRNAs, thus prompting an association between aberrant promoter methylation and expression downregulation in PCa. This was further confirmed in vitro as PCa cell lines disclosed increased expression levels after exposure to a demethylating agent, either alone or in combination with TSA. Importantly, these findings are comparable to those reported for miR-193b, miR-34b~34c and miR-23b~27b~24-1 cluster [16–18], confirming that aberrant promoter methylation is, indeed, the mechanism underlying miR-130b~301b cluster downregulation in PCa.

Concerning the functional characterization of these findings, it should be emphasized that miR-130b and miR-301b are members of a miRNA family which is deregulated in several cancer types, acting either as onco-miRs or tumour-suppressive miRs. Indeed, a tumour-suppressive role for miR-130b in PCa has been proposed (although the mechanism underlying its downregulation was not disclosed), counteracting metastasis formation through MMP2 downregulation [19]. Nevertheless, another report implicated miR-130b in tumorigenic reprogramming of adipose tissue-derived stem cells in PCa patients, acting as oncomir [20]. Furthermore, the role of miR-301b in PCa remains elusive, although it appears to be induced under hypoxia and target *NDRG2* [21, 22]. Interestingly, the functional assays confirmed the tumour-suppressive action of miR-130b and miR-301b. In both cases, miRNA overexpression reduced cell viability, induced apoptotic cell death and irreversibly activated the cell cycle arrest program DNA damage-induced senescence.

Phenotypic alterations were supported at molecular level, as restored expression of both miR-130b and miR-301b significantly increased the expression of genes acting as checkpoint sensors, required for effective tumour suppression. It is not clear whether these alterations directly result from miRNA-mRNA interactions at 5′ UTR or promoter [23], or from the naive output of tumour-suppression. It might be speculated that both miR-130b and miR-301b interact with other regulatory elements and consequently enhance transcription or translation of those genes [23]. Indeed, it has been hypothesized that many miRNAs have evolved to act not as genetic switches of specific pathways or individual targets but rather to modulate expression of large gene networks [24]. Moreover, it should be recalled that due to the seed sequence similarity among miRNAs of the same family, targets from the same miRNAs cluster may be shared, although specific targets might also exist, as result of other base pairing determinants in addition to seed sequence [25]. This may explain why restoration of either miR-130b or miR-301b basically had the same functional impact. Nonetheless, the magnitude of the effect may be different, as demonstrated for several target genes, including *Ki67* and *CASP3*. Thus, different functional specialization of miR-130b and miR-301b is proposed.

Our data suggest that miR-130b~301b cluster might counteract malignant transformation of prostate epithelial cells through impairment of EMT, favouring MET instead. This was apparent not only morphologically, as PC3 cells exhibited a more epithelial phenotype upon miR-130b or miR-301b overexpression, but also at molecular level, through increased expression of several genes, including *CD44*. Interestingly, *CD44* downregulation was depicted following transfection with anti-miR-130b or anti-miR-301b. Decreased *CD44* expression has been associated with a more aggressive PCa phenotype, due to its association with higher grade and pathological stage, correlating with biochemical recurrence and tumour relapse [26]. Our observations are in line with these findings, although the mechanism by which the miR-130b~301b cluster influences *CD44* expression requires clarification. Nevertheless, it should be emphasized that the impact of miR-130b and miR-301b on EMT-related genes seems to differ, as illustrated by the almost opposite expression patterns of *TGFB3* and *WNT5A*. Yet, because no double transfection experiments were conducted (as all were transient transfections), the net result of miR-130b~301b cluster downregulation cannot be determined.

An interesting and novel finding was the link between miR-130b~301b cluster and cellular senescence. This process induces cell cycle and cell growth arrest, and it may counteract tumour formation [27]. Accumulation of DNA damage is a common basis for senescence, preventing genomic instability [28]. Senescent cells display cell size increase and a more flattened shape, as well as increased p53, *CDKN2A* (p16), *CDKN1A* (p21) and *CDKN1B* (p27) expression, and *LMNB1* downregulation [29, 30]. Remarkably, the same gene expression pattern was observed upon miR-130b or miR-301b overexpression, whereas miR-130b or miR-301b depletion had the opposite effect, suggesting that miR-130b or miR-301b downregulation might allow for senescence bypass. Our observations are also in line with previous reports correlating *LMNB1* reduction (particularly from H3K9me3 regions) and spatial repositioning of perinuclear heterochromatin (H3K9me3-enriched) and SAHF formation [31]. These findings are further supported by induction of SASP upon miR-130b or miR-301b overexpression. Interestingly, in oncogene-induced senescence (OIS), SASP is regulated by persistent DDR [32, 33]. We found that miR-130b or miR-301b overexpression stimulated the expression of genes involved in DDR as well as in DNA repair, suggesting that miR-130b~301b cluster downregulation might impair OIS and foster malignant transformation of prostate cells.

## Conclusions

In conclusion, we found novel miRNAs deregulated through aberrant promoter methylation in PCa. In particular, the miR-130b~301b cluster displays a tumour-suppressive profile and its downregulation might fuel malignant transformation and tumour progression through facilitation of EMT and bypass of cellular senescence.

## Additional files

Additional file 1: Table S1. Clinical and pathological data of the patients included in the study. **Table S2.** Primers used in the study. **Table S3.** Description of the antibodies used. **Table S4.** Gene Ontology terms for the altered putative miRNA targets. (DOCX 268 kb)
Additional file 2: Figure S1. DNA methylation changes in microRNAs´ promoters in prostate cancer (PCa), determined by Infinium HumanMethylation450 BeadChip in 25 PCa tissues and 5 morphologically normal prostate tissue (MNPT) samples. (A) Schematic representation of the DNA methylation mapping approach used to identify new aberrantly methylated miRNAs. (B) *β* values from representative microRNAs. (C) Genomic location of the differentially methylated microRNAs. (D) DNA methylation levels determined by pyrosequencing in PCa cell lines, showing that all cell lines tested display aberrant DNA methylation in the promoter of miR-130b~301b cluster. (**p* < 0.05, ***p* < 0.01, ****p* < 0.001). **Figure S2.** Confirmation of miR-130b and miR-301b expression levels by RT-qPCR. (A–C) miR-130b or miR-301b expression levels after transfection with anti-miR-NC, anti-miR-130b and, anti-miR-301b in LNCaP, DU145 and, PC3, respectively. (D) Overexpression of miR-130b and miR-310b in PC3 cells. The analyses were conducted 72 h post-transfections. All data are presented as mean of three independent experiments ± s.d. (**p* < 0.05, ***p* < 0.01, ****p* < 0.001). **Figure S3.** Cross-validation of deregulated genes upon cluster miR-130b-301b manipulation in the TCGA cohort. Boxplot depiction of the cancer versus normal differentially expressed mRNAs among the TCGA prostate RNA-seq cohort. Green and red squares refer to down-regulated and overexpressed genes in PCa versus NAT samples, respectively. Each point represents one RNA-seq tissue sample. **Figure S4.** RT-qPCR expression changes in multiple genes involved in invasion and epithelial to mesenchymal transition (EMT), suggesting functional specialization among members of miR-130b-301b polycistron. Gene expression patterns by (A) induction of pre-miR-130b or pre-miR-301b or (B) after endogenous levels blocking. The analyses were conducted 72 h post-transfections. All data is presented as mean of three independent experiments ± s.d. (**p* < 0.05, ***p* < 0.01, ****p* < 0.001). **Figure S5.** Morphological alterations in PC3 cells after miR-130b or miR-301b overexpression. Transfection of (A) pre-miR-NC, (B) pre-miR-130b or (C) pre-miR-301b. The restoration of miR-130b or miR-301b expression induced cell polarization and epithelial-like phenotype, suggesting a mesenchymal to epithelial transition. **Figure S6.** Morphological alterations in PC3 cells upon miR-130b or miR-301b knockdown. (A) PC3 cells transfected with anti-miR-NC, (B) anti-miR-130b or (C) anti-miR-301b. Inhibition of endogenous miR-130b or miR-301b caused PC3 cells to acquire a more pronounced fibroblast-like morphology, compatible with a mesenchymal-type phenotype. **Figure S7.** *LMNB1* 3′ UTR putative binding sites for miR-130b and miR-301b. (PDF 2147 kb)

## Abbreviations

5-Aza-CdR: 5-Aza-2-deoxycytidine
AGO: Argonaute proteins
DDR: DNA damage response
DMNT: DNA methyltransferase
EMT: Epithelial to mesenchymal transition
MET: Mesenchymal to epithelial transition
miRNA: MicroRNA
MNPT: Morphologically normal prostate tissues
MTT: 3-(4,5-Dimethylthiazol-2-yl)-2,5-diphenyl-tetrazolium bromide
OIS: Oncogene-induced senescence
PCa: Prostate cancer
SAHF: Senescence-associated heterochromatic foci
SASP: Senescence-associated secretory phenotype
SNPs: Single-nucleotide polymorphisms
TCGA: The Cancer Genome Atlas
TSA: Trichostatin A
TSS: Transcription-start sites
UTR: Untranslated region
