# Supplementary material for: Downregulation of miR-130b~301b cluster is mediated by aberrant promoter methylation and impairs cellular senescence in prostate cancer
Source: J Hematol Oncol. 2017 Feb 6;10:43. doi: 10.1186/s13045-017-0415-1 (PMC5294724; doi:10.1186/s13045-017-0415-1)
Supplement: Additional file 1: Table S1. — Clinical and pathological data of the patients included in the study. Table S2. Primers used in the study. Table S3. Description of the antibodies used. Table S4. Gene Ontology terms for the altered putative miRNA targets. (DOCX 268 kb) [file 13045_2017_415_MOESM1_ESM.docx]

**Supplementary Information**

**Ramalho-Carvalho *et* al: Downregulation of miR-130b~301b cluster is mediated by aberrant promoter methylation and impairs cellular senescence in prostate cancer.**

**Supplementary Table S1 –** Clinical and pathological data of the patients included in the study.

**Supplementary Table S2** – Primers used in the study.

**Supplementary Table S3** – Description of the antibodies used.

**Supplementary Table S4** – Gene Ontology terms for the altered putative miRNA targets.

**Supplementary Figures 1-7 and captions**

**Supplementary Tables**

**Supplementary Table S1 –** Clinical and pathological data of the patients included in this study.

| **Clinico-pathological Features** | **MNPT** | **PCa** |
| --- | --- | --- |
| **Patients, n** | 14 | 111 |
| **Median age, years*** | 65 (49 - 80) | 64 (49 - 74) |
| **PSA (ng/mL), median (range)*** | *n.a.* | 8.1 (3.4 - 23) |
| **Stage pT2** | *n.a.* | 61 (55%) |
| **Stage pT3** | *n.a.* | 50 (45%) |
| **Gleason score < 7** | *n.a.* | 31 (27.9%) |
| **Gleason score = 7** | *n.a.* | 66 (59.5%) |
| **Gleason score > 7** | *n.a.* | 14 (12.6%) |

**Supplementary table S2 – Primers used in the study**

| **Primer** | **5’- Sequence – 3’** | **Figure** | |
| --- | --- | --- | --- |
| miR129-2-pyro_F | GTTGGGGAGATTTAGTTTGT | | 1 |
| miR129-2-pyro_R | (Btn)CTATTAAATTATATACAACAAACCCAAACC | | 1 |
| miR129-2-pyro_S | GGAGATATTTTGGGTTGA | | 1 |
| miR130_301b-pyro_F | GTTAGTTTTTGTTTYGGGATTGGAATAATT | | 1 |
| miR130_301b-pyro_R | [Btn]ACAAAAAAAACCCCACCA | | 1 |
| miR130_301b-pyro_S | TTGGGGTTTTTTTTTTATTTTAAT | | 1 |
| miR152-pyro_F | GGGTTTAAGTTTTGTTATGTATTGATTGT | 1 | |
| miR152-pyro_R | [Btn]AAATCCAACCCRACCAAAAATCAACTA | 1 | |
| miR152-pyro_S | GAGTYGGAGTGTATTATAGAATT | 1 | |
| BGUS_qPCR_F | CTCATTTGGAATTTTGCCGATT |  | |
| BGUS_qPCR_R | CCGAGTGAAGATCCCCTTTTTA |  | |
| CASP3_qPCR_F | GTGAGGAGTTAGCGAGCCC | 2; 3 | |
| CASP3_qPCR_R | ACCTTTATTAATGAGAATGGGGGA | 2; 3 | |
| CASP8_qPCR_F | TCTGCCTACAGGTTCCACTT | 2; 3 | |
| CASP8_qPCR_R | TAGATGATCGACCCTCCGCC | 2; 3 | |
| CASP9_qPCR_F | GGAAGAGCTGCAGGTGGAC | 2; 3 | |
| CASP9_qPCR_R | CCTGCCCGCTGGATGTC | 2; 3 | |
| KI67_qPCR_F | GGACTTTGGGTGCGACTTGA | 2; 3 | |
| KI67_qPCR_R | ACAACTCTTCCACTGGGACG | 2; 3 | |
| CDKN1A_qPCR_F | TGCCGAAGTCAGTTCCTTGT | 2; 3 | |
| CDKN1A_qPCR_R | GTTCTGACATGGCGCCTCC | 2; 3 | |
| CDKN1B_qPCR_F | TTGGTGGACCCAAAGACTGA | 2; 3 | |
| CDKN1B_qPCR_R | GAAGAATCGTCGGTTGCAGG | 2; 3 | |
| CDK2_qPCR_F | GGCACGTACGGAGTTGTGTA | 4 | |
| CDK2_qPCR_R | ACCCTCAGTCTCAGTGTCCA | 4 | |
| CDKN2A_qPCR_F | GAAGGTCCCTCAGACATCCCC | 4 | |
| CDKN2A_qPCR_R | CCCTGTAGGACCTTCGGTGAC | 4 | |
| CDKN2B_qPCR_F | GGGACTAGTGGAGAAGGTGC | 4 | |
| CDKN2B_qPCR_R | CATCATCATGACCTGGATCGC | 4 | |
| qPCR_GLB1_R | gaagatggctgggctgaa | 4 | |
| qPCR_GLB1_F | tccacatcatggtcctcaga | 4 | |
| LMNB1_qPCR_F1 | CGACCAGCTGCTCCTCAACTAT | 4 | |
| LMNB1_qPCR_R1 | TTCTCGAAGCTTGATCTGGGC | 4 | |
| LMNB1_qPCR_F2 | GCGCTCGACGACACGG | 4 | |
| LMNB1_qPCR_R2 | TCCTTCTTAGCATAGTTGAGGAGC | 4 | |
| MMP1_qPCR_F | gctaacctttgatgctataactacga | 5 | |
| MMP1_qPCR_R | ggatttgtgcgcatgtagaa | 5 | |
| MMP10_qPCR_F | AGTTTGGCTCATGCCTACCC | 5 | |
| MMP10_qPCR_R | TTGGTGCCTGATGCATCTTCT | 5 | |
| NFKB1_qPCR_F | GCTTAGGAGGGAGAGCCCA | 5 | |
| NFKB1_qPCR_R | CTGCCATTCTGAAGCCGGG | 5 | |
| CCL20_qPCR_F | CCAAGAGTTTGCTCCTGGCT | 5 | |
| CCL20_qPCR_R | TTGCTTGCTTCTGATTCGCC | 5 | |
| IL1A_qPCR_F | GACTCAGGCTTAAGCTGCCA | 5 | |
| IL1A_qPCR_R | TGGCCATCTTGACTTCTTTGC | 5 | |
| IL1B_qPCR_F | GCCAATCTTCATTGCTCAAGTGT | 5 | |
| IL1B_qPCR_R | AGCCATCATTTCACTGGCGA | 5 | |
| IL6_qPCR_F | AGAGGCACTGGCAGAAAACA | 5 | |
| IL6_qPCR_R | TCACCAGGCAAGTCTCCTCA | 5 | |
| IL8_qPCR_F | CCAGGAAGAAACCACCGGAA | 5 | |
| IL8_qPCR_R | CTCCTTGGCAAAACTGCACC | 5 | |
| ATR_qPCR_F | TAGGGGAATTGGGGGCGATA | 6 | |
| ATR_qPCR_R | TGAATCTTCTACTCCAGTCACAAA | 6 | |
| GADD45A_qPCR_F | AGCAGAAGACCGAAAGGATGG | 6 | |
| GADD45A_qPCR_R | TGACTCAGGGCTTTGCTGAG | 6 | |
| GADD45B_qPCR_F | CTGGTCACGAACCCTCACAC | 6 | |
| GADD45B_qPCR_R | CTTTCTTCGCAGTAGCTGGC | 6 | |
| CCNB1_qPCR_F | GCCTGAGCCTATTTTGGTTGA | 6 | |
| CCNB1_qPCR_R | CTTCTTCTGCAGGGGCACAT | 6 | |
| TOP1_qPCR_F | GTCTGCGTCTCCCCCAC | 6 | |
| TOP1_qPCR_R | CGCTTCGATCTGGGAATCGT | 6 | |
| MRE11_qPCR_F | AGTCCAGCAGTGGGAATTTCT | 6 | |
| MRE11_qPCR_R | TCAGTCAAGCTCCTCTGGGA | 6 | |
| RAD9A_qPCR_F | GTGAAGGTGCTCGGCAAGG | 6 | |
| RAD9A_qPCR_R | CCAAGGGTTCCAGGTAGAGC | 6 | |
| RAD17_qPCR_F | GCGTTGAGCCCGGGTAG | 6 | |
| RAD17_qPCR_R | TGAATTCACTTGTTTCCCGGAG | 6 | |
| TOPBP1_qPCR_F | CCAGAAGCAAAGACTATGCCC | 6 | |
| TOPBP1_qPCR_R | TGAAAGAGTACGACTATCAATTGTG | 6 | |
| PCNA_qPCR_F | GCTCTTCCCTTACGCAAGTCT | 6 | |
| PCNA_qPCR_R | AGTCTAGCTGGTTTCGGCTT | 6 | |
| DDIT3_qPCR_F | AGGCACTGAGCGTATCATGT | 6 | |
| DDIT3_qPCR_R | CTTGAACACTCTCTCCTCAGGT | 6 | |
| DDIT4_qPCR_F | CCGGAGGAAGACACGGCTTA | 6 | |
| DDIT4_qPCR_R | GCATCAGGTTGGCACACAAG | 6 | |

**Supplementary Table S3** – Description of the antibodies used.

| **Antibody** | **Dilution** | **Manufacturer** |
| --- | --- | --- |
| p21 | 1/500 | BD Pharmingen, San Jose, CA |
| p27 | 1/500 | BD Transduction Laboratories, San Jose, CA |
| CD44 | 1/750 | Biocare Medical, Concord, CA |
| Lamin B1 | 1/1000 | Cell Signaling Technology, Danvers, MA |
| H3K9me3 | 1/1000 | EMD Millipore, Temecula, CA |
| H3 | 1/3000 | Cell Signaling Technology, Danvers, MA |
| Actin | 1/8000 | Sigma-Aldrich, CO., St. Louis, MO |

**Supplementary Table S4** – Gene Ontology terms for the altered miRNA.

| GO.ID | Term | Annotated | Significant | Expected | KS |
| --- | --- | --- | --- | --- | --- |
| GO:0060999 | positive regulation of dendritic spine d... | 21 | 21 | 21 | 0.00010 |
| GO:0006584 | catecholamine metabolic process | 34 | 34 | 34 | 0.00010 |
| GO:0009712 | catechol-containing compound metabolic p... | 34 | 34 | 34 | 0.00010 |
| GO:0048313 | Golgi inheritance | 11 | 11 | 11 | 0.00010 |
| GO:0072384 | organelle transport along microtubule | 34 | 34 | 34 | 0.00010 |
| GO:0033238 | regulation of cellular amine metabolic p... | 52 | 52 | 52 | 0.00010 |
| GO:0034694 | response to prostaglandin | 19 | 19 | 19 | 0.00010 |
| GO:0060693 | regulation of branching involved in sali... | 9 | 9 | 9 | 0.00010 |
| GO:0030811 | regulation of nucleotide catabolic proce... | 28 | 28 | 28 | 0.00010 |
| GO:1900181 | negative regulation of protein localizat... | 51 | 51 | 51 | 0.00010 |
| GO:0048485 | sympathetic nervous system development | 20 | 20 | 20 | 0.00011 |
| GO:1903513 | endoplasmic reticulum to cytosol transpo... | 40 | 40 | 40 | 0.00011 |
| GO:0000819 | sister chromatid segregation | 86 | 86 | 86 | 0.00011 |
| GO:0032106 | positive regulation of response to extra... | 28 | 28 | 28 | 0.00011 |
| GO:0032109 | positive regulation of response to nutri... | 28 | 28 | 28 | 0.00011 |
| GO:2000779 | regulation of double-strand break repair | 20 | 20 | 20 | 0.00011 |
| GO:0051439 | regulation of ubiquitin-protein ligase a... | 57 | 57 | 57 | 0.00011 |
| GO:0030166 | proteoglycan biosynthetic process | 48 | 48 | 48 | 0.00011 |
| GO:0048538 | thymus development | 31 | 31 | 31 | 0.00011 |
| GO:0060216 | definitive hemopoiesis | 19 | 19 | 19 | 0.00011 |
| GO:0035194 | posttranscriptional gene silencing by RN... | 36 | 36 | 36 | 0.00011 |
| GO:0002822 | regulation of adaptive immune response b... | 53 | 53 | 53 | 0.00011 |
| GO:0050777 | negative regulation of immune response | 53 | 53 | 53 | 0.00011 |
| GO:0006110 | regulation of glycolytic process | 24 | 24 | 24 | 0.00011 |
| GO:0045879 | negative regulation of smoothened signal... | 22 | 22 | 22 | 0.00012 |
| GO:0055088 | lipid homeostasis | 83 | 83 | 83 | 0.00012 |
| GO:1902803 | regulation of synaptic vesicle transport | 22 | 22 | 22 | 0.00012 |
| GO:0030104 | water homeostasis | 55 | 55 | 55 | 0.00012 |
| GO:0002700 | regulation of production of molecular me... | 50 | 50 | 50 | 0.00012 |
| GO:0051602 | response to electrical stimulus | 28 | 28 | 28 | 0.00012 |
| GO:0033273 | response to vitamin | 64 | 64 | 64 | 0.00012 |
| GO:0035329 | hippo signaling | 30 | 30 | 30 | 0.00012 |
| GO:0060736 | prostate gland growth | 9 | 9 | 9 | 0.00012 |
| GO:0033057 | multicellular organismal reproductive be... | 14 | 14 | 14 | 0.00012 |
| GO:2000766 | negative regulation of cytoplasmic trans... | 6 | 6 | 6 | 0.00012 |
| GO:0046889 | positive regulation of lipid biosyntheti... | 33 | 33 | 33 | 0.00012 |
| GO:0007189 | adenylate cyclase-activating G-protein c... | 49 | 49 | 49 | 0.00012 |
| GO:0045453 | bone resorption | 39 | 39 | 39 | 0.00012 |
| GO:0017156 | calcium ion-dependent exocytosis | 53 | 53 | 53 | 0.00012 |
| GO:0014741 | negative regulation of muscle hypertroph... | 12 | 12 | 12 | 0.00012 |
| GO:0007368 | determination of left/right symmetry | 65 | 65 | 65 | 0.00012 |
| GO:0019083 | viral transcription | 88 | 88 | 88 | 0.00013 |
| GO:0010092 | specification of organ identity | 24 | 24 | 24 | 0.00013 |
| GO:0031112 | positive regulation of microtubule polym... | 16 | 16 | 16 | 0.00013 |
| GO:0061462 | protein localization to lysosome | 16 | 16 | 16 | 0.00013 |
| GO:0003151 | outflow tract morphogenesis | 44 | 44 | 44 | 0.00013 |
| GO:0006354 | elongation | 78 | 78 | 78 | 0.00013 |
| GO:0006090 | pyruvate metabolic process | 70 | 70 | 70 | 0.00013 |
| GO:1901385 | regulation of voltage-gated calcium chan... | 19 | 19 | 19 | 0.00013 |
| GO:0003156 | regulation of organ formation | 26 | 26 | 26 | 0.00013 |
| GO:2000142 | regulation of DNA-templated transcriptio... | 23 | 23 | 23 | 0.00013 |
| GO:1903051 | negative regulation of proteolysis invol... | 50 | 50 | 50 | 0.00013 |
| GO:0032091 | negative regulation of protein binding | 48 | 48 | 48 | 0.00013 |
| GO:0071236 | cellular response to antibiotic | 11 | 11 | 11 | 0.00013 |
| GO:0021670 | lateral ventricle development | 10 | 10 | 10 | 0.00013 |
| GO:0040034 | regulation of development heterochronic | 12 | 12 | 12 | 0.00013 |
| GO:0071479 | cellular response to ionizing radiation | 36 | 36 | 36 | 0.00013 |
| GO:0021889 | olfactory bulb interneuron differentiati... | 10 | 10 | 10 | 0.00013 |
| GO:0060026 | convergent extension | 10 | 10 | 10 | 0.00013 |
| GO:0006509 | membrane protein ectodomain proteolysis | 26 | 26 | 26 | 0.00013 |
| GO:0045684 | positive regulation of epidermis develop... | 18 | 18 | 18 | 0.00014 |
| GO:0031936 | negative regulation of chromatin silenci... | 8 | 8 | 8 | 0.00014 |
| GO:0086009 | membrane repolarization | 29 | 29 | 29 | 0.00014 |
| GO:0006901 | vesicle coating | 32 | 32 | 32 | 0.00014 |
| GO:0070570 | regulation of neuron projection regenera... | 15 | 15 | 15 | 0.00014 |
| GO:0035929 | steroid hormone secretion | 13 | 13 | 13 | 0.00014 |
| GO:0000183 | chromatin silencing at rDNA | 23 | 23 | 23 | 0.00014 |
| GO:0060438 | trachea development | 16 | 16 | 16 | 0.00014 |
| GO:0000245 | spliceosomal complex assembly | 37 | 37 | 37 | 0.00014 |
| GO:0001754 | eye photoreceptor cell differentiation | 30 | 30 | 30 | 0.00014 |
| GO:0060119 | inner ear receptor cell development | 26 | 26 | 26 | 0.00014 |
| GO:0097484 | dendrite extension | 16 | 16 | 16 | 0.00014 |
| GO:0044705 | multi-organism reproductive behavior | 19 | 19 | 19 | 0.00014 |
| GO:1903206 | negative regulation of hydrogen peroxide... | 6 | 6 | 6 | 0.00014 |
| GO:0006903 | vesicle targeting | 35 | 35 | 35 | 0.00015 |
| GO:0051193 | regulation of cofactor metabolic process | 38 | 38 | 38 | 0.00015 |
| GO:0051196 | regulation of coenzyme metabolic process | 38 | 38 | 38 | 0.00015 |
| GO:0036503 | ERAD pathway | 59 | 59 | 59 | 0.00015 |
| GO:0071634 | regulation of transforming growth factor... | 18 | 18 | 18 | 0.00015 |
| GO:2000179 | positive regulation of neural precursor ... | 31 | 31 | 31 | 0.00015 |
| GO:0050728 | negative regulation of inflammatory resp... | 50 | 50 | 50 | 0.00015 |
| GO:0010801 | negative regulation of peptidyl-threonin... | 13 | 13 | 13 | 0.00015 |
| GO:0032011 | ARF protein signal transduction | 15 | 15 | 15 | 0.00015 |
| GO:0032012 | regulation of ARF protein signal transdu... | 15 | 15 | 15 | 0.00015 |
| GO:0051437 | positive regulation of ubiquitin-protein... | 38 | 38 | 38 | 0.00016 |
| GO:0019886 | antigen processing and presentation of e... | 55 | 55 | 55 | 0.00016 |
| GO:0007040 | lysosome organization | 34 | 34 | 34 | 0.00016 |
| GO:0080171 | lytic vacuole organization | 34 | 34 | 34 | 0.00016 |
| GO:0045197 | establishment or maintenance of epitheli... | 22 | 22 | 22 | 0.00016 |
| GO:0090311 | regulation of protein deacetylation | 24 | 24 | 24 | 0.00016 |
| GO:0043090 | amino acid import | 14 | 14 | 14 | 0.00016 |
| GO:0033962 | cytoplasmic mRNA processing body assembl... | 14 | 14 | 14 | 0.00016 |
| GO:0006757 | ATP generation from ADP | 48 | 48 | 48 | 0.00016 |
| GO:0072329 | monocarboxylic acid catabolic process | 66 | 66 | 66 | 0.00016 |
| GO:0021681 | cerebellar granular layer development | 12 | 12 | 12 | 0.00016 |
| GO:0031641 | regulation of myelination | 25 | 25 | 25 | 0.00016 |
| GO:0042304 | regulation of fatty acid biosynthetic pr... | 22 | 22 | 22 | 0.00016 |
| GO:0014887 | cardiac muscle adaptation | 16 | 16 | 16 | 0.00016 |
| GO:2000273 | positive regulation of receptor activity | 23 | 23 | 23 | 0.00017 |
| GO:0045599 | negative regulation of fat cell differen... | 31 | 31 | 31 | 0.00017 |
| GO:0044091 | membrane biogenesis | 27 | 27 | 27 | 0.00017 |
| GO:0033077 | T cell differentiation in thymus | 55 | 55 | 55 | 0.00017 |
| GO:0071594 | thymocyte aggregation | 55 | 55 | 55 | 0.00017 |
| GO:0035850 | epithelial cell differentiation involved... | 24 | 24 | 24 | 0.00017 |
| GO:0035411 | catenin import into nucleus | 22 | 22 | 22 | 0.00017 |
| GO:0035412 | regulation of catenin import into nucleu... | 22 | 22 | 22 | 0.00017 |
| GO:0046631 | alpha-beta T cell activation | 68 | 68 | 68 | 0.00017 |
| GO:0031062 | positive regulation of histone methylati... | 27 | 27 | 27 | 0.00017 |
| GO:0080182 | histone H3-K4 trimethylation | 10 | 10 | 10 | 0.00017 |
| GO:0050732 | negative regulation of peptidyl-tyrosine... | 26 | 26 | 26 | 0.00018 |
| GO:0044783 | G1 DNA damage checkpoint | 47 | 47 | 47 | 0.00018 |
| GO:0003197 | endocardial cushion development | 22 | 22 | 22 | 0.00018 |
| GO:0007016 | cytoskeletal anchoring at plasma membran... | 11 | 11 | 11 | 0.00018 |
| GO:0006833 | water transport | 32 | 32 | 32 | 0.00018 |
| GO:2000463 | positive regulation of excitatory postsy... | 15 | 15 | 15 | 0.00018 |
| GO:0046887 | positive regulation of hormone secretion | 69 | 69 | 69 | 0.00018 |
| GO:0036474 | cell death in response to hydrogen perox... | 8 | 8 | 8 | 0.00018 |
| GO:0010389 | regulation of G2/M transition of mitotic... | 38 | 38 | 38 | 0.00018 |
| GO:1903727 | positive regulation of phospholipid meta... | 30 | 30 | 30 | 0.00018 |
| GO:0045912 | negative regulation of carbohydrate meta... | 31 | 31 | 31 | 0.00018 |
| GO:0010677 | negative regulation of cellular carbohyd... | 27 | 27 | 27 | 0.00019 |
| GO:0006513 | protein monoubiquitination | 43 | 43 | 43 | 0.00019 |
| GO:0045580 | regulation of T cell differentiation | 70 | 70 | 70 | 0.00019 |
| GO:0001953 | negative regulation of cell-matrix adhes... | 24 | 24 | 24 | 0.00019 |
| GO:0043392 | negative regulation of DNA binding | 35 | 35 | 35 | 0.00019 |
| GO:0010881 | regulation of cardiac muscle contraction... | 15 | 15 | 15 | 0.00019 |
| GO:0048025 | negative regulation of mRNA splicing | 18 | 18 | 18 | 0.00020 |
| GO:0051225 | spindle assembly | 55 | 55 | 55 | 0.00020 |
| GO:0006939 | smooth muscle contraction | 64 | 64 | 64 | 0.00020 |
| GO:0016239 | positive regulation of macroautophagy | 27 | 27 | 27 | 0.00020 |
| GO:0021532 | neural tube patterning | 32 | 32 | 32 | 0.00020 |
| GO:0030514 | negative regulation of BMP signaling pat... | 33 | 33 | 33 | 0.00020 |
| GO:0061029 | eyelid development in camera-type eye | 10 | 10 | 10 | 0.00020 |
| GO:0090342 | regulation of cell aging | 26 | 26 | 26 | 0.00020 |
| GO:2000505 | regulation of energy homeostasis | 10 | 10 | 10 | 0.00020 |
| GO:0051893 | regulation of focal adhesion assembly | 38 | 38 | 38 | 0.00020 |
| GO:0090109 | regulation of cell-substrate junction as... | 38 | 38 | 38 | 0.00020 |
| GO:0046513 | ceramide biosynthetic process | 35 | 35 | 35 | 0.00020 |
| GO:1901136 | carbohydrate derivative catabolic proces... | 110 | 110 | 110 | 0.00020 |
| GO:1990090 | cellular response to nerve growth factor... | 20 | 20 | 20 | 0.00020 |
| GO:0001659 | temperature homeostasis | 24 | 24 | 24 | 0.00021 |
| GO:0035195 | gene silencing by miRNA | 33 | 33 | 33 | 0.00021 |
| GO:0006891 | intra-Golgi vesicle-mediated transport | 31 | 31 | 31 | 0.00021 |
| GO:0036465 | synaptic vesicle recycling | 26 | 26 | 26 | 0.00021 |
| GO:2001021 | negative regulation of response to DNA d... | 36 | 36 | 36 | 0.00021 |
| GO:0009950 | dorsal/ventral axis specification | 18 | 18 | 18 | 0.00021 |
| GO:0007379 | segment specification | 13 | 13 | 13 | 0.00021 |
| GO:0097104 | postsynaptic membrane assembly | 10 | 10 | 10 | 0.00021 |
| GO:0050434 | positive regulation of viral transcripti... | 38 | 38 | 38 | 0.00021 |
| GO:0009583 | detection of light stimulus | 74 | 74 | 74 | 0.00022 |
| GO:0070296 | sarcoplasmic reticulum calcium ion trans... | 21 | 21 | 21 | 0.00022 |
| GO:1902742 | apoptotic process involved in developmen... | 27 | 27 | 27 | 0.00022 |
| GO:0045661 | regulation of myoblast differentiation | 34 | 34 | 34 | 0.00022 |
| GO:0008333 | endosome to lysosome transport | 32 | 32 | 32 | 0.00022 |
| GO:0002526 | acute inflammatory response | 70 | 70 | 70 | 0.00022 |
| GO:0016064 | immunoglobulin mediated immune response | 52 | 52 | 52 | 0.00022 |
| GO:0030216 | keratinocyte differentiation | 68 | 68 | 68 | 0.00022 |
| GO:0048008 | platelet-derived growth factor receptor ... | 36 | 36 | 36 | 0.00022 |
| GO:0035150 | regulation of tube size | 90 | 90 | 90 | 0.00022 |
| GO:0051445 | regulation of meiotic cell cycle | 22 | 22 | 22 | 0.00022 |
| GO:0016574 | histone ubiquitination | 30 | 30 | 30 | 0.00023 |
| GO:0071320 | cellular response to cAMP | 38 | 38 | 38 | 0.00023 |
| GO:0008217 | regulation of blood pressure | 108 | 108 | 108 | 0.00023 |
| GO:0060439 | trachea morphogenesis | 8 | 8 | 8 | 0.00023 |
| GO:0050891 | multicellular organismal water homeostas... | 46 | 46 | 46 | 0.00023 |
| GO:0006413 | translational initiation | 109 | 109 | 109 | 0.00023 |
| GO:1901186 | positive regulation of ERBB signaling pa... | 15 | 15 | 15 | 0.00023 |
| GO:0014904 | myotube cell development | 22 | 22 | 22 | 0.00023 |
| GO:0046530 | photoreceptor cell differentiation | 37 | 37 | 37 | 0.00023 |
| GO:0034405 | response to fluid shear stress | 29 | 29 | 29 | 0.00024 |
| GO:1990089 | response to nerve growth factor | 23 | 23 | 23 | 0.00024 |
| GO:1903078 | positive regulation of protein localizat... | 25 | 25 | 25 | 0.00024 |
| GO:1904377 | positive regulation of protein localizat... | 25 | 25 | 25 | 0.00024 |
| GO:0006883 | cellular sodium ion homeostasis | 13 | 13 | 13 | 0.00024 |
| GO:0002320 | lymphoid progenitor cell differentiation | 12 | 12 | 12 | 0.00024 |
| GO:0031115 | negative regulation of microtubule polym... | 7 | 7 | 7 | 0.00024 |
| GO:0009108 | coenzyme biosynthetic process | 71 | 71 | 71 | 0.00024 |
| GO:0050715 | positive regulation of cytokine secretio... | 38 | 38 | 38 | 0.00024 |
| GO:0032946 | positive regulation of mononuclear cell ... | 66 | 66 | 66 | 0.00024 |
| GO:0045324 | late endosome to vacuole transport | 11 | 11 | 11 | 0.00024 |
| GO:0001783 | B cell apoptotic process | 17 | 17 | 17 | 0.00024 |
| GO:0070932 | histone H3 deacetylation | 20 | 20 | 20 | 0.00024 |
| GO:0010172 | embryonic body morphogenesis | 13 | 13 | 13 | 0.00024 |
| GO:0042157 | lipoprotein metabolic process | 86 | 86 | 86 | 0.00025 |
| GO:0035883 | enteroendocrine cell differentiation | 25 | 25 | 25 | 0.00025 |
| GO:2000737 | negative regulation of stem cell differe... | 36 | 36 | 36 | 0.00025 |
| GO:0030032 | lamellipodium assembly | 48 | 48 | 48 | 0.00025 |
| GO:0048525 | negative regulation of viral process | 48 | 48 | 48 | 0.00025 |
| GO:0002712 | regulation of B cell mediated immunity | 25 | 25 | 25 | 0.00025 |
| GO:0002474 | antigen processing and presentation of p... | 57 | 57 | 57 | 0.00025 |
| GO:0035826 | rubidium ion transport | 5 | 5 | 5 | 0.00026 |
| GO:1903077 | negative regulation of protein localizat... | 18 | 18 | 18 | 0.00026 |
| GO:1904376 | negative regulation of protein localizat... | 18 | 18 | 18 | 0.00026 |
| GO:0071709 | membrane assembly | 24 | 24 | 24 | 0.00026 |
| GO:1902235 | regulation of endoplasmic reticulum stre... | 26 | 26 | 26 | 0.00026 |
| GO:0007601 | visual perception | 112 | 112 | 112 | 0.00026 |
| GO:0060324 | face development | 38 | 38 | 38 | 0.00026 |
| GO:0051491 | positive regulation of filopodium assemb... | 18 | 18 | 18 | 0.00026 |
| GO:0051928 | positive regulation of calcium ion trans... | 65 | 65 | 65 | 0.00026 |
| GO:0051436 | negative regulation of ubiquitin-protein... | 49 | 49 | 49 | 0.00026 |
| GO:0055076 | transition metal ion homeostasis | 91 | 91 | 91 | 0.00026 |
| GO:0060674 | placenta blood vessel development | 20 | 20 | 20 | 0.00026 |
| GO:0030325 | adrenal gland development | 23 | 23 | 23 | 0.00026 |
| GO:0006094 | gluconeogenesis | 54 | 54 | 54 | 0.00026 |
| GO:1903523 | negative regulation of blood circulation | 26 | 26 | 26 | 0.00026 |
| GO:0014823 | response to activity | 35 | 35 | 35 | 0.00027 |
| GO:1903901 | negative regulation of viral life cycle | 46 | 46 | 46 | 0.00027 |
| GO:2000209 | regulation of anoikis | 18 | 18 | 18 | 0.00027 |
| GO:0070509 | calcium ion import | 38 | 38 | 38 | 0.00027 |
| GO:2000108 | positive regulation of leukocyte apoptot... | 14 | 14 | 14 | 0.00027 |
| GO:2000345 | regulation of hepatocyte proliferation | 9 | 9 | 9 | 0.00027 |
| GO:0033555 | multicellular organismal response to str... | 59 | 59 | 59 | 0.00027 |
| GO:0051496 | positive regulation of stress fiber asse... | 34 | 34 | 34 | 0.00027 |
| GO:0070670 | response to interleukin-4 | 22 | 22 | 22 | 0.00027 |
| GO:0090199 | regulation of release of cytochrome c fr... | 26 | 26 | 26 | 0.00027 |
| GO:0090398 | cellular senescence | 43 | 43 | 43 | 0.00028 |
| GO:0051865 | protein autoubiquitination | 43 | 43 | 43 | 0.00028 |
| GO:0032515 | negative regulation of phosphoprotein ph... | 11 | 11 | 11 | 0.00028 |
| GO:0044819 | mitotic G1/S transition checkpoint | 47 | 47 | 47 | 0.00028 |
| GO:0045762 | positive regulation of adenylate cyclase... | 37 | 37 | 37 | 0.00028 |
| GO:0016024 | CDP-diacylglycerol biosynthetic process | 11 | 11 | 11 | 0.00029 |
| GO:0046341 | CDP-diacylglycerol metabolic process | 11 | 11 | 11 | 0.00029 |
| GO:0055119 | relaxation of cardiac muscle | 14 | 14 | 14 | 0.00029 |
| GO:0071392 | cellular response to estradiol stimulus | 16 | 16 | 16 | 0.00029 |
| GO:0010800 | positive regulation of peptidyl-threonin... | 20 | 20 | 20 | 0.00029 |
| GO:0036445 | neuronal stem cell division | 9 | 9 | 9 | 0.00029 |
| GO:0055057 | neuroblast division | 9 | 9 | 9 | 0.00029 |
| GO:0090004 | positive regulation of establishment of ... | 23 | 23 | 23 | 0.00029 |
| GO:0051983 | regulation of chromosome segregation | 54 | 54 | 54 | 0.00029 |
| GO:0040036 | regulation of fibroblast growth factor r... | 20 | 20 | 20 | 0.00030 |
| GO:0014003 | oligodendrocyte development | 24 | 24 | 24 | 0.00030 |
| GO:0002718 | regulation of cytokine production involv... | 27 | 27 | 27 | 0.00030 |
| GO:0043092 | L-amino acid import | 13 | 13 | 13 | 0.00030 |
| GO:0010811 | positive regulation of cell-substrate ad... | 76 | 76 | 76 | 0.00030 |
| GO:0036037 | CD8-positive  alpha-beta T cell activati... | 8 | 8 | 8 | 0.00030 |
| GO:0045579 | positive regulation of B cell differenti... | 12 | 12 | 12 | 0.00030 |
| GO:0036303 | lymph vessel morphogenesis | 14 | 14 | 14 | 0.00030 |
| GO:0003309 | type B pancreatic cell differentiation | 23 | 23 | 23 | 0.00030 |
| GO:0030520 | intracellular estrogen receptor signalin... | 33 | 33 | 33 | 0.00030 |
| GO:0002902 | regulation of B cell apoptotic process | 13 | 13 | 13 | 0.00030 |
| GO:0019054 | modulation by virus of host process | 11 | 11 | 11 | 0.00030 |
| GO:0009948 | anterior/posterior axis specification | 39 | 39 | 39 | 0.00030 |
| GO:0070555 | response to interleukin-1 | 74 | 74 | 74 | 0.00031 |
| GO:0031572 | G2 DNA damage checkpoint | 27 | 27 | 27 | 0.00031 |
| GO:0044068 | modulation by symbiont of host cellular ... | 13 | 13 | 13 | 0.00031 |
| GO:0042308 | negative regulation of protein import in... | 49 | 49 | 49 | 0.00031 |
| GO:1904590 | negative regulation of protein import | 49 | 49 | 49 | 0.00031 |
| GO:0046165 | alcohol biosynthetic process | 99 | 99 | 99 | 0.00031 |
| GO:0019395 | fatty acid oxidation | 62 | 62 | 62 | 0.00031 |
| GO:0032469 | endoplasmic reticulum calcium ion homeos... | 18 | 18 | 18 | 0.00031 |
| GO:0034199 | activation of protein kinase A activity | 14 | 14 | 14 | 0.00031 |
| GO:0003211 | cardiac ventricle formation | 8 | 8 | 8 | 0.00031 |
| GO:0072160 | nephron tubule epithelial cell different... | 13 | 13 | 13 | 0.00032 |
| GO:0060969 | negative regulation of gene silencing | 12 | 12 | 12 | 0.00032 |
| GO:0034104 | negative regulation of tissue remodeling | 10 | 10 | 10 | 0.00032 |
| GO:0060384 | innervation | 20 | 20 | 20 | 0.00032 |
| GO:0032459 | regulation of protein oligomerization | 24 | 24 | 24 | 0.00032 |
| GO:0050994 | regulation of lipid catabolic process | 28 | 28 | 28 | 0.00032 |
| GO:0007602 | phototransduction | 65 | 65 | 65 | 0.00032 |
| GO:0002263 | cell activation involved in immune respo... | 119 | 119 | 119 | 0.00032 |
| GO:0050880 | regulation of blood vessel size | 89 | 89 | 89 | 0.00032 |
| GO:0034770 | histone H4-K20 methylation | 9 | 9 | 9 | 0.00032 |
| GO:0018198 | peptidyl-cysteine modification | 29 | 29 | 29 | 0.00032 |
| GO:0010226 | response to lithium ion | 19 | 19 | 19 | 0.00032 |
| GO:0032414 | positive regulation of ion transmembrane... | 43 | 43 | 43 | 0.00033 |
| GO:0045737 | positive regulation of cyclin-dependent ... | 21 | 21 | 21 | 0.00033 |
| GO:0031650 | regulation of heat generation | 9 | 9 | 9 | 0.00033 |
| GO:0070228 | regulation of lymphocyte apoptotic proce... | 30 | 30 | 30 | 0.00033 |
| GO:0031290 | retinal ganglion cell axon guidance | 17 | 17 | 17 | 0.00033 |
| GO:0007520 | myoblast fusion | 28 | 28 | 28 | 0.00033 |
| GO:0048384 | retinoic acid receptor signaling pathway | 25 | 25 | 25 | 0.00033 |
| GO:0060039 | pericardium development | 15 | 15 | 15 | 0.00033 |
| GO:0060174 | limb bud formation | 8 | 8 | 8 | 0.00034 |
| GO:0070665 | positive regulation of leukocyte prolife... | 67 | 67 | 67 | 0.00034 |
| GO:0051898 | negative regulation of protein kinase B ... | 24 | 24 | 24 | 0.00034 |
| GO:0051341 | regulation of oxidoreductase activity | 55 | 55 | 55 | 0.00034 |
| GO:0097237 | cellular response to toxic substance | 14 | 14 | 14 | 0.00034 |
| GO:0048712 | negative regulation of astrocyte differe... | 9 | 9 | 9 | 0.00035 |
| GO:0031023 | microtubule organizing center organizati... | 73 | 73 | 73 | 0.00035 |
| GO:0000070 | mitotic sister chromatid segregation | 80 | 80 | 80 | 0.00035 |
| GO:0051220 | cytoplasmic sequestering of protein | 29 | 29 | 29 | 0.00035 |
| GO:0000018 | regulation of DNA recombination | 34 | 34 | 34 | 0.00035 |
| GO:0061005 | cell differentiation involved in kidney ... | 32 | 32 | 32 | 0.00035 |
| GO:0050879 | multicellular organismal movement | 27 | 27 | 27 | 0.00035 |
| GO:0050881 | musculoskeletal movement | 27 | 27 | 27 | 0.00035 |
| GO:0030219 | megakaryocyte differentiation | 29 | 29 | 29 | 0.00035 |
| GO:0060638 | mesenchymal-epithelial cell signaling | 8 | 8 | 8 | 0.00035 |
| GO:0006368 | transcription elongation from RNA polyme... | 58 | 58 | 58 | 0.00035 |
| GO:0006688 | glycosphingolipid biosynthetic process | 23 | 23 | 23 | 0.00035 |
| GO:0006353 | DNA-templated transcription  termination | 54 | 54 | 54 | 0.00036 |
| GO:0043403 | skeletal muscle tissue regeneration | 24 | 24 | 24 | 0.00036 |
| GO:0042990 | regulation of transcription factor impor... | 65 | 65 | 65 | 0.00036 |
| GO:0006888 | ER to Golgi vesicle-mediated transport | 63 | 63 | 63 | 0.00036 |
| GO:0060749 | mammary gland alveolus development | 14 | 14 | 14 | 0.00036 |
| GO:0061377 | mammary gland lobule development | 14 | 14 | 14 | 0.00036 |
| GO:0031571 | mitotic G1 DNA damage checkpoint | 46 | 46 | 46 | 0.00036 |
| GO:0018345 | protein palmitoylation | 20 | 20 | 20 | 0.00037 |
| GO:0042634 | regulation of hair cycle | 16 | 16 | 16 | 0.00037 |
| GO:0006376 | mRNA splice site selection | 23 | 23 | 23 | 0.00037 |
| GO:0031103 | axon regeneration | 27 | 27 | 27 | 0.00037 |
| GO:0045581 | negative regulation of T cell differenti... | 19 | 19 | 19 | 0.00038 |
| GO:1900117 | regulation of execution phase of apoptos... | 18 | 18 | 18 | 0.00038 |
| GO:0045071 | negative regulation of viral genome repl... | 20 | 20 | 20 | 0.00038 |
| GO:0006953 | acute-phase response | 25 | 25 | 25 | 0.00038 |
| GO:0043276 | anoikis | 22 | 22 | 22 | 0.00038 |
| GO:0002070 | epithelial cell maturation | 14 | 14 | 14 | 0.00038 |
| GO:1903670 | regulation of sprouting angiogenesis | 14 | 14 | 14 | 0.00038 |
| GO:0006195 | purine nucleotide catabolic process | 30 | 30 | 30 | 0.00038 |
| GO:0042147 | retrograde transport endosome to Golgi | 54 | 54 | 54 | 0.00038 |
| GO:0031644 | regulation of neurological system proces... | 47 | 47 | 47 | 0.00038 |
| GO:0060351 | cartilage development involved in endoch... | 21 | 21 | 21 | 0.00038 |
| GO:0019319 | hexose biosynthetic process | 56 | 56 | 56 | 0.00038 |
| GO:0021800 | cerebral cortex tangential migration | 9 | 9 | 9 | 0.00039 |
| GO:0019098 | reproductive behavior | 20 | 20 | 20 | 0.00039 |
| GO:0070076 | histone lysine demethylation | 21 | 21 | 21 | 0.00039 |
| GO:0060059 | embryonic retina morphogenesis in camera... | 6 | 6 | 6 | 0.00039 |
| GO:0045992 | negative regulation of embryonic develop... | 22 | 22 | 22 | 0.00039 |
| GO:2000191 | regulation of fatty acid transport | 12 | 12 | 12 | 0.00039 |
| GO:0001885 | endothelial cell development | 42 | 42 | 42 | 0.00039 |
| GO:0060850 | regulation of transcription involved in ... | 17 | 17 | 17 | 0.00039 |
| GO:0051148 | negative regulation of muscle cell diffe... | 42 | 42 | 42 | 0.00039 |
| GO:0070544 | histone H3-K36 demethylation | 5 | 5 | 5 | 0.00040 |
| GO:0098815 | modulation of excitatory postsynaptic po... | 21 | 21 | 21 | 0.00040 |
| GO:0072132 | mesenchyme morphogenesis | 28 | 28 | 28 | 0.00041 |
| GO:0051150 | regulation of smooth muscle cell differe... | 10 | 10 | 10 | 0.00041 |
| GO:0006356 | regulation of transcription from RNA pol... | 15 | 15 | 15 | 0.00042 |
| GO:0048505 | regulation of timing of cell differentia... | 11 | 11 | 11 | 0.00042 |
| GO:0071715 | icosanoid transport | 16 | 16 | 16 | 0.00042 |
| GO:1901571 | fatty acid derivative transport | 16 | 16 | 16 | 0.00042 |
| GO:0009214 | cyclic nucleotide catabolic process | 19 | 19 | 19 | 0.00043 |
| GO:0061549 | sympathetic ganglion development | 9 | 9 | 9 | 0.00043 |
| GO:0038092 | nodal signaling pathway | 9 | 9 | 9 | 0.00043 |
| GO:0043647 | inositol phosphate metabolic process | 51 | 51 | 51 | 0.00043 |
| GO:0021683 | cerebellar granular layer morphogenesis | 9 | 9 | 9 | 0.00044 |
| GO:0051653 | spindle localization | 29 | 29 | 29 | 0.00044 |
| GO:0034505 | tooth mineralization | 13 | 13 | 13 | 0.00045 |
| GO:0048096 | chromatin-mediated maintenance of transc... | 8 | 8 | 8 | 0.00045 |
| GO:0060669 | embryonic placenta morphogenesis | 18 | 18 | 18 | 0.00045 |
| GO:0032800 | receptor biosynthetic process | 22 | 22 | 22 | 0.00045 |
| GO:0070373 | negative regulation of ERK1 and ERK2 cas... | 39 | 39 | 39 | 0.00045 |
| GO:0060841 | venous blood vessel development | 14 | 14 | 14 | 0.00045 |
| GO:0008542 | visual learning | 35 | 35 | 35 | 0.00045 |
| GO:0048742 | regulation of skeletal muscle fiber deve... | 7 | 7 | 7 | 0.00045 |
| GO:0002366 | leukocyte activation involved in immune ... | 117 | 117 | 117 | 0.00045 |
| GO:1900034 | regulation of cellular response to heat | 53 | 53 | 53 | 0.00046 |
| GO:0032233 | positive regulation of actin filament bu... | 38 | 38 | 38 | 0.00046 |
| GO:0043388 | positive regulation of DNA binding | 30 | 30 | 30 | 0.00046 |
| GO:0045022 | early endosome to late endosome transpor... | 30 | 30 | 30 | 0.00046 |
| GO:0048016 | inositol phosphate-mediated signaling | 28 | 28 | 28 | 0.00046 |
| GO:0016322 | neuron remodeling | 8 | 8 | 8 | 0.00046 |
| GO:1902307 | positive regulation of sodium ion transm... | 14 | 14 | 14 | 0.00046 |
| GO:0050671 | positive regulation of lymphocyte prolif... | 65 | 65 | 65 | 0.00046 |
| GO:0000381 | regulation of alternative mRNA splicing | 25 | 25 | 25 | 0.00047 |
| GO:0060135 | maternal process involved in female preg... | 40 | 40 | 40 | 0.00047 |
| GO:2001057 | reactive nitrogen species metabolic proc... | 58 | 58 | 58 | 0.00047 |
| GO:0007528 | neuromuscular junction development | 34 | 34 | 34 | 0.00047 |
| GO:0072207 | metanephric epithelium development | 21 | 21 | 21 | 0.00047 |
| GO:0045471 | response to ethanol | 76 | 76 | 76 | 0.00048 |
| GO:0060484 | lung-associated mesenchyme development | 7 | 7 | 7 | 0.00048 |
| GO:0070613 | regulation of protein processing | 47 | 47 | 47 | 0.00049 |
| GO:0002495 | antigen processing and presentation of p... | 57 | 57 | 57 | 0.00049 |
| GO:0002504 | antigen processing and presentation of p... | 57 | 57 | 57 | 0.00049 |
| GO:0060525 | prostate glandular acinus development | 10 | 10 | 10 | 0.00049 |
| GO:0014033 | neural crest cell differentiation | 50 | 50 | 50 | 0.00049 |
| GO:0098813 | nuclear chromosome segregation | 109 | 109 | 109 | 0.00050 |
| GO:0045648 | positive regulation of erythrocyte diffe... | 18 | 18 | 18 | 0.00050 |
| GO:0010831 | positive regulation of myotube different... | 19 | 19 | 19 | 0.00050 |
| GO:0040023 | establishment of nucleus localization | 14 | 14 | 14 | 0.00050 |
| GO:0032069 | regulation of nuclease activity | 13 | 13 | 13 | 0.00051 |
| GO:0010002 | cardioblast differentiation | 18 | 18 | 18 | 0.00051 |
| GO:0043923 | positive regulation by host of viral tra... | 11 | 11 | 11 | 0.00052 |
| GO:0072673 | lamellipodium morphogenesis | 13 | 13 | 13 | 0.00052 |
| GO:0006520 | cellular amino acid metabolic process | 255 | 255 | 255 | 0.00052 |
| GO:0046825 | regulation of protein export from nucleu... | 21 | 21 | 21 | 0.00052 |
| GO:0090177 | establishment of planar polarity involve... | 11 | 11 | 11 | 0.00053 |
| GO:0048486 | parasympathetic nervous system developme... | 14 | 14 | 14 | 0.00053 |
| GO:0048665 | neuron fate specification | 25 | 25 | 25 | 0.00053 |
| GO:0045214 | sarcomere organization | 21 | 21 | 21 | 0.00053 |
| GO:0034440 | lipid oxidation | 63 | 63 | 63 | 0.00053 |
| GO:0051294 | establishment of spindle orientation | 20 | 20 | 20 | 0.00054 |
| GO:0072661 | protein targeting to plasma membrane | 18 | 18 | 18 | 0.00054 |
| GO:2000052 | positive regulation of non-canonical Wnt... | 7 | 7 | 7 | 0.00054 |
| GO:2000739 | regulation of mesenchymal stem cell diff... | 6 | 6 | 6 | 0.00054 |
| GO:0007632 | visual behavior | 40 | 40 | 40 | 0.00054 |
| GO:0010869 | regulation of receptor biosynthetic proc... | 18 | 18 | 18 | 0.00054 |
| GO:0006261 | DNA-dependent DNA replication | 69 | 69 | 69 | 0.00055 |
| GO:0046839 | phospholipid dephosphorylation | 23 | 23 | 23 | 0.00055 |
| GO:0060068 | vagina development | 8 | 8 | 8 | 0.00056 |
| GO:0010880 | regulation of release of sequestered cal... | 18 | 18 | 18 | 0.00056 |
| GO:0021517 | ventral spinal cord development | 38 | 38 | 38 | 0.00056 |
| GO:0042991 | transcription factor import into nucleus | 66 | 66 | 66 | 0.00056 |
| GO:0035871 | protein K11-linked deubiquitination | 7 | 7 | 7 | 0.00056 |
| GO:0021799 | cerebral cortex radially oriented cell m... | 24 | 24 | 24 | 0.00057 |
| GO:0097284 | hepatocyte apoptotic process | 13 | 13 | 13 | 0.00057 |
| GO:0045807 | positive regulation of endocytosis | 78 | 78 | 78 | 0.00057 |
| GO:0002699 | positive regulation of immune effector p... | 86 | 86 | 86 | 0.00057 |
| GO:0021983 | pituitary gland development | 28 | 28 | 28 | 0.00057 |
| GO:0045806 | negative regulation of endocytosis | 24 | 24 | 24 | 0.00058 |
| GO:0021604 | cranial nerve structural organization | 8 | 8 | 8 | 0.00058 |
| GO:0046823 | negative regulation of nucleocytoplasmic... | 52 | 52 | 52 | 0.00058 |
| GO:0071353 | cellular response to interleukin-4 | 18 | 18 | 18 | 0.00059 |
| GO:0048488 | synaptic vesicle endocytosis | 23 | 23 | 23 | 0.00059 |
| GO:0030433 | ER-associated ubiquitin-dependent protei... | 49 | 49 | 49 | 0.00059 |
| GO:0045739 | positive regulation of DNA repair | 20 | 20 | 20 | 0.00059 |
| GO:0046209 | nitric oxide metabolic process | 56 | 56 | 56 | 0.00059 |
| GO:0002889 | regulation of immunoglobulin mediated im... | 24 | 24 | 24 | 0.00059 |
| GO:0046916 | cellular transition metal ion homeostasi... | 68 | 68 | 68 | 0.00059 |
| GO:0002181 | cytoplasmic translation | 22 | 22 | 22 | 0.00060 |
| GO:0001706 | endoderm formation | 47 | 47 | 47 | 0.00060 |
| GO:0009261 | ribonucleotide catabolic process | 23 | 23 | 23 | 0.00061 |
| GO:0007190 | activation of adenylate cyclase activity | 27 | 27 | 27 | 0.00061 |
| GO:0060391 | positive regulation of SMAD protein impo... | 8 | 8 | 8 | 0.00061 |
| GO:0046856 | phosphatidylinositol dephosphorylation | 18 | 18 | 18 | 0.00061 |
| GO:0016054 | organic acid catabolic process | 133 | 133 | 133 | 0.00061 |
| GO:0046395 | carboxylic acid catabolic process | 133 | 133 | 133 | 0.00061 |
| GO:0033138 | positive regulation of peptidyl-serine p... | 60 | 60 | 60 | 0.00061 |
| GO:0032891 | negative regulation of organic acid tran... | 12 | 12 | 12 | 0.00062 |
| GO:0021783 | preganglionic parasympathetic fiber deve... | 12 | 12 | 12 | 0.00062 |
| GO:0045921 | positive regulation of exocytosis | 55 | 55 | 55 | 0.00062 |
| GO:1901985 | positive regulation of protein acetylati... | 27 | 27 | 27 | 0.00062 |
| GO:1902749 | regulation of cell cycle G2/M phase tran... | 40 | 40 | 40 | 0.00062 |
| GO:0006694 | steroid biosynthetic process | 90 | 90 | 90 | 0.00063 |
| GO:1901017 | negative regulation of potassium ion tra... | 11 | 11 | 11 | 0.00063 |
| GO:0045840 | positive regulation of mitotic nuclear d... | 32 | 32 | 32 | 0.00063 |
| GO:0070841 | inclusion body assembly | 17 | 17 | 17 | 0.00064 |
| GO:1990267 | response to transition metal nanoparticl... | 77 | 77 | 77 | 0.00064 |
| GO:0043981 | histone H4-K5 acetylation | 13 | 13 | 13 | 0.00064 |
| GO:0043982 | histone H4-K8 acetylation | 13 | 13 | 13 | 0.00064 |
| GO:0046320 | regulation of fatty acid oxidation | 20 | 20 | 20 | 0.00064 |
| GO:0050906 | detection of stimulus involved in sensor... | 44 | 44 | 44 | 0.00064 |
| GO:0009409 | response to cold | 32 | 32 | 32 | 0.00064 |
| GO:0097105 | presynaptic membrane assembly | 9 | 9 | 9 | 0.00064 |
| GO:0031998 | regulation of fatty acid beta-oxidation | 11 | 11 | 11 | 0.00065 |
| GO:0033137 | negative regulation of peptidyl-serine p... | 17 | 17 | 17 | 0.00065 |
| GO:0030252 | growth hormone secretion | 11 | 11 | 11 | 0.00065 |
| GO:0021846 | cell proliferation in forebrain | 22 | 22 | 22 | 0.00065 |
| GO:0043922 | negative regulation by host of viral tra... | 8 | 8 | 8 | 0.00065 |
| GO:0002753 | cytoplasmic pattern recognition receptor... | 38 | 38 | 38 | 0.00066 |
| GO:0044804 | nucleophagy | 17 | 17 | 17 | 0.00066 |
| GO:0045606 | positive regulation of epidermal cell di... | 12 | 12 | 12 | 0.00066 |
| GO:0010692 | regulation of alkaline phosphatase activ... | 8 | 8 | 8 | 0.00066 |
| GO:0048755 | branching morphogenesis of a nerve | 8 | 8 | 8 | 0.00066 |
| GO:0050818 | regulation of coagulation | 59 | 59 | 59 | 0.00066 |
| GO:0072176 | nephric duct development | 14 | 14 | 14 | 0.00067 |
| GO:0048799 | organ maturation | 19 | 19 | 19 | 0.00067 |
| GO:0051785 | positive regulation of nuclear division | 39 | 39 | 39 | 0.00067 |
| GO:0072234 | metanephric nephron tubule development | 18 | 18 | 18 | 0.00067 |
| GO:0072243 | metanephric nephron epithelium developme... | 18 | 18 | 18 | 0.00067 |
| GO:0050654 | chondroitin sulfate proteoglycan metabol... | 46 | 46 | 46 | 0.00068 |
| GO:0042742 | defense response to bacterium | 87 | 87 | 87 | 0.00068 |
| GO:0051058 | negative regulation of small GTPase medi... | 36 | 36 | 36 | 0.00068 |
| GO:0001660 | fever generation | 7 | 7 | 7 | 0.00068 |
| GO:0042311 | vasodilation | 44 | 44 | 44 | 0.00068 |
| GO:1900024 | regulation of substrate adhesion-depende... | 35 | 35 | 35 | 0.00069 |
| GO:0035518 | histone H2A monoubiquitination | 10 | 10 | 10 | 0.00069 |
| GO:0031620 | regulation of fever generation | 6 | 6 | 6 | 0.00069 |
| GO:0001958 | endochondral ossification | 24 | 24 | 24 | 0.00070 |
| GO:0036075 | replacement ossification | 24 | 24 | 24 | 0.00070 |
| GO:0033045 | regulation of sister chromatid segregati... | 37 | 37 | 37 | 0.00070 |
| GO:0033047 | regulation of mitotic sister chromatid s... | 37 | 37 | 37 | 0.00070 |
| GO:2000696 | regulation of epithelial cell differenti... | 12 | 12 | 12 | 0.00070 |
| GO:0070170 | regulation of tooth mineralization | 5 | 5 | 5 | 0.00070 |
| GO:0021978 | telencephalon regionalization | 10 | 10 | 10 | 0.00070 |
| GO:0045742 | positive regulation of epidermal growth ... | 14 | 14 | 14 | 0.00070 |
| GO:0097201 | negative regulation of transcription fro... | 6 | 6 | 6 | 0.00070 |
| GO:0021877 | forebrain neuron fate commitment | 12 | 12 | 12 | 0.00071 |
| GO:0034394 | protein localization to cell surface | 33 | 33 | 33 | 0.00071 |
| GO:0018230 | peptidyl-L-cysteine S-palmitoylation | 15 | 15 | 15 | 0.00071 |
| GO:0018231 | peptidyl-S-diacylglycerol-L-cysteine bio... | 15 | 15 | 15 | 0.00071 |
| GO:0060325 | face morphogenesis | 27 | 27 | 27 | 0.00071 |
| GO:0007595 | lactation | 32 | 32 | 32 | 0.00071 |
| GO:0002328 | pro-B cell differentiation | 8 | 8 | 8 | 0.00071 |
| GO:0030852 | regulation of granulocyte differentiatio... | 12 | 12 | 12 | 0.00072 |
| GO:0018027 | peptidyl-lysine dimethylation | 9 | 9 | 9 | 0.00073 |
| GO:0006895 | Golgi to endosome transport | 16 | 16 | 16 | 0.00073 |
| GO:0051926 | negative regulation of calcium ion trans... | 29 | 29 | 29 | 0.00075 |
| GO:0021859 | pyramidal neuron differentiation | 7 | 7 | 7 | 0.00075 |
| GO:0016577 | histone demethylation | 22 | 22 | 22 | 0.00075 |
| GO:0021692 | cerebellar Purkinje cell layer morphogen... | 14 | 14 | 14 | 0.00076 |
| GO:1901032 | negative regulation of response to react... | 7 | 7 | 7 | 0.00077 |
| GO:1903205 | regulation of hydrogen peroxide-induced ... | 7 | 7 | 7 | 0.00077 |
| GO:0035930 | corticosteroid hormone secretion | 11 | 11 | 11 | 0.00077 |
| GO:1903317 | regulation of protein maturation | 48 | 48 | 48 | 0.00077 |
| GO:0045136 | development of secondary sexual characte... | 9 | 9 | 9 | 0.00077 |
| GO:0044088 | regulation of vacuole organization | 29 | 29 | 29 | 0.00078 |
| GO:0048066 | developmental pigmentation | 30 | 30 | 30 | 0.00078 |
| GO:0090504 | epiboly | 21 | 21 | 21 | 0.00078 |
| GO:2000377 | regulation of reactive oxygen species me... | 104 | 104 | 104 | 0.00079 |
| GO:0060561 | apoptotic process involved in morphogene... | 24 | 24 | 24 | 0.00079 |
| GO:0085029 | extracellular matrix assembly | 23 | 23 | 23 | 0.00079 |
| GO:0002200 | somatic diversification of immune recept... | 38 | 38 | 38 | 0.00079 |
| GO:0010573 | vascular endothelial growth factor produ... | 22 | 22 | 22 | 0.00079 |
| GO:0048741 | skeletal muscle fiber development | 20 | 20 | 20 | 0.00079 |
| GO:0048821 | erythrocyte development | 22 | 22 | 22 | 0.00080 |
| GO:0060412 | ventricular septum morphogenesis | 22 | 22 | 22 | 0.00080 |
| GO:0046677 | response to antibiotic | 34 | 34 | 34 | 0.00080 |
| GO:0086002 | cardiac muscle cell action potential inv... | 28 | 28 | 28 | 0.00081 |
| GO:0044342 | type B pancreatic cell proliferation | 15 | 15 | 15 | 0.00082 |
| GO:0060839 | endothelial cell fate commitment | 7 | 7 | 7 | 0.00082 |
| GO:0060252 | positive regulation of glial cell prolif... | 9 | 9 | 9 | 0.00082 |
| GO:0072079 | nephron tubule formation | 17 | 17 | 17 | 0.00082 |
| GO:0048745 | smooth muscle tissue development | 17 | 17 | 17 | 0.00082 |
| GO:0061298 | retina vasculature development in camera... | 17 | 17 | 17 | 0.00082 |
| GO:0042254 | ribosome biogenesis | 95 | 95 | 95 | 0.00083 |
| GO:0006821 | chloride transport | 73 | 73 | 73 | 0.00083 |
| GO:0010614 | negative regulation of cardiac muscle hy... | 10 | 10 | 10 | 0.00083 |
| GO:0060479 | lung cell differentiation | 25 | 25 | 25 | 0.00083 |
| GO:0033522 | histone H2A ubiquitination | 17 | 17 | 17 | 0.00083 |
| GO:0002367 | cytokine production involved in immune r... | 36 | 36 | 36 | 0.00083 |
| GO:0098900 | regulation of action potential | 26 | 26 | 26 | 0.00083 |
| GO:0007289 | spermatid nucleus differentiation | 15 | 15 | 15 | 0.00084 |
| GO:0051304 | chromosome separation | 42 | 42 | 42 | 0.00084 |
| GO:2000758 | positive regulation of peptidyl-lysine a... | 23 | 23 | 23 | 0.00084 |
| GO:0071599 | otic vesicle development | 12 | 12 | 12 | 0.00085 |
| GO:0051782 | negative regulation of cell division | 50 | 50 | 50 | 0.00085 |
| GO:0002673 | regulation of acute inflammatory respons... | 38 | 38 | 38 | 0.00085 |
| GO:0003207 | cardiac chamber formation | 9 | 9 | 9 | 0.00085 |
| GO:0042921 | glucocorticoid receptor signaling pathwa... | 16 | 16 | 16 | 0.00086 |
| GO:0006491 | N-glycan processing | 14 | 14 | 14 | 0.00086 |
| GO:0097503 | sialylation | 20 | 20 | 20 | 0.00086 |
| GO:0090382 | phagosome maturation | 28 | 28 | 28 | 0.00087 |
| GO:0050927 | positive regulation of positive chemotax... | 17 | 17 | 17 | 0.00087 |
| GO:0070266 | necroptotic process | 25 | 25 | 25 | 0.00087 |
| GO:0021591 | ventricular system development | 21 | 21 | 21 | 0.00088 |
| GO:0070933 | histone H4 deacetylation | 10 | 10 | 10 | 0.00088 |
| GO:0019228 | neuronal action potential | 28 | 28 | 28 | 0.00088 |
| GO:0032480 | negative regulation of type I interferon... | 26 | 26 | 26 | 0.00088 |
| GO:0048599 | oocyte development | 18 | 18 | 18 | 0.00088 |
| GO:0071364 | cellular response to epidermal growth fa... | 19 | 19 | 19 | 0.00088 |
| GO:1902117 | positive regulation of organelle assembl... | 38 | 38 | 38 | 0.00089 |
| GO:2000465 | regulation of glycogen (starch) synthase... | 6 | 6 | 6 | 0.00090 |
| GO:0009994 | oocyte differentiation | 20 | 20 | 20 | 0.00090 |
| GO:0035872 | nucleotide-binding domain  leucine rich ... | 35 | 35 | 35 | 0.00090 |
| GO:0006337 | nucleosome disassembly | 15 | 15 | 15 | 0.00091 |
| GO:0032986 | protein-DNA complex disassembly | 15 | 15 | 15 | 0.00091 |
| GO:0050810 | regulation of steroid biosynthetic proce... | 37 | 37 | 37 | 0.00091 |
| GO:0072497 | mesenchymal stem cell differentiation | 7 | 7 | 7 | 0.00091 |
| GO:0036296 | response to increased oxygen levels | 19 | 19 | 19 | 0.00091 |
| GO:0055093 | response to hyperoxia | 19 | 19 | 19 | 0.00091 |
| GO:0002701 | negative regulation of production of mol... | 11 | 11 | 11 | 0.00091 |
| GO:2000794 | regulation of epithelial cell proliferat... | 7 | 7 | 7 | 0.00093 |
| GO:0072498 | embryonic skeletal joint development | 12 | 12 | 12 | 0.00093 |
| GO:0048679 | regulation of axon regeneration | 13 | 13 | 13 | 0.00093 |
| GO:1900120 | regulation of receptor binding | 13 | 13 | 13 | 0.00093 |
| GO:0034470 | ncRNA processing | 135 | 135 | 135 | 0.00094 |
| GO:0035384 | thioester biosynthetic process | 29 | 29 | 29 | 0.00094 |
| GO:0071616 | acyl-CoA biosynthetic process | 29 | 29 | 29 | 0.00094 |
| GO:0072170 | metanephric tubule development | 20 | 20 | 20 | 0.00094 |
| GO:0070828 | heterochromatin organization | 10 | 10 | 10 | 0.00094 |
| GO:0035987 | endodermal cell differentiation | 37 | 37 | 37 | 0.00095 |
| GO:0071205 | protein localization to juxtaparanode re... | 5 | 5 | 5 | 0.00095 |
| GO:0007193 | adenylate cyclase-inhibiting G-protein c... | 44 | 44 | 44 | 0.00095 |
| GO:0001946 | lymphangiogenesis | 12 | 12 | 12 | 0.00096 |
| GO:0003416 | endochondral bone growth | 16 | 16 | 16 | 0.00096 |
| GO:0001937 | negative regulation of endothelial cell ... | 22 | 22 | 22 | 0.00097 |
| GO:0090501 | RNA phosphodiester bond hydrolysis | 61 | 61 | 61 | 0.00097 |
| GO:0043536 | positive regulation of blood vessel endo... | 13 | 13 | 13 | 0.00097 |
| GO:0097120 | receptor localization to synapse | 7 | 7 | 7 | 0.00098 |
| GO:0030913 | paranodal junction assembly | 5 | 5 | 5 | 0.00098 |
| GO:0030818 | negative regulation of cAMP biosynthetic... | 24 | 24 | 24 | 0.00099 |
| GO:0050886 | endocrine process | 45 | 45 | 45 | 0.00099 |
| GO:0016126 | sterol biosynthetic process | 33 | 33 | 33 | 0.00100 |
| GO:0090277 | positive regulation of peptide hormone s... | 53 | 53 | 53 | 0.00101 |
| GO:0043537 | negative regulation of blood vessel endo... | 15 | 15 | 15 | 0.00101 |
| GO:0050435 | beta-amyloid metabolic process | 16 | 16 | 16 | 0.00101 |
| GO:0022400 | regulation of rhodopsin mediated signali... | 21 | 21 | 21 | 0.00102 |
| GO:1902743 | regulation of lamellipodium organization | 31 | 31 | 31 | 0.00103 |
| GO:0032873 | negative regulation of stress-activated ... | 31 | 31 | 31 | 0.00103 |
| GO:0070303 | negative regulation of stress-activated ... | 31 | 31 | 31 | 0.00103 |
| GO:0042339 | keratan sulfate metabolic process | 27 | 27 | 27 | 0.00103 |
| GO:0032392 | DNA geometric change | 40 | 40 | 40 | 0.00104 |
| GO:0030262 | apoptotic nuclear changes | 21 | 21 | 21 | 0.00104 |
| GO:0033173 | calcineurin-NFAT signaling cascade | 20 | 20 | 20 | 0.00104 |
| GO:0036473 | cell death in response to oxidative stre... | 32 | 32 | 32 | 0.00104 |
| GO:0045010 | actin nucleation | 30 | 30 | 30 | 0.00105 |
| GO:0045333 | cellular respiration | 91 | 91 | 91 | 0.00106 |
| GO:0086091 | regulation of heart rate by cardiac cond... | 24 | 24 | 24 | 0.00106 |
| GO:0001941 | postsynaptic membrane organization | 22 | 22 | 22 | 0.00107 |
| GO:0000768 | syncytium formation by plasma membrane f... | 37 | 37 | 37 | 0.00107 |
| GO:0007260 | tyrosine phosphorylation of STAT protein | 40 | 40 | 40 | 0.00108 |
| GO:0042509 | regulation of tyrosine phosphorylation o... | 40 | 40 | 40 | 0.00108 |
| GO:0070972 | protein localization to endoplasmic reti... | 56 | 56 | 56 | 0.00108 |
| GO:0046710 | GDP metabolic process | 11 | 11 | 11 | 0.00109 |
| GO:0060122 | inner ear receptor stereocilium organiza... | 13 | 13 | 13 | 0.00109 |
| GO:0019915 | lipid storage | 39 | 39 | 39 | 0.00110 |
| GO:0034067 | protein localization to Golgi apparatus | 23 | 23 | 23 | 0.00110 |
| GO:0030007 | cellular potassium ion homeostasis | 10 | 10 | 10 | 0.00111 |
| GO:0090245 | axis elongation involved in somitogenesi... | 6 | 6 | 6 | 0.00111 |
| GO:0086019 | cell-cell signaling involved in cardiac ... | 16 | 16 | 16 | 0.00111 |
| GO:1901741 | positive regulation of myoblast fusion | 11 | 11 | 11 | 0.00112 |
| GO:0021522 | spinal cord motor neuron differentiation | 29 | 29 | 29 | 0.00112 |
| GO:0003323 | type B pancreatic cell development | 18 | 18 | 18 | 0.00112 |
| GO:0009988 | cell-cell recognition | 27 | 27 | 27 | 0.00114 |
| GO:0035116 | embryonic hindlimb morphogenesis | 26 | 26 | 26 | 0.00114 |
| GO:0090184 | positive regulation of kidney developmen... | 28 | 28 | 28 | 0.00114 |
| GO:1902692 | regulation of neuroblast proliferation | 23 | 23 | 23 | 0.00114 |
| GO:0046851 | negative regulation of bone remodeling | 8 | 8 | 8 | 0.00115 |
| GO:0055072 | iron ion homeostasis | 62 | 62 | 62 | 0.00115 |
| GO:0006622 | protein targeting to lysosome | 12 | 12 | 12 | 0.00115 |
| GO:0045494 | photoreceptor cell maintenance | 24 | 24 | 24 | 0.00116 |
| GO:0048278 | vesicle docking | 46 | 46 | 46 | 0.00116 |
| GO:2000192 | negative regulation of fatty acid transp... | 5 | 5 | 5 | 0.00117 |
| GO:0060143 | positive regulation of syncytium formati... | 16 | 16 | 16 | 0.00118 |
| GO:0060065 | uterus development | 15 | 15 | 15 | 0.00118 |
| GO:0021871 | forebrain regionalization | 18 | 18 | 18 | 0.00118 |
| GO:2000369 | regulation of clathrin-mediated endocyto... | 8 | 8 | 8 | 0.00119 |
| GO:0031116 | positive regulation of microtubule polym... | 12 | 12 | 12 | 0.00119 |
| GO:0033627 | cell adhesion mediated by integrin | 37 | 37 | 37 | 0.00119 |
| GO:0001676 | long-chain fatty acid metabolic process | 54 | 54 | 54 | 0.00119 |
| GO:0042417 | dopamine metabolic process | 21 | 21 | 21 | 0.00119 |
| GO:0072395 | signal transduction involved in cell cyc... | 41 | 41 | 41 | 0.00120 |
| GO:0046632 | alpha-beta T cell differentiation | 51 | 51 | 51 | 0.00120 |
| GO:0030595 | leukocyte chemotaxis | 100 | 100 | 100 | 0.00120 |
| GO:0051053 | negative regulation of DNA metabolic pro... | 47 | 47 | 47 | 0.00120 |
| GO:0003283 | atrial septum development | 14 | 14 | 14 | 0.00120 |
| GO:0046470 | phosphatidylcholine metabolic process | 41 | 41 | 41 | 0.00121 |
| GO:0021561 | facial nerve development | 7 | 7 | 7 | 0.00121 |
| GO:0021610 | facial nerve morphogenesis | 7 | 7 | 7 | 0.00121 |
| GO:0021612 | facial nerve structural organization | 7 | 7 | 7 | 0.00121 |
| GO:0005513 | detection of calcium ion | 11 | 11 | 11 | 0.00123 |
| GO:0030199 | collagen fibril organization | 36 | 36 | 36 | 0.00123 |
| GO:0042035 | regulation of cytokine biosynthetic proc... | 58 | 58 | 58 | 0.00123 |
| GO:0033169 | histone H3-K9 demethylation | 6 | 6 | 6 | 0.00124 |
| GO:0001945 | lymph vessel development | 21 | 21 | 21 | 0.00125 |
| GO:0051784 | negative regulation of nuclear division | 40 | 40 | 40 | 0.00125 |
| GO:0048733 | sebaceous gland development | 8 | 8 | 8 | 0.00125 |
| GO:0034341 | response to interferon-gamma | 85 | 85 | 85 | 0.00126 |
| GO:0009154 | purine ribonucleotide catabolic process | 22 | 22 | 22 | 0.00126 |
| GO:0032479 | regulation of type I interferon producti... | 70 | 70 | 70 | 0.00127 |
| GO:0071377 | cellular response to glucagon stimulus | 31 | 31 | 31 | 0.00128 |
| GO:0051642 | centrosome localization | 16 | 16 | 16 | 0.00128 |
| GO:0010971 | positive regulation of G2/M transition o... | 14 | 14 | 14 | 0.00128 |
| GO:1902751 | positive regulation of cell cycle G2/M p... | 14 | 14 | 14 | 0.00128 |
| GO:0070988 | demethylation | 37 | 37 | 37 | 0.00129 |
| GO:0098586 | cellular response to virus | 20 | 20 | 20 | 0.00129 |
| GO:0030049 | muscle filament sliding | 21 | 21 | 21 | 0.00129 |
| GO:0033275 | actin-myosin filament sliding | 21 | 21 | 21 | 0.00129 |
| GO:0018146 | keratan sulfate biosynthetic process | 24 | 24 | 24 | 0.00129 |
| GO:0090313 | regulation of protein targeting to membr... | 21 | 21 | 21 | 0.00130 |
| GO:0070527 | platelet aggregation | 40 | 40 | 40 | 0.00130 |
| GO:0071318 | cellular response to ATP | 6 | 6 | 6 | 0.00130 |
| GO:0046580 | negative regulation of Ras protein signa... | 35 | 35 | 35 | 0.00131 |
| GO:0014808 | release of sequestered calcium ion into ... | 19 | 19 | 19 | 0.00131 |
| GO:1903514 | calcium ion transport from endoplasmic r... | 19 | 19 | 19 | 0.00131 |
| GO:1903573 | negative regulation of response to endop... | 28 | 28 | 28 | 0.00131 |
| GO:0060445 | branching involved in salivary gland mor... | 21 | 21 | 21 | 0.00132 |
| GO:0048670 | regulation of collateral sprouting | 17 | 17 | 17 | 0.00133 |
| GO:0031365 | N-terminal protein amino acid modificati... | 20 | 20 | 20 | 0.00133 |
| GO:2000680 | regulation of rubidium ion transport | 4 | 4 | 4 | 0.00134 |
| GO:2000686 | regulation of rubidium ion transmembrane... | 4 | 4 | 4 | 0.00134 |
| GO:0060831 | smoothened signaling pathway involved in... | 11 | 11 | 11 | 0.00134 |
| GO:0050798 | activated T cell proliferation | 27 | 27 | 27 | 0.00134 |
| GO:0046037 | GMP metabolic process | 16 | 16 | 16 | 0.00135 |
| GO:2001212 | regulation of vasculogenesis | 11 | 11 | 11 | 0.00136 |
| GO:0016081 | synaptic vesicle docking | 8 | 8 | 8 | 0.00136 |
| GO:0001542 | ovulation from ovarian follicle | 8 | 8 | 8 | 0.00136 |
| GO:2000811 | negative regulation of anoikis | 13 | 13 | 13 | 0.00137 |
| GO:0016056 | rhodopsin mediated signaling pathway | 25 | 25 | 25 | 0.00137 |
| GO:0006654 | phosphatidic acid biosynthetic process | 18 | 18 | 18 | 0.00137 |
| GO:0046473 | phosphatidic acid metabolic process | 18 | 18 | 18 | 0.00137 |
| GO:1901021 | positive regulation of calcium ion trans... | 21 | 21 | 21 | 0.00137 |
| GO:1904427 | positive regulation of calcium ion trans... | 21 | 21 | 21 | 0.00137 |
| GO:0031063 | regulation of histone deacetylation | 15 | 15 | 15 | 0.00138 |
| GO:2001214 | positive regulation of vasculogenesis | 7 | 7 | 7 | 0.00139 |
| GO:0001959 | regulation of cytokine-mediated signalin... | 70 | 70 | 70 | 0.00139 |
| GO:0001782 | B cell homeostasis | 19 | 19 | 19 | 0.00139 |
| GO:0006885 | regulation of pH | 59 | 59 | 59 | 0.00139 |
| GO:0007091 | metaphase/anaphase transition of mitotic... | 36 | 36 | 36 | 0.00140 |
| GO:0010965 | regulation of mitotic sister chromatid s... | 36 | 36 | 36 | 0.00140 |
| GO:0044784 | metaphase/anaphase transition of cell cy... | 36 | 36 | 36 | 0.00140 |
| GO:0051306 | mitotic sister chromatid separation | 36 | 36 | 36 | 0.00140 |
| GO:0034314 | Arp2/3 complex-mediated actin nucleation | 22 | 22 | 22 | 0.00140 |
| GO:0051386 | regulation of neurotrophin TRK receptor ... | 6 | 6 | 6 | 0.00140 |
| GO:2000050 | regulation of non-canonical Wnt signalin... | 13 | 13 | 13 | 0.00141 |
| GO:0046425 | regulation of JAK-STAT cascade | 69 | 69 | 69 | 0.00141 |
| GO:0042297 | vocal learning | 5 | 5 | 5 | 0.00142 |
| GO:0098596 | imitative learning | 5 | 5 | 5 | 0.00142 |
| GO:0098598 | learned vocalization behavior or vocal l... | 5 | 5 | 5 | 0.00142 |
| GO:0009062 | fatty acid catabolic process | 56 | 56 | 56 | 0.00142 |
| GO:0032288 | myelin assembly | 16 | 16 | 16 | 0.00143 |
| GO:0042461 | photoreceptor cell development | 25 | 25 | 25 | 0.00143 |
| GO:1903202 | negative regulation of oxidative stress-... | 16 | 16 | 16 | 0.00144 |
| GO:0042487 | regulation of odontogenesis of dentin-co... | 9 | 9 | 9 | 0.00144 |
| GO:0042048 | olfactory behavior | 5 | 5 | 5 | 0.00146 |
| GO:2000379 | positive regulation of reactive oxygen s... | 59 | 59 | 59 | 0.00147 |
| GO:0002703 | regulation of leukocyte mediated immunit... | 80 | 80 | 80 | 0.00147 |
| GO:0072210 | metanephric nephron development | 33 | 33 | 33 | 0.00147 |
| GO:0033059 | cellular pigmentation | 28 | 28 | 28 | 0.00147 |
| GO:1900746 | regulation of vascular endothelial growt... | 8 | 8 | 8 | 0.00147 |
| GO:0010603 | regulation of cytoplasmic mRNA processin... | 8 | 8 | 8 | 0.00148 |
| GO:2000677 | regulation of transcription regulatory r... | 27 | 27 | 27 | 0.00148 |
| GO:0010738 | regulation of protein kinase A signaling | 11 | 11 | 11 | 0.00149 |
| GO:0030540 | female genitalia development | 12 | 12 | 12 | 0.00149 |
| GO:0042462 | eye photoreceptor cell development | 20 | 20 | 20 | 0.00149 |
| GO:0003159 | morphogenesis of an endothelium | 11 | 11 | 11 | 0.00149 |
| GO:0001573 | ganglioside metabolic process | 20 | 20 | 20 | 0.00149 |
| GO:0072523 | purine-containing compound catabolic pro... | 34 | 34 | 34 | 0.00150 |
| GO:0048668 | collateral sprouting | 19 | 19 | 19 | 0.00150 |
| GO:0051125 | regulation of actin nucleation | 17 | 17 | 17 | 0.00151 |
| GO:0030517 | negative regulation of axon extension | 19 | 19 | 19 | 0.00151 |
| GO:0010324 | membrane invagination | 25 | 25 | 25 | 0.00151 |
| GO:0010390 | histone monoubiquitination | 19 | 19 | 19 | 0.00152 |
| GO:0035886 | vascular smooth muscle cell differentiat... | 12 | 12 | 12 | 0.00152 |
| GO:0051127 | positive regulation of actin nucleation | 10 | 10 | 10 | 0.00152 |
| GO:0045600 | positive regulation of fat cell differen... | 38 | 38 | 38 | 0.00153 |
| GO:0002052 | positive regulation of neuroblast prolif... | 15 | 15 | 15 | 0.00153 |
| GO:0090200 | positive regulation of release of cytoch... | 16 | 16 | 16 | 0.00153 |
| GO:0031498 | chromatin disassembly | 16 | 16 | 16 | 0.00153 |
| GO:0060272 | embryonic skeletal joint morphogenesis | 11 | 11 | 11 | 0.00153 |
| GO:0072178 | nephric duct morphogenesis | 11 | 11 | 11 | 0.00153 |
| GO:0072182 | regulation of nephron tubule epithelial ... | 11 | 11 | 11 | 0.00153 |
| GO:0032516 | positive regulation of phosphoprotein ph... | 11 | 11 | 11 | 0.00153 |
| GO:0045475 | locomotor rhythm | 10 | 10 | 10 | 0.00153 |
| GO:0006879 | cellular iron ion homeostasis | 42 | 42 | 42 | 0.00156 |
| GO:0021796 | cerebral cortex regionalization | 7 | 7 | 7 | 0.00157 |
| GO:0032508 | DNA duplex unwinding | 35 | 35 | 35 | 0.00157 |
| GO:0030800 | negative regulation of cyclic nucleotide... | 29 | 29 | 29 | 0.00157 |
| GO:0030193 | regulation of blood coagulation | 57 | 57 | 57 | 0.00158 |
| GO:1900046 | regulation of hemostasis | 57 | 57 | 57 | 0.00158 |
| GO:0060976 | coronary vasculature development | 13 | 13 | 13 | 0.00158 |
| GO:0007220 | Notch receptor processing | 15 | 15 | 15 | 0.00158 |
| GO:0090178 | regulation of establishment of planar po... | 10 | 10 | 10 | 0.00159 |
| GO:0090179 | planar cell polarity pathway involved in... | 10 | 10 | 10 | 0.00159 |
| GO:0010575 | positive regulation of vascular endothel... | 18 | 18 | 18 | 0.00159 |
| GO:2000144 | positive regulation of DNA-templated tra... | 17 | 17 | 17 | 0.00160 |
| GO:1903859 | regulation of dendrite extension | 13 | 13 | 13 | 0.00161 |
| GO:1903861 | positive regulation of dendrite extensio... | 13 | 13 | 13 | 0.00161 |
| GO:0016266 | O-glycan processing | 41 | 41 | 41 | 0.00161 |
| GO:0060306 | regulation of membrane repolarization | 22 | 22 | 22 | 0.00161 |
| GO:0046427 | positive regulation of JAK-STAT cascade | 45 | 45 | 45 | 0.00161 |
| GO:0071526 | semaphorin-plexin signaling pathway | 19 | 19 | 19 | 0.00161 |
| GO:0030204 | chondroitin sulfate metabolic process | 44 | 44 | 44 | 0.00162 |
| GO:0046634 | regulation of alpha-beta T cell activati... | 40 | 40 | 40 | 0.00162 |
| GO:0010944 | negative regulation of transcription by ... | 7 | 7 | 7 | 0.00162 |
| GO:1901381 | positive regulation of potassium ion tra... | 20 | 20 | 20 | 0.00163 |
| GO:0031987 | locomotion involved in locomotory behavi... | 6 | 6 | 6 | 0.00163 |
| GO:0035066 | positive regulation of histone acetylati... | 22 | 22 | 22 | 0.00163 |
| GO:0019068 | virion assembly | 26 | 26 | 26 | 0.00163 |
| GO:0060767 | epithelial cell proliferation involved i... | 6 | 6 | 6 | 0.00167 |
| GO:0021546 | rhombomere development | 5 | 5 | 5 | 0.00168 |
| GO:0048845 | venous blood vessel morphogenesis | 8 | 8 | 8 | 0.00169 |
| GO:0010718 | positive regulation of epithelial to mes... | 26 | 26 | 26 | 0.00169 |
| GO:0051569 | regulation of histone H3-K4 methylation | 21 | 21 | 21 | 0.00170 |
| GO:0002762 | negative regulation of myeloid leukocyte... | 27 | 27 | 27 | 0.00170 |
| GO:0010837 | regulation of keratinocyte proliferation | 20 | 20 | 20 | 0.00170 |
| GO:0031648 | protein destabilization | 27 | 27 | 27 | 0.00171 |
| GO:2001241 | positive regulation of extrinsic apoptot... | 10 | 10 | 10 | 0.00171 |
| GO:0046321 | positive regulation of fatty acid oxidat... | 11 | 11 | 11 | 0.00171 |
| GO:0048170 | positive regulation of long-term neurona... | 4 | 4 | 4 | 0.00171 |
| GO:1901976 | regulation of cell cycle checkpoint | 18 | 18 | 18 | 0.00171 |
| GO:0031503 | protein complex localization | 48 | 48 | 48 | 0.00172 |
| GO:0001840 | neural plate development | 9 | 9 | 9 | 0.00173 |
| GO:0032606 | type I interferon production | 71 | 71 | 71 | 0.00173 |
| GO:0051881 | regulation of mitochondrial membrane pot... | 32 | 32 | 32 | 0.00173 |
| GO:0033363 | secretory granule organization | 19 | 19 | 19 | 0.00174 |
| GO:0071470 | cellular response to osmotic stress | 20 | 20 | 20 | 0.00175 |
| GO:0044319 | wound healing  spreading of cells | 20 | 20 | 20 | 0.00175 |
| GO:0090505 | epiboly involved in wound healing | 20 | 20 | 20 | 0.00175 |
| GO:0060837 | blood vessel endothelial cell differenti... | 8 | 8 | 8 | 0.00175 |
| GO:0002285 | lymphocyte activation involved in immune... | 82 | 82 | 82 | 0.00175 |
| GO:0043616 | keratinocyte proliferation | 26 | 26 | 26 | 0.00176 |
| GO:0030815 | negative regulation of cAMP metabolic pr... | 27 | 27 | 27 | 0.00177 |
| GO:0002706 | regulation of lymphocyte mediated immuni... | 57 | 57 | 57 | 0.00178 |
| GO:0034661 | ncRNA catabolic process | 13 | 13 | 13 | 0.00179 |
| GO:0036005 | response to macrophage colony-stimulatin... | 8 | 8 | 8 | 0.00179 |
| GO:0036006 | cellular response to macrophage colony-s... | 8 | 8 | 8 | 0.00179 |
| GO:0001662 | behavioral fear response | 27 | 27 | 27 | 0.00180 |
| GO:0002209 | behavioral defense response | 27 | 27 | 27 | 0.00180 |
| GO:0031114 | regulation of microtubule depolymerizati... | 17 | 17 | 17 | 0.00180 |
| GO:0060037 | pharyngeal system development | 14 | 14 | 14 | 0.00181 |
| GO:1901739 | regulation of myoblast fusion | 13 | 13 | 13 | 0.00181 |
| GO:0015837 | amine transport | 49 | 49 | 49 | 0.00181 |
| GO:0006977 | DNA damage response  signal transduction... | 38 | 38 | 38 | 0.00181 |
| GO:0072431 | signal transduction involved in mitotic ... | 38 | 38 | 38 | 0.00181 |
| GO:1902400 | intracellular signal transduction involv... | 38 | 38 | 38 | 0.00181 |
| GO:0007026 | negative regulation of microtubule depol... | 15 | 15 | 15 | 0.00182 |
| GO:0048227 | plasma membrane to endosome transport | 6 | 6 | 6 | 0.00184 |
| GO:0090336 | positive regulation of brown fat cell di... | 5 | 5 | 5 | 0.00186 |
| GO:0050765 | negative regulation of phagocytosis | 4 | 4 | 4 | 0.00187 |
| GO:0031622 | positive regulation of fever generation | 5 | 5 | 5 | 0.00188 |
| GO:0042755 | eating behavior | 16 | 16 | 16 | 0.00189 |
| GO:0010452 | histone H3-K36 methylation | 7 | 7 | 7 | 0.00190 |
| GO:0050919 | negative chemotaxis | 15 | 15 | 15 | 0.00190 |
| GO:0050853 | B cell receptor signaling pathway | 27 | 27 | 27 | 0.00191 |
| GO:0009247 | glycolipid biosynthetic process | 44 | 44 | 44 | 0.00191 |
| GO:0045948 | positive regulation of translational ini... | 10 | 10 | 10 | 0.00192 |
| GO:0040020 | regulation of meiotic nuclear division | 17 | 17 | 17 | 0.00192 |
| GO:0060575 | intestinal epithelial cell differentiati... | 13 | 13 | 13 | 0.00193 |
| GO:0050974 | detection of mechanical stimulus involve... | 16 | 16 | 16 | 0.00193 |
| GO:1900103 | positive regulation of endoplasmic retic... | 9 | 9 | 9 | 0.00194 |
| GO:0090218 | positive regulation of lipid kinase acti... | 25 | 25 | 25 | 0.00195 |
| GO:0032808 | lacrimal gland development | 7 | 7 | 7 | 0.00196 |
| GO:0098597 | observational learning | 7 | 7 | 7 | 0.00197 |
| GO:0030803 | negative regulation of cyclic nucleotide... | 25 | 25 | 25 | 0.00197 |
| GO:0006949 | syncytium formation | 38 | 38 | 38 | 0.00198 |
| GO:0010842 | retina layer formation | 16 | 16 | 16 | 0.00199 |
| GO:0021860 | pyramidal neuron development | 6 | 6 | 6 | 0.00199 |
| GO:0060665 | regulation of branching involved in sali... | 4 | 4 | 4 | 0.00201 |
| GO:0048199 | vesicle targeting to  from or within Go... | 23 | 23 | 23 | 0.00201 |
| GO:0003284 | septum primum development | 4 | 4 | 4 | 0.00201 |
| GO:0003289 | atrial septum primum morphogenesis | 4 | 4 | 4 | 0.00201 |
| GO:0070534 | protein K63-linked ubiquitination | 32 | 32 | 32 | 0.00201 |
| GO:1903409 | reactive oxygen species biosynthetic pro... | 54 | 54 | 54 | 0.00202 |
| GO:0070857 | regulation of bile acid biosynthetic pro... | 4 | 4 | 4 | 0.00202 |
| GO:0030889 | negative regulation of B cell proliferat... | 10 | 10 | 10 | 0.00202 |
| GO:0000737 | DNA catabolic process  endonucleolytic | 16 | 16 | 16 | 0.00203 |
| GO:0006198 | cAMP catabolic process | 17 | 17 | 17 | 0.00204 |
| GO:0010574 | regulation of vascular endothelial growt... | 21 | 21 | 21 | 0.00205 |
| GO:1902237 | positive regulation of endoplasmic retic... | 10 | 10 | 10 | 0.00205 |
| GO:0031547 | brain-derived neurotrophic factor recept... | 4 | 4 | 4 | 0.00205 |
| GO:0090049 | regulation of cell migration involved in... | 11 | 11 | 11 | 0.00206 |
| GO:0045820 | negative regulation of glycolytic proces... | 8 | 8 | 8 | 0.00206 |
| GO:0090085 | regulation of protein deubiquitination | 6 | 6 | 6 | 0.00207 |
| GO:0006111 | regulation of gluconeogenesis | 25 | 25 | 25 | 0.00207 |
| GO:0061217 | regulation of mesonephros development | 21 | 21 | 21 | 0.00207 |
| GO:0070305 | response to cGMP | 7 | 7 | 7 | 0.00208 |
| GO:1902166 | negative regulation of intrinsic apoptot... | 8 | 8 | 8 | 0.00210 |
| GO:0002562 | somatic diversification of immune recept... | 35 | 35 | 35 | 0.00210 |
| GO:0016444 | somatic cell DNA recombination | 35 | 35 | 35 | 0.00210 |
| GO:0042474 | middle ear morphogenesis | 18 | 18 | 18 | 0.00210 |
| GO:0001574 | ganglioside biosynthetic process | 17 | 17 | 17 | 0.00210 |
| GO:0060487 | lung epithelial cell differentiation | 24 | 24 | 24 | 0.00210 |
| GO:0007603 | phototransduction  visible light | 56 | 56 | 56 | 0.00212 |
| GO:2000136 | regulation of cell proliferation involve... | 12 | 12 | 12 | 0.00213 |
| GO:2000741 | positive regulation of mesenchymal stem ... | 4 | 4 | 4 | 0.00213 |
| GO:0098657 | import into cell | 22 | 22 | 22 | 0.00214 |
| GO:0061001 | regulation of dendritic spine morphogene... | 23 | 23 | 23 | 0.00214 |
| GO:0060502 | epithelial cell proliferation involved i... | 8 | 8 | 8 | 0.00215 |
| GO:0034063 | stress granule assembly | 6 | 6 | 6 | 0.00216 |
| GO:0042531 | positive regulation of tyrosine phosphor... | 35 | 35 | 35 | 0.00218 |
| GO:0032784 | regulation of DNA-templated transcriptio... | 28 | 28 | 28 | 0.00218 |
| GO:0021873 | forebrain neuroblast division | 6 | 6 | 6 | 0.00219 |
| GO:2000311 | regulation of alpha-amino-3-hydroxy-5-me... | 16 | 16 | 16 | 0.00219 |
| GO:0046621 | negative regulation of organ growth | 18 | 18 | 18 | 0.00220 |
| GO:1902992 | negative regulation of amyloid precursor... | 6 | 6 | 6 | 0.00220 |
| GO:0048730 | epidermis morphogenesis | 27 | 27 | 27 | 0.00220 |
| GO:0003215 | cardiac right ventricle morphogenesis | 13 | 13 | 13 | 0.00220 |
| GO:0032878 | regulation of establishment or maintenan... | 17 | 17 | 17 | 0.00221 |
| GO:0035313 | wound healing  spreading of epidermal ce... | 13 | 13 | 13 | 0.00221 |
| GO:0007398 | ectoderm development | 18 | 18 | 18 | 0.00222 |
| GO:0070265 | necrotic cell death | 35 | 35 | 35 | 0.00223 |
| GO:0055075 | potassium ion homeostasis | 21 | 21 | 21 | 0.00225 |
| GO:0042220 | response to cocaine | 37 | 37 | 37 | 0.00225 |
| GO:0021544 | subpallium development | 17 | 17 | 17 | 0.00225 |
| GO:0032786 | positive regulation of DNA-templated tra... | 16 | 16 | 16 | 0.00226 |
| GO:0051531 | NFAT protein import into nucleus | 14 | 14 | 14 | 0.00227 |
| GO:0060571 | morphogenesis of an epithelial fold | 19 | 19 | 19 | 0.00228 |
| GO:0045713 | low-density lipoprotein particle recepto... | 9 | 9 | 9 | 0.00229 |
| GO:0045621 | positive regulation of lymphocyte differ... | 56 | 56 | 56 | 0.00229 |
| GO:0034773 | histone H4-K20 trimethylation | 5 | 5 | 5 | 0.00230 |
| GO:0031338 | regulation of vesicle fusion | 13 | 13 | 13 | 0.00230 |
| GO:0030201 | heparan sulfate proteoglycan metabolic p... | 26 | 26 | 26 | 0.00230 |
| GO:0035881 | amacrine cell differentiation | 7 | 7 | 7 | 0.00231 |
| GO:0010586 | miRNA metabolic process | 13 | 13 | 13 | 0.00231 |
| GO:1902476 | chloride transmembrane transport | 66 | 66 | 66 | 0.00231 |
| GO:0010893 | positive regulation of steroid biosynthe... | 11 | 11 | 11 | 0.00232 |
| GO:0031670 | cellular response to nutrient | 31 | 31 | 31 | 0.00232 |
| GO:2000973 | regulation of pro-B cell differentiation | 6 | 6 | 6 | 0.00232 |
| GO:0010224 | response to UV-B | 12 | 12 | 12 | 0.00232 |
| GO:0032527 | protein exit from endoplasmic reticulum | 26 | 26 | 26 | 0.00233 |
| GO:0051293 | establishment of spindle localization | 26 | 26 | 26 | 0.00234 |
| GO:0010470 | regulation of gastrulation | 28 | 28 | 28 | 0.00234 |
| GO:0090162 | establishment of epithelial cell polarit... | 16 | 16 | 16 | 0.00234 |
| GO:0060713 | labyrinthine layer morphogenesis | 16 | 16 | 16 | 0.00234 |
| GO:0072401 | signal transduction involved in DNA inte... | 40 | 40 | 40 | 0.00235 |
| GO:0072422 | signal transduction involved in DNA dama... | 40 | 40 | 40 | 0.00235 |
| GO:0070231 | T cell apoptotic process | 29 | 29 | 29 | 0.00235 |
| GO:0006482 | protein demethylation | 24 | 24 | 24 | 0.00235 |
| GO:0008214 | protein dealkylation | 24 | 24 | 24 | 0.00235 |
| GO:0060323 | head morphogenesis | 33 | 33 | 33 | 0.00237 |
| GO:0090129 | positive regulation of synapse maturatio... | 7 | 7 | 7 | 0.00237 |
| GO:0006471 | protein ADP-ribosylation | 14 | 14 | 14 | 0.00237 |
| GO:0061045 | negative regulation of wound healing | 43 | 43 | 43 | 0.00239 |
| GO:0045839 | negative regulation of mitotic nuclear d... | 34 | 34 | 34 | 0.00239 |
| GO:0009584 | detection of visible light | 66 | 66 | 66 | 0.00241 |
| GO:1901797 | negative regulation of signal transducti... | 18 | 18 | 18 | 0.00242 |
| GO:2000772 | regulation of cellular senescence | 22 | 22 | 22 | 0.00245 |
| GO:0044331 | cell-cell adhesion mediated by cadherin | 10 | 10 | 10 | 0.00246 |
| GO:0007567 | parturition | 10 | 10 | 10 | 0.00246 |
| GO:0022406 | membrane docking | 55 | 55 | 55 | 0.00247 |
| GO:0003143 | embryonic heart tube morphogenesis | 44 | 44 | 44 | 0.00248 |
| GO:0003181 | atrioventricular valve morphogenesis | 13 | 13 | 13 | 0.00248 |
| GO:0002087 | regulation of respiratory gaseous exchan... | 12 | 12 | 12 | 0.00248 |
| GO:0044065 | regulation of respiratory system process | 12 | 12 | 12 | 0.00248 |
| GO:0060080 | inhibitory postsynaptic potential | 12 | 12 | 12 | 0.00248 |
| GO:0060836 | lymphatic endothelial cell differentiati... | 5 | 5 | 5 | 0.00249 |
| GO:0042398 | cellular modified amino acid biosyntheti... | 31 | 31 | 31 | 0.00251 |
| GO:0051570 | regulation of histone H3-K9 methylation | 10 | 10 | 10 | 0.00252 |
| GO:0006346 | methylation-dependent chromatin silencin... | 10 | 10 | 10 | 0.00252 |
| GO:0048846 | axon extension involved in axon guidance | 16 | 16 | 16 | 0.00252 |
| GO:1902284 | neuron projection extension involved in ... | 16 | 16 | 16 | 0.00252 |
| GO:0075733 | intracellular transport of virus | 24 | 24 | 24 | 0.00254 |
| GO:1902583 | multi-organism intracellular transport | 24 | 24 | 24 | 0.00254 |
| GO:0060765 | regulation of androgen receptor signalin... | 16 | 16 | 16 | 0.00256 |
| GO:0007194 | negative regulation of adenylate cyclase... | 17 | 17 | 17 | 0.00257 |
| GO:0048477 | oogenesis | 33 | 33 | 33 | 0.00257 |
| GO:0032462 | regulation of protein homooligomerizatio... | 11 | 11 | 11 | 0.00258 |
| GO:2000479 | regulation of cAMP-dependent protein kin... | 12 | 12 | 12 | 0.00259 |
| GO:0015992 | proton transport | 82 | 82 | 82 | 0.00259 |
| GO:0006308 | DNA catabolic process | 21 | 21 | 21 | 0.00259 |
| GO:0001554 | luteolysis | 5 | 5 | 5 | 0.00259 |
| GO:0043374 | CD8-positive  alpha-beta T cell differen... | 6 | 6 | 6 | 0.00259 |
| GO:0003382 | epithelial cell morphogenesis | 40 | 40 | 40 | 0.00259 |
| GO:0042451 | purine nucleoside biosynthetic process | 61 | 61 | 61 | 0.00260 |
| GO:0046129 | purine ribonucleoside biosynthetic proce... | 61 | 61 | 61 | 0.00260 |
| GO:0040019 | positive regulation of embryonic develop... | 27 | 27 | 27 | 0.00260 |
| GO:0046827 | positive regulation of protein export fr... | 12 | 12 | 12 | 0.00260 |
| GO:0007530 | sex determination | 17 | 17 | 17 | 0.00261 |
| GO:0048630 | skeletal muscle tissue growth | 8 | 8 | 8 | 0.00261 |
| GO:0050926 | regulation of positive chemotaxis | 18 | 18 | 18 | 0.00262 |
| GO:0001759 | organ induction | 14 | 14 | 14 | 0.00263 |
| GO:0072215 | regulation of metanephros development | 20 | 20 | 20 | 0.00265 |
| GO:0061299 | retina vasculature morphogenesis in came... | 11 | 11 | 11 | 0.00266 |
| GO:0031061 | negative regulation of histone methylati... | 12 | 12 | 12 | 0.00267 |
| GO:0006995 | cellular response to nitrogen starvation | 14 | 14 | 14 | 0.00267 |
| GO:0043562 | cellular response to nitrogen levels | 14 | 14 | 14 | 0.00267 |
| GO:0097529 | myeloid leukocyte migration | 90 | 90 | 90 | 0.00267 |
| GO:0010996 | response to auditory stimulus | 16 | 16 | 16 | 0.00267 |
| GO:0008090 | retrograde axon cargo transport | 5 | 5 | 5 | 0.00268 |
| GO:0030812 | negative regulation of nucleotide catabo... | 12 | 12 | 12 | 0.00268 |
| GO:0050819 | negative regulation of coagulation | 33 | 33 | 33 | 0.00269 |
| GO:0001961 | positive regulation of cytokine-mediated... | 24 | 24 | 24 | 0.00270 |
| GO:0060760 | positive regulation of response to cytok... | 24 | 24 | 24 | 0.00270 |
| GO:0038083 | peptidyl-tyrosine autophosphorylation | 28 | 28 | 28 | 0.00271 |
| GO:0050930 | induction of positive chemotaxis | 9 | 9 | 9 | 0.00271 |
| GO:0006984 | ER-nucleus signaling pathway | 34 | 34 | 34 | 0.00272 |
| GO:0046543 | development of secondary female sexual c... | 7 | 7 | 7 | 0.00272 |
| GO:0031652 | positive regulation of heat generation | 7 | 7 | 7 | 0.00273 |
| GO:1903429 | regulation of cell maturation | 12 | 12 | 12 | 0.00273 |
| GO:0051057 | positive regulation of small GTPase medi... | 29 | 29 | 29 | 0.00274 |
| GO:0030071 | regulation of mitotic metaphase/anaphase... | 35 | 35 | 35 | 0.00275 |
| GO:1902099 | regulation of metaphase/anaphase transit... | 35 | 35 | 35 | 0.00275 |
| GO:0060413 | atrial septum morphogenesis | 11 | 11 | 11 | 0.00276 |
| GO:0070423 | nucleotide-binding oligomerization domai... | 26 | 26 | 26 | 0.00276 |
| GO:0007588 | excretion | 48 | 48 | 48 | 0.00277 |
| GO:0021534 | cell proliferation in hindbrain | 11 | 11 | 11 | 0.00277 |
| GO:0021924 | cell proliferation in external granule l... | 11 | 11 | 11 | 0.00277 |
| GO:0021930 | cerebellar granule cell precursor prolif... | 11 | 11 | 11 | 0.00277 |
| GO:0031649 | heat generation | 13 | 13 | 13 | 0.00278 |
| GO:0060632 | regulation of microtubule-based movement | 11 | 11 | 11 | 0.00278 |
| GO:0035520 | monoubiquitinated protein deubiquitinati... | 7 | 7 | 7 | 0.00279 |
| GO:0051571 | positive regulation of histone H3-K4 met... | 14 | 14 | 14 | 0.00279 |
| GO:0045672 | positive regulation of osteoclast differ... | 20 | 20 | 20 | 0.00281 |
| GO:0050996 | positive regulation of lipid catabolic p... | 13 | 13 | 13 | 0.00282 |
| GO:0060147 | regulation of posttranscriptional gene s... | 11 | 11 | 11 | 0.00282 |
| GO:0060966 | regulation of gene silencing by RNA | 11 | 11 | 11 | 0.00282 |
| GO:0031054 | pre-miRNA processing | 12 | 12 | 12 | 0.00284 |
| GO:1900408 | negative regulation of cellular response... | 17 | 17 | 17 | 0.00284 |
| GO:1902883 | negative regulation of response to oxida... | 17 | 17 | 17 | 0.00284 |
| GO:0030317 | sperm motility | 29 | 29 | 29 | 0.00289 |
| GO:0071346 | cellular response to interferon-gamma | 76 | 76 | 76 | 0.00289 |
| GO:0031958 | corticosteroid receptor signaling pathwa... | 17 | 17 | 17 | 0.00289 |
| GO:0021515 | cell differentiation in spinal cord | 44 | 44 | 44 | 0.00291 |
| GO:0046719 | regulation by virus of viral protein lev... | 10 | 10 | 10 | 0.00293 |
| GO:0061469 | regulation of type B pancreatic cell pro... | 7 | 7 | 7 | 0.00294 |
| GO:0045719 | negative regulation of glycogen biosynth... | 6 | 6 | 6 | 0.00294 |
| GO:0016925 | protein sumoylation | 77 | 77 | 77 | 0.00295 |
| GO:0060759 | regulation of response to cytokine stimu... | 75 | 75 | 75 | 0.00295 |
| GO:0032467 | positive regulation of cytokinesis | 20 | 20 | 20 | 0.00295 |
| GO:0055015 | ventricular cardiac muscle cell developm... | 10 | 10 | 10 | 0.00297 |
| GO:0060347 | heart trabecula formation | 10 | 10 | 10 | 0.00297 |
| GO:0009065 | glutamine family amino acid catabolic pr... | 17 | 17 | 17 | 0.00299 |
| GO:0017158 | regulation of calcium ion-dependent exoc... | 35 | 35 | 35 | 0.00299 |
| GO:0060444 | branching involved in mammary gland duct... | 21 | 21 | 21 | 0.00300 |
| GO:0071806 | protein transmembrane transport | 30 | 30 | 30 | 0.00300 |
| GO:0006818 | hydrogen transport | 84 | 84 | 84 | 0.00300 |
| GO:0071877 | regulation of adrenergic receptor signal... | 6 | 6 | 6 | 0.00301 |
| GO:0032836 | glomerular basement membrane development | 7 | 7 | 7 | 0.00301 |
| GO:0009299 | mRNA transcription | 13 | 13 | 13 | 0.00304 |
| GO:0031623 | receptor internalization | 54 | 54 | 54 | 0.00304 |
| GO:0048259 | regulation of receptor-mediated endocyto... | 54 | 54 | 54 | 0.00304 |
| GO:0048009 | insulin-like growth factor receptor sign... | 26 | 26 | 26 | 0.00307 |
| GO:1902041 | regulation of extrinsic apoptotic signal... | 33 | 33 | 33 | 0.00308 |
| GO:0072676 | lymphocyte migration | 38 | 38 | 38 | 0.00310 |
| GO:0032365 | intracellular lipid transport | 19 | 19 | 19 | 0.00311 |
| GO:0032793 | positive regulation of CREB transcriptio... | 11 | 11 | 11 | 0.00311 |
| GO:0040018 | positive regulation of multicellular org... | 24 | 24 | 24 | 0.00312 |
| GO:0042596 | fear response | 30 | 30 | 30 | 0.00315 |
| GO:0038165 | oncostatin-M-mediated signaling pathway | 4 | 4 | 4 | 0.00315 |
| GO:0070120 | ciliary neurotrophic factor-mediated sig... | 4 | 4 | 4 | 0.00315 |
| GO:0046426 | negative regulation of JAK-STAT cascade | 14 | 14 | 14 | 0.00316 |
| GO:0001967 | suckling behavior | 13 | 13 | 13 | 0.00316 |
| GO:0051043 | regulation of membrane protein ectodomai... | 14 | 14 | 14 | 0.00316 |
| GO:0002158 | osteoclast proliferation | 4 | 4 | 4 | 0.00317 |
| GO:0014015 | positive regulation of gliogenesis | 33 | 33 | 33 | 0.00318 |
| GO:0010715 | regulation of extracellular matrix disas... | 10 | 10 | 10 | 0.00319 |
| GO:2000095 | regulation of Wnt signaling pathway  pla... | 10 | 10 | 10 | 0.00319 |
| GO:0072017 | distal tubule development | 11 | 11 | 11 | 0.00320 |
| GO:0030641 | regulation of cellular pH | 45 | 45 | 45 | 0.00321 |
| GO:0090308 | regulation of methylation-dependent chro... | 4 | 4 | 4 | 0.00322 |
| GO:0023058 | adaptation of signaling pathway | 13 | 13 | 13 | 0.00324 |
| GO:0050861 | positive regulation of B cell receptor s... | 4 | 4 | 4 | 0.00325 |
| GO:0032438 | melanosome organization | 13 | 13 | 13 | 0.00327 |
| GO:0048753 | pigment granule organization | 13 | 13 | 13 | 0.00327 |
| GO:0006635 | fatty acid beta-oxidation | 44 | 44 | 44 | 0.00329 |
| GO:0044766 | multi-organism transport | 27 | 27 | 27 | 0.00332 |
| GO:0046794 | transport of virus | 27 | 27 | 27 | 0.00332 |
| GO:1902579 | multi-organism localization | 27 | 27 | 27 | 0.00332 |
| GO:0010763 | positive regulation of fibroblast migrat... | 5 | 5 | 5 | 0.00332 |
| GO:0019674 | NAD metabolic process | 33 | 33 | 33 | 0.00333 |
| GO:0043407 | negative regulation of MAP kinase activi... | 52 | 52 | 52 | 0.00334 |
| GO:0051952 | regulation of amine transport | 46 | 46 | 46 | 0.00334 |
| GO:1902306 | negative regulation of sodium ion transm... | 7 | 7 | 7 | 0.00334 |
| GO:2000650 | negative regulation of sodium ion transm... | 7 | 7 | 7 | 0.00334 |
| GO:0016072 | rRNA metabolic process | 70 | 70 | 70 | 0.00335 |
| GO:0071621 | granulocyte chemotaxis | 58 | 58 | 58 | 0.00336 |
| GO:0046364 | monosaccharide biosynthetic process | 61 | 61 | 61 | 0.00336 |
| GO:0042310 | vasoconstriction | 50 | 50 | 50 | 0.00337 |
| GO:0032026 | response to magnesium ion | 11 | 11 | 11 | 0.00337 |
| GO:0030890 | positive regulation of B cell proliferat... | 26 | 26 | 26 | 0.00338 |
| GO:0042089 | cytokine biosynthetic process | 64 | 64 | 64 | 0.00338 |
| GO:0071371 | cellular response to gonadotropin stimul... | 15 | 15 | 15 | 0.00338 |
| GO:0046716 | muscle cell cellular homeostasis | 15 | 15 | 15 | 0.00340 |
| GO:0048873 | homeostasis of number of cells within a ... | 16 | 16 | 16 | 0.00340 |
| GO:0000132 | establishment of mitotic spindle orienta... | 16 | 16 | 16 | 0.00341 |
| GO:0060706 | cell differentiation involved in embryon... | 21 | 21 | 21 | 0.00341 |
| GO:0042993 | positive regulation of transcription fac... | 32 | 32 | 32 | 0.00342 |
| GO:0050830 | defense response to Gram-positive bacter... | 31 | 31 | 31 | 0.00343 |
| GO:0098751 | bone cell development | 22 | 22 | 22 | 0.00345 |
| GO:0045671 | negative regulation of osteoclast differ... | 15 | 15 | 15 | 0.00345 |
| GO:0043516 | regulation of DNA damage response  signa... | 20 | 20 | 20 | 0.00347 |
| GO:0036342 | post-anal tail morphogenesis | 15 | 15 | 15 | 0.00349 |
| GO:0060766 | negative regulation of androgen receptor... | 10 | 10 | 10 | 0.00350 |
| GO:0043124 | negative regulation of I-kappaB kinase/N... | 33 | 33 | 33 | 0.00350 |
| GO:0010829 | negative regulation of glucose transport | 13 | 13 | 13 | 0.00351 |
| GO:0007638 | mechanosensory behavior | 10 | 10 | 10 | 0.00351 |
| GO:1900271 | regulation of long-term synaptic potenti... | 10 | 10 | 10 | 0.00351 |
| GO:0034315 | regulation of Arp2/3 complex-mediated ac... | 10 | 10 | 10 | 0.00354 |
| GO:0045655 | regulation of monocyte differentiation | 13 | 13 | 13 | 0.00354 |
| GO:0030947 | regulation of vascular endothelial growt... | 22 | 22 | 22 | 0.00355 |
| GO:0051646 | mitochondrion localization | 20 | 20 | 20 | 0.00357 |
| GO:0033280 | response to vitamin D | 23 | 23 | 23 | 0.00357 |
| GO:0072413 | signal transduction involved in mitotic ... | 39 | 39 | 39 | 0.00359 |
| GO:1902402 | signal transduction involved in mitotic ... | 39 | 39 | 39 | 0.00359 |
| GO:1902403 | signal transduction involved in mitotic ... | 39 | 39 | 39 | 0.00359 |
| GO:0032309 | icosanoid secretion | 14 | 14 | 14 | 0.00360 |
| GO:0007200 | phospholipase C-activating G-protein cou... | 47 | 47 | 47 | 0.00361 |
| GO:0035988 | chondrocyte proliferation | 13 | 13 | 13 | 0.00365 |
| GO:2000628 | regulation of miRNA metabolic process | 7 | 7 | 7 | 0.00365 |
| GO:1901532 | regulation of hematopoietic progenitor c... | 25 | 25 | 25 | 0.00366 |
| GO:0021854 | hypothalamus development | 14 | 14 | 14 | 0.00366 |
| GO:0031952 | regulation of protein autophosphorylatio... | 23 | 23 | 23 | 0.00366 |
| GO:0030809 | negative regulation of nucleotide biosyn... | 26 | 26 | 26 | 0.00367 |
| GO:1900372 | negative regulation of purine nucleotide... | 26 | 26 | 26 | 0.00367 |
| GO:0032897 | negative regulation of viral transcripti... | 16 | 16 | 16 | 0.00368 |
| GO:0045779 | negative regulation of bone resorption | 7 | 7 | 7 | 0.00368 |
| GO:0042754 | negative regulation of circadian rhythm | 13 | 13 | 13 | 0.00369 |
| GO:0071436 | sodium ion export | 9 | 9 | 9 | 0.00370 |
| GO:0032926 | negative regulation of activin receptor ... | 7 | 7 | 7 | 0.00370 |
| GO:0060300 | regulation of cytokine activity | 4 | 4 | 4 | 0.00370 |
| GO:0022904 | respiratory electron transport chain | 53 | 53 | 53 | 0.00370 |
| GO:0042102 | positive regulation of T cell proliferat... | 41 | 41 | 41 | 0.00370 |
| GO:1903201 | regulation of oxidative stress-induced c... | 23 | 23 | 23 | 0.00371 |
| GO:0060142 | regulation of syncytium formation by pla... | 19 | 19 | 19 | 0.00374 |
| GO:0060716 | labyrinthine layer blood vessel developm... | 11 | 11 | 11 | 0.00374 |
| GO:0060742 | epithelial cell differentiation involved... | 7 | 7 | 7 | 0.00375 |
| GO:0060526 | prostate glandular acinus morphogenesis | 7 | 7 | 7 | 0.00375 |
| GO:0060527 | prostate epithelial cord arborization in... | 7 | 7 | 7 | 0.00375 |
| GO:0050951 | sensory perception of temperature stimul... | 14 | 14 | 14 | 0.00376 |
| GO:0021957 | corticospinal tract morphogenesis | 5 | 5 | 5 | 0.00378 |
| GO:0014072 | response to isoquinoline alkaloid | 20 | 20 | 20 | 0.00378 |
| GO:0002675 | positive regulation of acute inflammator... | 16 | 16 | 16 | 0.00380 |
| GO:0007606 | sensory perception of chemical stimulus | 52 | 52 | 52 | 0.00380 |
| GO:0045745 | positive regulation of G-protein coupled... | 12 | 12 | 12 | 0.00383 |
| GO:0002793 | positive regulation of peptide secretion | 56 | 56 | 56 | 0.00385 |
| GO:0030195 | negative regulation of blood coagulation | 32 | 32 | 32 | 0.00386 |
| GO:1900047 | negative regulation of hemostasis | 32 | 32 | 32 | 0.00386 |
| GO:0045717 | negative regulation of fatty acid biosyn... | 10 | 10 | 10 | 0.00386 |
| GO:0006289 | nucleotide-excision repair | 48 | 48 | 48 | 0.00388 |
| GO:0051353 | positive regulation of oxidoreductase ac... | 30 | 30 | 30 | 0.00388 |
| GO:1901201 | regulation of extracellular matrix assem... | 8 | 8 | 8 | 0.00389 |
| GO:0060440 | trachea formation | 5 | 5 | 5 | 0.00391 |
| GO:1901620 | regulation of smoothened signaling pathw... | 6 | 6 | 6 | 0.00392 |
| GO:0032000 | positive regulation of fatty acid beta-o... | 7 | 7 | 7 | 0.00393 |
| GO:0051574 | positive regulation of histone H3-K9 met... | 5 | 5 | 5 | 0.00393 |
| GO:0003203 | endocardial cushion morphogenesis | 14 | 14 | 14 | 0.00393 |
| GO:0007175 | negative regulation of epidermal growth ... | 7 | 7 | 7 | 0.00394 |
| GO:0044246 | regulation of multicellular organismal m... | 21 | 21 | 21 | 0.00394 |
| GO:0050650 | chondroitin sulfate proteoglycan biosynt... | 23 | 23 | 23 | 0.00395 |
| GO:0010992 | ubiquitin homeostasis | 5 | 5 | 5 | 0.00395 |
| GO:1902774 | late endosome to lysosome transport | 5 | 5 | 5 | 0.00397 |
| GO:0046069 | cGMP catabolic process | 5 | 5 | 5 | 0.00400 |
| GO:0061099 | negative regulation of protein tyrosine ... | 15 | 15 | 15 | 0.00401 |
| GO:0006680 | glucosylceramide catabolic process | 3 | 3 | 3 | 0.00401 |
| GO:0060750 | epithelial cell proliferation involved i... | 5 | 5 | 5 | 0.00401 |
| GO:0060751 | branch elongation involved in mammary gl... | 5 | 5 | 5 | 0.00401 |
| GO:0008105 | asymmetric protein localization | 19 | 19 | 19 | 0.00403 |
| GO:0002719 | negative regulation of cytokine producti... | 8 | 8 | 8 | 0.00406 |
| GO:0002714 | positive regulation of B cell mediated i... | 13 | 13 | 13 | 0.00407 |
| GO:0002891 | positive regulation of immunoglobulin me... | 13 | 13 | 13 | 0.00407 |
| GO:0006959 | humoral immune response | 80 | 80 | 80 | 0.00407 |
| GO:0051797 | regulation of hair follicle development | 9 | 9 | 9 | 0.00407 |
| GO:0014038 | regulation of Schwann cell differentiati... | 3 | 3 | 3 | 0.00408 |
| GO:0030950 | establishment or maintenance of actin cy... | 3 | 3 | 3 | 0.00409 |
| GO:0015701 | bicarbonate transport | 30 | 30 | 30 | 0.00409 |
| GO:0006575 | cellular modified amino acid metabolic p... | 127 | 127 | 127 | 0.00409 |
| GO:0006458 | 'de novo' protein folding | 31 | 31 | 31 | 0.00410 |
| GO:1902229 | regulation of intrinsic apoptotic signal... | 27 | 27 | 27 | 0.00410 |
| GO:0071435 | potassium ion export | 8 | 8 | 8 | 0.00411 |
| GO:0044154 | histone H3-K14 acetylation | 7 | 7 | 7 | 0.00412 |
| GO:0071480 | cellular response to gamma radiation | 11 | 11 | 11 | 0.00413 |
| GO:0048536 | spleen development | 29 | 29 | 29 | 0.00415 |
| GO:0007100 | mitotic centrosome separation | 7 | 7 | 7 | 0.00415 |
| GO:0051299 | centrosome separation | 7 | 7 | 7 | 0.00415 |
| GO:0006695 | cholesterol biosynthetic process | 30 | 30 | 30 | 0.00416 |
| GO:1902653 | secondary alcohol biosynthetic process | 30 | 30 | 30 | 0.00416 |
| GO:1903146 | regulation of mitophagy | 27 | 27 | 27 | 0.00416 |
| GO:0048643 | positive regulation of skeletal muscle t... | 19 | 19 | 19 | 0.00417 |
| GO:0060534 | trachea cartilage development | 7 | 7 | 7 | 0.00420 |
| GO:0090201 | negative regulation of release of cytoch... | 11 | 11 | 11 | 0.00422 |
| GO:0071600 | otic vesicle morphogenesis | 8 | 8 | 8 | 0.00423 |
| GO:0021891 | olfactory bulb interneuron development | 7 | 7 | 7 | 0.00423 |
| GO:0048569 | post-embryonic organ development | 13 | 13 | 13 | 0.00424 |
| GO:0060795 | cell fate commitment involved in formati... | 26 | 26 | 26 | 0.00425 |
| GO:2000831 | regulation of steroid hormone secretion | 9 | 9 | 9 | 0.00425 |
| GO:0021785 | branchiomotor neuron axon guidance | 5 | 5 | 5 | 0.00427 |
| GO:0043552 | positive regulation of phosphatidylinosi... | 24 | 24 | 24 | 0.00428 |
| GO:0048172 | regulation of short-term neuronal synapt... | 11 | 11 | 11 | 0.00429 |
| GO:2000279 | negative regulation of DNA biosynthetic ... | 12 | 12 | 12 | 0.00430 |
| GO:0060633 | negative regulation of transcription ini... | 4 | 4 | 4 | 0.00430 |
| GO:2000143 | negative regulation of DNA-templated tra... | 4 | 4 | 4 | 0.00430 |
| GO:0006991 | response to sterol depletion | 9 | 9 | 9 | 0.00433 |
| GO:0032933 | SREBP signaling pathway | 9 | 9 | 9 | 0.00433 |
| GO:0071501 | cellular response to sterol depletion | 9 | 9 | 9 | 0.00433 |
| GO:0051490 | negative regulation of filopodium assemb... | 3 | 3 | 3 | 0.00433 |
| GO:0071157 | negative regulation of cell cycle arrest | 8 | 8 | 8 | 0.00433 |
| GO:0015732 | prostaglandin transport | 8 | 8 | 8 | 0.00435 |
| GO:0046329 | negative regulation of JNK cascade | 25 | 25 | 25 | 0.00436 |
| GO:0043462 | regulation of ATPase activity | 31 | 31 | 31 | 0.00439 |
| GO:0031017 | exocrine pancreas development | 8 | 8 | 8 | 0.00440 |
| GO:1902254 | negative regulation of intrinsic apoptot... | 10 | 10 | 10 | 0.00440 |
| GO:0043268 | positive regulation of potassium ion tra... | 30 | 30 | 30 | 0.00440 |
| GO:0042506 | tyrosine phosphorylation of Stat5 protei... | 17 | 17 | 17 | 0.00443 |
| GO:0071498 | cellular response to fluid shear stress | 19 | 19 | 19 | 0.00444 |
| GO:0007029 | endoplasmic reticulum organization | 32 | 32 | 32 | 0.00445 |
| GO:1901796 | regulation of signal transduction by p53... | 35 | 35 | 35 | 0.00446 |
| GO:0001504 | neurotransmitter uptake | 20 | 20 | 20 | 0.00447 |
| GO:0071391 | cellular response to estrogen stimulus | 21 | 21 | 21 | 0.00447 |
| GO:0006414 | translational elongation | 68 | 68 | 68 | 0.00449 |
| GO:0016578 | histone deubiquitination | 18 | 18 | 18 | 0.00450 |
| GO:0086012 | membrane depolarization during cardiac m... | 11 | 11 | 11 | 0.00451 |
| GO:0007440 | foregut morphogenesis | 9 | 9 | 9 | 0.00451 |
| GO:0006826 | iron ion transport | 37 | 37 | 37 | 0.00452 |
| GO:0048642 | negative regulation of skeletal muscle t... | 11 | 11 | 11 | 0.00453 |
| GO:0071679 | commissural neuron axon guidance | 5 | 5 | 5 | 0.00454 |
| GO:0021520 | spinal cord motor neuron cell fate speci... | 12 | 12 | 12 | 0.00454 |
| GO:0007031 | peroxisome organization | 25 | 25 | 25 | 0.00455 |
| GO:0007062 | sister chromatid cohesion | 32 | 32 | 32 | 0.00456 |
| GO:0045061 | thymic T cell selection | 16 | 16 | 16 | 0.00457 |
| GO:0002676 | regulation of chronic inflammatory respo... | 6 | 6 | 6 | 0.00458 |
| GO:0060430 | lung saccule development | 6 | 6 | 6 | 0.00459 |
| GO:0002544 | chronic inflammatory response | 15 | 15 | 15 | 0.00459 |
| GO:0051044 | positive regulation of membrane protein ... | 9 | 9 | 9 | 0.00460 |
| GO:2000096 | positive regulation of Wnt signaling pat... | 5 | 5 | 5 | 0.00462 |
| GO:0009629 | response to gravity | 6 | 6 | 6 | 0.00463 |
| GO:0051453 | regulation of intracellular pH | 43 | 43 | 43 | 0.00463 |
| GO:0006636 | unsaturated fatty acid biosynthetic proc... | 33 | 33 | 33 | 0.00464 |
| GO:0032905 | transforming growth factor beta1 product... | 6 | 6 | 6 | 0.00468 |
| GO:0032908 | regulation of transforming growth factor... | 6 | 6 | 6 | 0.00468 |
| GO:0046033 | AMP metabolic process | 14 | 14 | 14 | 0.00468 |
| GO:0019060 | intracellular transport of viral protein... | 4 | 4 | 4 | 0.00471 |
| GO:0030581 | symbiont intracellular protein transport... | 4 | 4 | 4 | 0.00471 |
| GO:0051708 | intracellular protein transport in other... | 4 | 4 | 4 | 0.00471 |
| GO:0015696 | ammonium transport | 59 | 59 | 59 | 0.00472 |
| GO:0014048 | regulation of glutamate secretion | 10 | 10 | 10 | 0.00472 |
| GO:0016188 | synaptic vesicle maturation | 9 | 9 | 9 | 0.00473 |
| GO:0072044 | collecting duct development | 10 | 10 | 10 | 0.00474 |
| GO:0008202 | steroid metabolic process | 167 | 167 | 167 | 0.00474 |
| GO:0044346 | fibroblast apoptotic process | 16 | 16 | 16 | 0.00476 |
| GO:0021511 | spinal cord patterning | 21 | 21 | 21 | 0.00478 |
| GO:0070257 | positive regulation of mucus secretion | 4 | 4 | 4 | 0.00479 |
| GO:0035773 | insulin secretion involved in cellular r... | 40 | 40 | 40 | 0.00480 |
| GO:0000578 | embryonic axis specification | 28 | 28 | 28 | 0.00481 |
| GO:0060027 | convergent extension involved in gastrul... | 4 | 4 | 4 | 0.00481 |
| GO:0050775 | positive regulation of dendrite morphoge... | 21 | 21 | 21 | 0.00482 |
| GO:0021535 | cell migration in hindbrain | 12 | 12 | 12 | 0.00483 |
| GO:0060677 | ureteric bud elongation | 6 | 6 | 6 | 0.00483 |
| GO:0060768 | regulation of epithelial cell proliferat... | 5 | 5 | 5 | 0.00484 |
| GO:0032770 | positive regulation of monooxygenase act... | 19 | 19 | 19 | 0.00485 |
| GO:1900038 | negative regulation of cellular response... | 3 | 3 | 3 | 0.00486 |
| GO:0044340 | canonical Wnt signaling pathway involved... | 5 | 5 | 5 | 0.00486 |
| GO:0045616 | regulation of keratinocyte differentiati... | 17 | 17 | 17 | 0.00487 |
| GO:2000322 | regulation of glucocorticoid receptor si... | 9 | 9 | 9 | 0.00487 |
| GO:0061077 | chaperone-mediated protein folding | 36 | 36 | 36 | 0.00490 |
| GO:0021694 | cerebellar Purkinje cell layer formation | 12 | 12 | 12 | 0.00491 |
| GO:0021702 | cerebellar Purkinje cell differentiation | 12 | 12 | 12 | 0.00491 |
| GO:0042107 | cytokine metabolic process | 65 | 65 | 65 | 0.00492 |
| GO:0043217 | myelin maintenance | 9 | 9 | 9 | 0.00492 |
| GO:0051953 | negative regulation of amine transport | 18 | 18 | 18 | 0.00493 |
| GO:0048715 | negative regulation of oligodendrocyte d... | 11 | 11 | 11 | 0.00497 |
| GO:0048387 | negative regulation of retinoic acid rec... | 9 | 9 | 9 | 0.00497 |
| GO:2001222 | regulation of neuron migration | 20 | 20 | 20 | 0.00497 |
| GO:0090114 | COPII-coated vesicle budding | 17 | 17 | 17 | 0.00499 |
| GO:0045161 | neuronal ion channel clustering | 12 | 12 | 12 | 0.00500 |
| GO:0090005 | negative regulation of establishment of ... | 11 | 11 | 11 | 0.00500 |
| GO:0045792 | negative regulation of cell size | 6 | 6 | 6 | 0.00500 |
| GO:0016601 | Rac protein signal transduction | 26 | 26 | 26 | 0.00501 |
| GO:0038066 | p38MAPK cascade | 20 | 20 | 20 | 0.00502 |
| GO:0042711 | maternal behavior | 8 | 8 | 8 | 0.00503 |
| GO:0060746 | parental behavior | 8 | 8 | 8 | 0.00503 |
| GO:0071321 | cellular response to cGMP | 6 | 6 | 6 | 0.00503 |
| GO:0014816 | skeletal muscle satellite cell different... | 7 | 7 | 7 | 0.00504 |
| GO:0043489 | RNA stabilization | 27 | 27 | 27 | 0.00505 |
| GO:0048255 | mRNA stabilization | 27 | 27 | 27 | 0.00505 |
| GO:0007019 | microtubule depolymerization | 25 | 25 | 25 | 0.00505 |
| GO:0035196 | production of miRNAs involved in gene si... | 18 | 18 | 18 | 0.00505 |
| GO:0061098 | positive regulation of protein tyrosine ... | 25 | 25 | 25 | 0.00507 |
| GO:0003338 | metanephros morphogenesis | 26 | 26 | 26 | 0.00507 |
| GO:0007183 | SMAD protein complex assembly | 10 | 10 | 10 | 0.00507 |
| GO:1903055 | positive regulation of extracellular mat... | 10 | 10 | 10 | 0.00507 |
| GO:0002821 | positive regulation of adaptive immune r... | 34 | 34 | 34 | 0.00510 |
| GO:0071875 | adrenergic receptor signaling pathway | 18 | 18 | 18 | 0.00511 |
| GO:0008063 | Toll signaling pathway | 4 | 4 | 4 | 0.00513 |
| GO:0045923 | positive regulation of fatty acid metabo... | 17 | 17 | 17 | 0.00513 |
| GO:0060572 | morphogenesis of an epithelial bud | 13 | 13 | 13 | 0.00513 |
| GO:1902547 | regulation of cellular response to vascu... | 9 | 9 | 9 | 0.00514 |
| GO:0060964 | regulation of gene silencing by miRNA | 8 | 8 | 8 | 0.00520 |
| GO:0033564 | anterior/posterior axon guidance | 5 | 5 | 5 | 0.00520 |
| GO:0038028 | insulin receptor signaling pathway via p... | 3 | 3 | 3 | 0.00521 |
| GO:2000467 | positive regulation of glycogen (starch)... | 3 | 3 | 3 | 0.00521 |
| GO:0044273 | sulfur compound catabolic process | 26 | 26 | 26 | 0.00521 |
| GO:0043584 | nose development | 11 | 11 | 11 | 0.00521 |
| GO:0021602 | cranial nerve morphogenesis | 17 | 17 | 17 | 0.00522 |
| GO:0051955 | regulation of amino acid transport | 16 | 16 | 16 | 0.00525 |
| GO:0097106 | postsynaptic density organization | 4 | 4 | 4 | 0.00526 |
| GO:0097107 | postsynaptic density assembly | 4 | 4 | 4 | 0.00526 |
| GO:0097118 | neuroligin clustering involved in postsy... | 4 | 4 | 4 | 0.00526 |
| GO:0042483 | negative regulation of odontogenesis | 5 | 5 | 5 | 0.00529 |
| GO:0060235 | lens induction in camera-type eye | 3 | 3 | 3 | 0.00532 |
| GO:0045647 | negative regulation of erythrocyte diffe... | 5 | 5 | 5 | 0.00533 |
| GO:0001895 | retina homeostasis | 41 | 41 | 41 | 0.00533 |
| GO:0097530 | granulocyte migration | 64 | 64 | 64 | 0.00533 |
| GO:0003415 | chondrocyte hypertrophy | 4 | 4 | 4 | 0.00533 |
| GO:1990086 | lens fiber cell apoptotic process | 4 | 4 | 4 | 0.00533 |
| GO:0036475 | neuron death in response to oxidative st... | 6 | 6 | 6 | 0.00534 |
| GO:1903203 | regulation of oxidative stress-induced n... | 6 | 6 | 6 | 0.00534 |
| GO:0060788 | ectodermal placode formation | 13 | 13 | 13 | 0.00534 |
| GO:0071696 | ectodermal placode development | 13 | 13 | 13 | 0.00534 |
| GO:0071697 | ectodermal placode morphogenesis | 13 | 13 | 13 | 0.00534 |
| GO:0060314 | regulation of ryanodine-sensitive calciu... | 21 | 21 | 21 | 0.00535 |
| GO:0061028 | establishment of endothelial barrier | 28 | 28 | 28 | 0.00537 |
| GO:0060346 | bone trabecula formation | 8 | 8 | 8 | 0.00537 |
| GO:0060644 | mammary gland epithelial cell differenti... | 12 | 12 | 12 | 0.00538 |
| GO:1903428 | positive regulation of reactive oxygen s... | 30 | 30 | 30 | 0.00541 |
| GO:0072025 | distal convoluted tubule development | 4 | 4 | 4 | 0.00543 |
| GO:0072221 | metanephric distal convoluted tubule dev... | 4 | 4 | 4 | 0.00543 |
| GO:0035904 | aorta development | 20 | 20 | 20 | 0.00544 |
| GO:0051055 | negative regulation of lipid biosyntheti... | 29 | 29 | 29 | 0.00544 |
| GO:1903392 | negative regulation of adherens junction... | 13 | 13 | 13 | 0.00544 |
| GO:0060317 | cardiac epithelial to mesenchymal transi... | 18 | 18 | 18 | 0.00544 |
| GO:0044332 | Wnt signaling pathway involved in dorsal... | 7 | 7 | 7 | 0.00547 |
| GO:0035338 | long-chain fatty-acyl-CoA biosynthetic p... | 14 | 14 | 14 | 0.00547 |
| GO:0002439 | chronic inflammatory response to antigen... | 5 | 5 | 5 | 0.00547 |
| GO:0044060 | regulation of endocrine process | 22 | 22 | 22 | 0.00552 |
| GO:2000114 | regulation of establishment of cell pola... | 15 | 15 | 15 | 0.00554 |
| GO:0050908 | detection of light stimulus involved in ... | 12 | 12 | 12 | 0.00556 |
| GO:0050962 | detection of light stimulus involved in ... | 12 | 12 | 12 | 0.00556 |
| GO:2000001 | regulation of DNA damage checkpoint | 10 | 10 | 10 | 0.00557 |
| GO:0031280 | negative regulation of cyclase activity | 18 | 18 | 18 | 0.00558 |
| GO:0006904 | vesicle docking involved in exocytosis | 32 | 32 | 32 | 0.00559 |
| GO:0000724 | double-strand break repair via homologou... | 45 | 45 | 45 | 0.00561 |
| GO:0000725 | recombinational repair | 45 | 45 | 45 | 0.00561 |
| GO:1900078 | positive regulation of cellular response... | 13 | 13 | 13 | 0.00564 |
| GO:0002437 | inflammatory response to antigenic stimu... | 23 | 23 | 23 | 0.00566 |
| GO:0003214 | cardiac left ventricle morphogenesis | 11 | 11 | 11 | 0.00566 |
| GO:0060707 | trophoblast giant cell differentiation | 11 | 11 | 11 | 0.00569 |
| GO:0033148 | positive regulation of intracellular est... | 7 | 7 | 7 | 0.00573 |
| GO:0009127 | purine nucleoside monophosphate biosynth... | 41 | 41 | 41 | 0.00574 |
| GO:0009168 | purine ribonucleoside monophosphate bios... | 41 | 41 | 41 | 0.00574 |
| GO:1901629 | regulation of presynaptic membrane organ... | 7 | 7 | 7 | 0.00574 |
| GO:0031663 | lipopolysaccharide-mediated signaling pa... | 30 | 30 | 30 | 0.00577 |
| GO:0097502 | mannosylation | 16 | 16 | 16 | 0.00577 |
| GO:0045540 | regulation of cholesterol biosynthetic p... | 10 | 10 | 10 | 0.00578 |
| GO:0090630 | activation of GTPase activity | 30 | 30 | 30 | 0.00580 |
| GO:0042953 | lipoprotein transport | 8 | 8 | 8 | 0.00580 |
| GO:0044872 | lipoprotein localization | 8 | 8 | 8 | 0.00580 |
| GO:0045714 | regulation of low-density lipoprotein pa... | 8 | 8 | 8 | 0.00581 |
| GO:0043044 | ATP-dependent chromatin remodeling | 47 | 47 | 47 | 0.00582 |
| GO:0010453 | regulation of cell fate commitment | 21 | 21 | 21 | 0.00585 |
| GO:0010560 | positive regulation of glycoprotein bios... | 15 | 15 | 15 | 0.00586 |
| GO:0000715 | nucleotide-excision repair  DNA damage r... | 5 | 5 | 5 | 0.00586 |
| GO:1903825 | organic acid transmembrane transport | 67 | 67 | 67 | 0.00587 |
| GO:0070934 | CRD-mediated mRNA stabilization | 5 | 5 | 5 | 0.00587 |
| GO:0061154 | endothelial tube morphogenesis | 8 | 8 | 8 | 0.00587 |
| GO:0032635 | interleukin-6 production | 60 | 60 | 60 | 0.00589 |
| GO:0022900 | electron transport chain | 54 | 54 | 54 | 0.00591 |
| GO:0060318 | definitive erythrocyte differentiation | 5 | 5 | 5 | 0.00591 |
| GO:2000074 | regulation of type B pancreatic cell dev... | 8 | 8 | 8 | 0.00593 |
| GO:1901380 | negative regulation of potassium ion tra... | 14 | 14 | 14 | 0.00594 |
| GO:0051297 | centrosome organization | 65 | 65 | 65 | 0.00594 |
| GO:0055081 | anion homeostasis | 40 | 40 | 40 | 0.00594 |
| GO:1903265 | positive regulation of tumor necrosis fa... | 4 | 4 | 4 | 0.00594 |
| GO:0010888 | negative regulation of lipid storage | 13 | 13 | 13 | 0.00595 |
| GO:0048333 | mesodermal cell differentiation | 23 | 23 | 23 | 0.00595 |
| GO:0045736 | negative regulation of cyclin-dependent ... | 26 | 26 | 26 | 0.00596 |
| GO:0071361 | cellular response to ethanol | 8 | 8 | 8 | 0.00597 |
| GO:0071907 | determination of digestive tract left/ri... | 4 | 4 | 4 | 0.00598 |
| GO:0033151 | V(D)J recombination | 10 | 10 | 10 | 0.00600 |
| GO:0042116 | macrophage activation | 23 | 23 | 23 | 0.00601 |
| GO:0070977 | bone maturation | 15 | 15 | 15 | 0.00602 |
| GO:0031077 | post-embryonic camera-type eye developme... | 7 | 7 | 7 | 0.00605 |
| GO:2000810 | regulation of bicellular tight junction ... | 10 | 10 | 10 | 0.00610 |
| GO:0019218 | regulation of steroid metabolic process | 51 | 51 | 51 | 0.00615 |
| GO:2001275 | positive regulation of glucose import in... | 9 | 9 | 9 | 0.00617 |
| GO:0045922 | negative regulation of fatty acid metabo... | 12 | 12 | 12 | 0.00617 |
| GO:0061323 | cell proliferation involved in heart mor... | 13 | 13 | 13 | 0.00621 |
| GO:2000188 | regulation of cholesterol homeostasis | 9 | 9 | 9 | 0.00622 |
| GO:0034349 | glial cell apoptotic process | 15 | 15 | 15 | 0.00624 |
| GO:0040001 | establishment of mitotic spindle localiz... | 19 | 19 | 19 | 0.00625 |
| GO:0036301 | macrophage colony-stimulating factor pro... | 3 | 3 | 3 | 0.00626 |
| GO:1901256 | regulation of macrophage colony-stimulat... | 3 | 3 | 3 | 0.00626 |
| GO:0001839 | neural plate morphogenesis | 6 | 6 | 6 | 0.00628 |
| GO:0002685 | regulation of leukocyte migration | 83 | 83 | 83 | 0.00628 |
| GO:0042522 | regulation of tyrosine phosphorylation o... | 15 | 15 | 15 | 0.00630 |
| GO:0050982 | detection of mechanical stimulus | 31 | 31 | 31 | 0.00630 |
| GO:0007618 | mating | 26 | 26 | 26 | 0.00631 |
| GO:0042345 | regulation of NF-kappaB import into nucl... | 27 | 27 | 27 | 0.00631 |
| GO:0042348 | NF-kappaB import into nucleus | 27 | 27 | 27 | 0.00631 |
| GO:0048245 | eosinophil chemotaxis | 9 | 9 | 9 | 0.00632 |
| GO:0002069 | columnar/cuboidal epithelial cell matura... | 6 | 6 | 6 | 0.00634 |
| GO:0060136 | embryonic process involved in female pre... | 4 | 4 | 4 | 0.00635 |
| GO:0030851 | granulocyte differentiation | 23 | 23 | 23 | 0.00635 |
| GO:0034350 | regulation of glial cell apoptotic proce... | 7 | 7 | 7 | 0.00635 |
| GO:0034351 | negative regulation of glial cell apopto... | 7 | 7 | 7 | 0.00635 |
| GO:0060916 | mesenchymal cell proliferation involved ... | 3 | 3 | 3 | 0.00641 |
| GO:0014841 | skeletal muscle satellite cell prolifera... | 8 | 8 | 8 | 0.00641 |
| GO:2000347 | positive regulation of hepatocyte prolif... | 5 | 5 | 5 | 0.00643 |
| GO:0002691 | regulation of cellular extravasation | 15 | 15 | 15 | 0.00643 |
| GO:0051151 | negative regulation of smooth muscle cel... | 6 | 6 | 6 | 0.00644 |
| GO:0001578 | microtubule bundle formation | 33 | 33 | 33 | 0.00645 |
| GO:0032024 | positive regulation of insulin secretion | 42 | 42 | 42 | 0.00646 |
| GO:0090083 | regulation of inclusion body assembly | 14 | 14 | 14 | 0.00646 |
| GO:0071107 | response to parathyroid hormone | 10 | 10 | 10 | 0.00647 |
| GO:0007256 | activation of JNKK activity | 8 | 8 | 8 | 0.00648 |
| GO:0048012 | hepatocyte growth factor receptor signal... | 10 | 10 | 10 | 0.00648 |
| GO:0000184 | nuclear-transcribed mRNA catabolic proce... | 40 | 40 | 40 | 0.00650 |
| GO:0048339 | paraxial mesoderm development | 14 | 14 | 14 | 0.00651 |
| GO:0030916 | otic vesicle formation | 5 | 5 | 5 | 0.00651 |
| GO:0051000 | positive regulation of nitric-oxide synt... | 15 | 15 | 15 | 0.00652 |
| GO:0002678 | positive regulation of chronic inflammat... | 3 | 3 | 3 | 0.00654 |
| GO:0097154 | GABAergic neuron differentiation | 12 | 12 | 12 | 0.00654 |
| GO:0030157 | pancreatic juice secretion | 6 | 6 | 6 | 0.00656 |
| GO:0002704 | negative regulation of leukocyte mediate... | 21 | 21 | 21 | 0.00658 |
| GO:1902001 | fatty acid transmembrane transport | 11 | 11 | 11 | 0.00662 |
| GO:0016246 | RNA interference | 10 | 10 | 10 | 0.00662 |
| GO:0003062 | regulation of heart rate by chemical sig... | 6 | 6 | 6 | 0.00662 |
| GO:0040037 | negative regulation of fibroblast growth... | 10 | 10 | 10 | 0.00664 |
| GO:0060179 | male mating behavior | 3 | 3 | 3 | 0.00666 |
| GO:0051532 | regulation of NFAT protein import into n... | 13 | 13 | 13 | 0.00668 |
| GO:0010421 | hydrogen peroxide-mediated programmed ce... | 3 | 3 | 3 | 0.00668 |
| GO:0097468 | programmed cell death in response to rea... | 3 | 3 | 3 | 0.00668 |
| GO:0030857 | negative regulation of epithelial cell d... | 29 | 29 | 29 | 0.00669 |
| GO:0003417 | growth plate cartilage development | 12 | 12 | 12 | 0.00672 |
| GO:0003298 | physiological muscle hypertrophy | 9 | 9 | 9 | 0.00672 |
| GO:0003301 | physiological cardiac muscle hypertrophy | 9 | 9 | 9 | 0.00672 |
| GO:0061049 | cell growth involved in cardiac muscle c... | 9 | 9 | 9 | 0.00672 |
| GO:0048934 | peripheral nervous system neuron differe... | 12 | 12 | 12 | 0.00672 |
| GO:0048935 | peripheral nervous system neuron develop... | 12 | 12 | 12 | 0.00672 |
| GO:0014067 | negative regulation of phosphatidylinosi... | 10 | 10 | 10 | 0.00674 |
| GO:0051938 | L-glutamate import | 8 | 8 | 8 | 0.00675 |
| GO:0002327 | immature B cell differentiation | 5 | 5 | 5 | 0.00676 |
| GO:1902882 | regulation of response to oxidative stre... | 34 | 34 | 34 | 0.00676 |
| GO:0060307 | regulation of ventricular cardiac muscle... | 13 | 13 | 13 | 0.00677 |
| GO:0045023 | G0 to G1 transition | 7 | 7 | 7 | 0.00679 |
| GO:0006474 | N-terminal protein amino acid acetylatio... | 12 | 12 | 12 | 0.00680 |
| GO:1902475 | L-alpha-amino acid transmembrane transpo... | 24 | 24 | 24 | 0.00682 |
| GO:0060013 | righting reflex | 8 | 8 | 8 | 0.00684 |
| GO:0043501 | skeletal muscle adaptation | 16 | 16 | 16 | 0.00685 |
| GO:0090190 | positive regulation of branching involve... | 15 | 15 | 15 | 0.00686 |
| GO:2000821 | regulation of grooming behavior | 3 | 3 | 3 | 0.00687 |
| GO:0061013 | regulation of mRNA catabolic process | 18 | 18 | 18 | 0.00692 |
| GO:0021559 | trigeminal nerve development | 8 | 8 | 8 | 0.00694 |
| GO:0045822 | negative regulation of heart contraction | 19 | 19 | 19 | 0.00696 |
| GO:0098661 | inorganic anion transmembrane transport | 80 | 80 | 80 | 0.00699 |
| GO:2000681 | negative regulation of rubidium ion tran... | 3 | 3 | 3 | 0.00700 |
| GO:2000687 | negative regulation of rubidium ion tran... | 3 | 3 | 3 | 0.00700 |
| GO:0034698 | response to gonadotropin | 22 | 22 | 22 | 0.00702 |
| GO:0070572 | positive regulation of neuron projection... | 6 | 6 | 6 | 0.00706 |
| GO:0051195 | negative regulation of cofactor metaboli... | 9 | 9 | 9 | 0.00707 |
| GO:0051198 | negative regulation of coenzyme metaboli... | 9 | 9 | 9 | 0.00707 |
| GO:0097151 | positive regulation of inhibitory postsy... | 6 | 6 | 6 | 0.00709 |
| GO:0098828 | modulation of inihbitory postsynaptic po... | 6 | 6 | 6 | 0.00709 |
| GO:0006027 | glycosaminoglycan catabolic process | 42 | 42 | 42 | 0.00710 |
| GO:0007098 | centrosome cycle | 45 | 45 | 45 | 0.00710 |
| GO:0042440 | pigment metabolic process | 30 | 30 | 30 | 0.00712 |
| GO:0010960 | magnesium ion homeostasis | 9 | 9 | 9 | 0.00713 |
| GO:0006301 | postreplication repair | 31 | 31 | 31 | 0.00722 |
| GO:0070633 | transepithelial transport | 6 | 6 | 6 | 0.00723 |
| GO:0072205 | metanephric collecting duct development | 6 | 6 | 6 | 0.00724 |
| GO:0001514 | selenocysteine incorporation | 6 | 6 | 6 | 0.00725 |
| GO:0006451 | translational readthrough | 6 | 6 | 6 | 0.00725 |
| GO:0071286 | cellular response to magnesium ion | 4 | 4 | 4 | 0.00725 |
| GO:1900037 | regulation of cellular response to hypox... | 7 | 7 | 7 | 0.00726 |
| GO:0010839 | negative regulation of keratinocyte prol... | 12 | 12 | 12 | 0.00727 |
| GO:0045342 | MHC class II biosynthetic process | 11 | 11 | 11 | 0.00728 |
| GO:0090189 | regulation of branching involved in uret... | 18 | 18 | 18 | 0.00729 |
| GO:0030011 | maintenance of cell polarity | 9 | 9 | 9 | 0.00729 |
| GO:0044786 | cell cycle DNA replication | 24 | 24 | 24 | 0.00730 |
| GO:0072525 | pyridine-containing compound biosyntheti... | 9 | 9 | 9 | 0.00731 |
| GO:0070307 | lens fiber cell development | 11 | 11 | 11 | 0.00731 |
| GO:0043631 | RNA polyadenylation | 24 | 24 | 24 | 0.00731 |
| GO:0046189 | phenol-containing compound biosynthetic ... | 21 | 21 | 21 | 0.00734 |
| GO:0022010 | central nervous system myelination | 12 | 12 | 12 | 0.00735 |
| GO:0032291 | axon ensheathment in central nervous sys... | 12 | 12 | 12 | 0.00735 |
| GO:0060670 | branching involved in labyrinthine layer... | 9 | 9 | 9 | 0.00735 |
| GO:0035931 | mineralocorticoid secretion | 5 | 5 | 5 | 0.00736 |
| GO:0035932 | aldosterone secretion | 5 | 5 | 5 | 0.00736 |
| GO:1902043 | positive regulation of extrinsic apoptot... | 11 | 11 | 11 | 0.00738 |
| GO:0032461 | positive regulation of protein oligomeri... | 16 | 16 | 16 | 0.00740 |
| GO:0033572 | transferrin transport | 24 | 24 | 24 | 0.00741 |
| GO:0051985 | negative regulation of chromosome segreg... | 29 | 29 | 29 | 0.00741 |
| GO:0043353 | enucleate erythrocyte differentiation | 8 | 8 | 8 | 0.00743 |
| GO:0015012 | heparan sulfate proteoglycan biosyntheti... | 22 | 22 | 22 | 0.00749 |
| GO:0001964 | startle response | 18 | 18 | 18 | 0.00750 |
| GO:0030952 | establishment or maintenance of cytoskel... | 6 | 6 | 6 | 0.00750 |
| GO:0071474 | cellular hyperosmotic response | 7 | 7 | 7 | 0.00752 |
| GO:1901077 | regulation of relaxation of muscle | 8 | 8 | 8 | 0.00755 |
| GO:0030002 | cellular anion homeostasis | 11 | 11 | 11 | 0.00755 |
| GO:0030320 | cellular monovalent inorganic anion home... | 11 | 11 | 11 | 0.00755 |
| GO:0031069 | hair follicle morphogenesis | 24 | 24 | 24 | 0.00757 |
| GO:0003073 | regulation of systemic arterial blood pr... | 51 | 51 | 51 | 0.00758 |
| GO:1903793 | positive regulation of anion transport | 32 | 32 | 32 | 0.00762 |
| GO:0060148 | positive regulation of posttranscription... | 5 | 5 | 5 | 0.00762 |
| GO:0044381 | glucose import in response to insulin st... | 12 | 12 | 12 | 0.00763 |
| GO:2001273 | regulation of glucose import in response... | 12 | 12 | 12 | 0.00763 |
| GO:0033146 | regulation of intracellular estrogen rec... | 20 | 20 | 20 | 0.00763 |
| GO:1902259 | regulation of delayed rectifier potassiu... | 12 | 12 | 12 | 0.00764 |
| GO:0003032 | detection of oxygen | 3 | 3 | 3 | 0.00772 |
| GO:0016572 | histone phosphorylation | 21 | 21 | 21 | 0.00774 |
| GO:0033145 | positive regulation of intracellular ste... | 10 | 10 | 10 | 0.00775 |
| GO:0090219 | negative regulation of lipid kinase acti... | 4 | 4 | 4 | 0.00775 |
| GO:0071425 | hematopoietic stem cell proliferation | 16 | 16 | 16 | 0.00779 |
| GO:2000107 | negative regulation of leukocyte apoptot... | 25 | 25 | 25 | 0.00788 |
| GO:0061303 | cornea development in camera-type eye | 6 | 6 | 6 | 0.00790 |
| GO:0071636 | positive regulation of transforming grow... | 11 | 11 | 11 | 0.00793 |
| GO:0032369 | negative regulation of lipid transport | 13 | 13 | 13 | 0.00796 |
| GO:1902991 | regulation of amyloid precursor protein ... | 9 | 9 | 9 | 0.00799 |
| GO:0045738 | negative regulation of DNA repair | 9 | 9 | 9 | 0.00799 |
| GO:0003188 | heart valve formation | 9 | 9 | 9 | 0.00799 |
| GO:0035767 | endothelial cell chemotaxis | 17 | 17 | 17 | 0.00800 |
| GO:0048671 | negative regulation of collateral sprout... | 7 | 7 | 7 | 0.00802 |
| GO:0045541 | negative regulation of cholesterol biosy... | 3 | 3 | 3 | 0.00803 |
| GO:0090206 | negative regulation of cholesterol metab... | 3 | 3 | 3 | 0.00803 |
| GO:2000242 | negative regulation of reproductive proc... | 16 | 16 | 16 | 0.00805 |
| GO:0043983 | histone H4-K12 acetylation | 7 | 7 | 7 | 0.00806 |
| GO:0033240 | positive regulation of cellular amine me... | 7 | 7 | 7 | 0.00808 |
| GO:0051014 | actin filament severing | 6 | 6 | 6 | 0.00808 |
| GO:0072319 | vesicle uncoating | 3 | 3 | 3 | 0.00809 |
| GO:0014854 | response to inactivity | 7 | 7 | 7 | 0.00812 |
| GO:0086015 | SA node cell action potential | 7 | 7 | 7 | 0.00812 |
| GO:0086018 | SA node cell to atrial cardiac muscle ce... | 7 | 7 | 7 | 0.00812 |
| GO:0086070 | SA node cell to atrial cardiac muscle ce... | 7 | 7 | 7 | 0.00812 |
| GO:1901522 | positive regulation of transcription fro... | 24 | 24 | 24 | 0.00812 |
| GO:0035584 | calcium-mediated signaling using intrace... | 13 | 13 | 13 | 0.00812 |
| GO:0006927 | transformed cell apoptotic process | 6 | 6 | 6 | 0.00812 |
| GO:0055022 | negative regulation of cardiac muscle ti... | 10 | 10 | 10 | 0.00816 |
| GO:0061117 | negative regulation of heart growth | 10 | 10 | 10 | 0.00816 |
| GO:0003171 | atrioventricular valve development | 14 | 14 | 14 | 0.00816 |
| GO:0000291 | nuclear-transcribed mRNA catabolic proce... | 17 | 17 | 17 | 0.00817 |
| GO:0036092 | phosphatidylinositol-3-phosphate biosynt... | 12 | 12 | 12 | 0.00818 |
| GO:1900449 | regulation of glutamate receptor signali... | 25 | 25 | 25 | 0.00819 |
| GO:0050764 | regulation of phagocytosis | 38 | 38 | 38 | 0.00820 |
| GO:0016048 | detection of temperature stimulus | 13 | 13 | 13 | 0.00823 |
| GO:0010591 | regulation of lamellipodium assembly | 23 | 23 | 23 | 0.00825 |
| GO:0014856 | skeletal muscle cell proliferation | 9 | 9 | 9 | 0.00826 |
| GO:1901998 | toxin transport | 28 | 28 | 28 | 0.00827 |
| GO:0060600 | dichotomous subdivision of an epithelial... | 9 | 9 | 9 | 0.00827 |
| GO:0031532 | actin cytoskeleton reorganization | 61 | 61 | 61 | 0.00829 |
| GO:0043278 | response to morphine | 19 | 19 | 19 | 0.00833 |
| GO:2000272 | negative regulation of receptor activity | 11 | 11 | 11 | 0.00834 |
| GO:2000171 | negative regulation of dendrite developm... | 21 | 21 | 21 | 0.00835 |
| GO:0048388 | endosomal lumen acidification | 3 | 3 | 3 | 0.00839 |
| GO:0006998 | nuclear envelope organization | 55 | 55 | 55 | 0.00842 |
| GO:0023021 | termination of signal transduction | 3 | 3 | 3 | 0.00847 |
| GO:0050923 | regulation of negative chemotaxis | 4 | 4 | 4 | 0.00852 |
| GO:0071503 | response to heparin | 4 | 4 | 4 | 0.00852 |
| GO:0071504 | cellular response to heparin | 4 | 4 | 4 | 0.00852 |
| GO:0061179 | negative regulation of insulin secretion... | 7 | 7 | 7 | 0.00854 |
| GO:0003241 | growth involved in heart morphogenesis | 3 | 3 | 3 | 0.00855 |
| GO:0031050 | dsRNA fragmentation | 19 | 19 | 19 | 0.00855 |
| GO:0070918 | production of small RNA involved in gene... | 19 | 19 | 19 | 0.00855 |
| GO:0003177 | pulmonary valve development | 6 | 6 | 6 | 0.00856 |
| GO:0003184 | pulmonary valve morphogenesis | 6 | 6 | 6 | 0.00856 |
| GO:0003190 | atrioventricular valve formation | 6 | 6 | 6 | 0.00856 |
| GO:0060842 | arterial endothelial cell differentiatio... | 6 | 6 | 6 | 0.00856 |
| GO:0035909 | aorta morphogenesis | 18 | 18 | 18 | 0.00857 |
| GO:0060390 | regulation of SMAD protein import into n... | 12 | 12 | 12 | 0.00859 |
| GO:0035058 | nonmotile primary cilium assembly | 12 | 12 | 12 | 0.00860 |
| GO:0035234 | ectopic germ cell programmed cell death | 3 | 3 | 3 | 0.00866 |
| GO:1902263 | apoptotic process involved in embryonic ... | 3 | 3 | 3 | 0.00866 |
| GO:0007586 | digestion | 60 | 60 | 60 | 0.00869 |
| GO:0003263 | cardioblast proliferation | 8 | 8 | 8 | 0.00869 |
| GO:0003264 | regulation of cardioblast proliferation | 8 | 8 | 8 | 0.00869 |
| GO:0003266 | regulation of secondary heart field card... | 8 | 8 | 8 | 0.00869 |
| GO:0007097 | nuclear migration | 9 | 9 | 9 | 0.00872 |
| GO:0010712 | regulation of collagen metabolic process | 20 | 20 | 20 | 0.00880 |
| GO:0000038 | very long-chain fatty acid metabolic pro... | 17 | 17 | 17 | 0.00881 |
| GO:0030225 | macrophage differentiation | 27 | 27 | 27 | 0.00885 |
| GO:0050961 | detection of temperature stimulus involv... | 9 | 9 | 9 | 0.00888 |
| GO:0050965 | detection of temperature stimulus involv... | 9 | 9 | 9 | 0.00888 |
| GO:0002507 | tolerance induction | 14 | 14 | 14 | 0.00889 |
| GO:0006972 | hyperosmotic response | 13 | 13 | 13 | 0.00889 |
| GO:0006089 | lactate metabolic process | 8 | 8 | 8 | 0.00890 |
| GO:0042503 | tyrosine phosphorylation of Stat3 protei... | 25 | 25 | 25 | 0.00891 |
| GO:0042516 | regulation of tyrosine phosphorylation o... | 25 | 25 | 25 | 0.00891 |
| GO:0042992 | negative regulation of transcription fac... | 30 | 30 | 30 | 0.00893 |
| GO:0045163 | clustering of voltage-gated potassium ch... | 3 | 3 | 3 | 0.00896 |
| GO:0015807 | L-amino acid transport | 46 | 46 | 46 | 0.00897 |
| GO:0060029 | convergent extension involved in organog... | 4 | 4 | 4 | 0.00897 |
| GO:1903020 | positive regulation of glycoprotein meta... | 17 | 17 | 17 | 0.00897 |
| GO:0035268 | protein mannosylation | 12 | 12 | 12 | 0.00900 |
| GO:0090335 | regulation of brown fat cell differentia... | 6 | 6 | 6 | 0.00902 |
| GO:1900744 | regulation of p38MAPK cascade | 16 | 16 | 16 | 0.00903 |
| GO:0072600 | establishment of protein localization to... | 12 | 12 | 12 | 0.00903 |
| GO:0035710 | CD4-positive  alpha-beta T cell activati... | 39 | 39 | 39 | 0.00904 |
| GO:1900748 | positive regulation of vascular endothel... | 3 | 3 | 3 | 0.00906 |
| GO:0009251 | glucan catabolic process | 22 | 22 | 22 | 0.00907 |
| GO:0061450 | trophoblast cell migration | 3 | 3 | 3 | 0.00907 |
| GO:1901163 | regulation of trophoblast cell migration | 3 | 3 | 3 | 0.00907 |
| GO:0006809 | nitric oxide biosynthetic process | 45 | 45 | 45 | 0.00907 |
| GO:0047496 | vesicle transport along microtubule | 18 | 18 | 18 | 0.00908 |
| GO:0007216 | G-protein coupled glutamate receptor sig... | 10 | 10 | 10 | 0.00911 |
| GO:0018200 | peptidyl-glutamic acid modification | 16 | 16 | 16 | 0.00915 |
| GO:0048743 | positive regulation of skeletal muscle f... | 3 | 3 | 3 | 0.00918 |
| GO:0035162 | embryonic hemopoiesis | 21 | 21 | 21 | 0.00919 |
| GO:0033046 | negative regulation of sister chromatid ... | 27 | 27 | 27 | 0.00921 |
| GO:0033048 | negative regulation of mitotic sister ch... | 27 | 27 | 27 | 0.00921 |
| GO:0045841 | negative regulation of mitotic metaphase... | 27 | 27 | 27 | 0.00921 |
| GO:0071174 | mitotic spindle checkpoint | 27 | 27 | 27 | 0.00921 |
| GO:1902100 | negative regulation of metaphase/anaphas... | 27 | 27 | 27 | 0.00921 |
| GO:2000816 | negative regulation of mitotic sister ch... | 27 | 27 | 27 | 0.00921 |
| GO:0014051 | gamma-aminobutyric acid secretion | 7 | 7 | 7 | 0.00922 |
| GO:0043586 | tongue development | 16 | 16 | 16 | 0.00922 |
| GO:0001547 | antral ovarian follicle growth | 5 | 5 | 5 | 0.00923 |
| GO:0001893 | maternal placenta development | 21 | 21 | 21 | 0.00925 |
| GO:1902165 | regulation of intrinsic apoptotic signal... | 9 | 9 | 9 | 0.00931 |
| GO:0031577 | spindle checkpoint | 32 | 32 | 32 | 0.00931 |
| GO:0006521 | regulation of cellular amino acid metabo... | 38 | 38 | 38 | 0.00933 |
| GO:0072300 | positive regulation of metanephric glome... | 4 | 4 | 4 | 0.00933 |
| GO:0032675 | regulation of interleukin-6 production | 57 | 57 | 57 | 0.00935 |
| GO:0051580 | regulation of neurotransmitter uptake | 13 | 13 | 13 | 0.00936 |
| GO:0071347 | cellular response to interleukin-1 | 56 | 56 | 56 | 0.00936 |
| GO:0002029 | desensitization of G-protein coupled rec... | 11 | 11 | 11 | 0.00937 |
| GO:0022401 | negative adaptation of signaling pathway | 11 | 11 | 11 | 0.00937 |
| GO:0048791 | calcium ion-dependent exocytosis of neur... | 22 | 22 | 22 | 0.00938 |
| GO:0048570 | notochord morphogenesis | 8 | 8 | 8 | 0.00940 |
| GO:0043931 | ossification involved in bone maturation | 14 | 14 | 14 | 0.00945 |
| GO:0042445 | hormone metabolic process | 109 | 109 | 109 | 0.00948 |
| GO:0061213 | positive regulation of mesonephros devel... | 18 | 18 | 18 | 0.00949 |
| GO:0002381 | immunoglobulin production involved in im... | 25 | 25 | 25 | 0.00949 |
| GO:0003406 | retinal pigment epithelium development | 6 | 6 | 6 | 0.00952 |
| GO:1903726 | negative regulation of phospholipid meta... | 6 | 6 | 6 | 0.00956 |
| GO:0045932 | negative regulation of muscle contractio... | 17 | 17 | 17 | 0.00957 |
| GO:0060397 | JAK-STAT cascade involved in growth horm... | 15 | 15 | 15 | 0.00962 |
| GO:2000323 | negative regulation of glucocorticoid re... | 6 | 6 | 6 | 0.00962 |
| GO:0006360 | transcription from RNA polymerase I prom... | 35 | 35 | 35 | 0.00963 |
| GO:0003254 | regulation of membrane depolarization | 28 | 28 | 28 | 0.00964 |
| GO:0060449 | bud elongation involved in lung branchin... | 5 | 5 | 5 | 0.00964 |
| GO:0070206 | protein trimerization | 19 | 19 | 19 | 0.00965 |
| GO:0030903 | notochord development | 14 | 14 | 14 | 0.00967 |
| GO:0006270 | DNA replication initiation | 21 | 21 | 21 | 0.00968 |
| GO:1904251 | regulation of bile acid metabolic proces... | 5 | 5 | 5 | 0.00971 |
| GO:0007412 | axon target recognition | 3 | 3 | 3 | 0.00973 |
| GO:0007625 | grooming behavior | 11 | 11 | 11 | 0.00974 |
| GO:0021892 | cerebral cortex GABAergic interneuron di... | 11 | 11 | 11 | 0.00974 |
| GO:0009886 | post-embryonic morphogenesis | 12 | 12 | 12 | 0.00977 |
| GO:0038107 | nodal signaling pathway involved in dete... | 3 | 3 | 3 | 0.00977 |
| GO:1900094 | regulation of transcription from RNA pol... | 3 | 3 | 3 | 0.00977 |
| GO:1900164 | nodal signaling pathway involved in dete... | 3 | 3 | 3 | 0.00977 |
| GO:0046636 | negative regulation of alpha-beta T cell... | 11 | 11 | 11 | 0.00981 |
| GO:0050931 | pigment cell differentiation | 22 | 22 | 22 | 0.00984 |
| GO:0072520 | seminiferous tubule development | 5 | 5 | 5 | 0.00984 |
| GO:0001886 | endothelial cell morphogenesis | 11 | 11 | 11 | 0.00986 |
| GO:1901621 | negative regulation of smoothened signal... | 5 | 5 | 5 | 0.00988 |
| GO:0046164 | alcohol catabolic process | 34 | 34 | 34 | 0.00988 |
| GO:0032464 | positive regulation of protein homooligo... | 6 | 6 | 6 | 0.00992 |
| GO:0055012 | ventricular cardiac muscle cell differen... | 16 | 16 | 16 | 0.00992 |
| GO:0015874 | norepinephrine transport | 12 | 12 | 12 | 0.00993 |
| GO:2000846 | regulation of corticosteroid hormone sec... | 8 | 8 | 8 | 0.00993 |
| GO:0046666 | retinal cell programmed cell death | 4 | 4 | 4 | 0.00994 |
| GO:0045909 | positive regulation of vasodilation | 16 | 16 | 16 | 0.00994 |
| GO:0050687 | negative regulation of defense response ... | 11 | 11 | 11 | 0.00995 |
| GO:0032229 | negative regulation of synaptic transmis... | 5 | 5 | 5 | 0.00999 |
| GO:0061178 | regulation of insulin secretion involved... | 36 | 36 | 36 | 0.00999 |

**Supplementary Figures Legends**:

**Supplementary Figure 1** - DNA methylation changes in microRNAs´ promoters in prostate cancer (PCa), determined by Infinium HumanMethylation450 BeadChip in 25 PCa tissues and 5 morphologically normal prostate tissue (MNPT) samples. (A) Schematic representation of the DNA methylation mapping approach used to identify new aberrantly methylated miRNAs. (B) β-Values from representative microRNAs. (C) Genomic location of the differentially methylated microRNAs. (D) DNA methylation levels determined by pyrosequencing in PCa cell lines, showing that all cell lines tested display aberrant DNA methylation in the promoter of miR-130b~301b cluster. (*p<0.05, **p<0.01, ***p<0.001).

**Supplementary Figure 2** – Confirmation of miR-130b and miR-301b expression levels by RT-qPCR. (A-C) miR-130b or miR-301b expression levels after transfection with anti-miR-NC, anti-miR-130b and, anti-miR-301b in LNCaP, DU145 and, PC3, respectively. (D) Overexpression of miR-130b and miR-310b in PC3 cells. The analyses were conducted 72h post transfections. All data are presented as mean of three independent experiments ± s.d. (*p<0.05, **p<0.01, ***p<0.001).

**Supplementary Figure 3 –** Cross-validation of deregulated genes upon cluster miR-130b-301b manipulation in the TCGA cohort. Boxplot depiction of the cancer versus normal differentially expressed mRNAs among the TCGA prostate RNA-seq cohort. Green and red squares refer to down-regulated and overexpressed genes in PCa versus NAT samples, respectively. Each point represents one RNA-seq tissue sample.

**Supplementary Figure 4** – RT-qPCR expression changes in multiple genes involved in invasion and epithelial to mesenchymal transition (EMT), suggesting functional specialization among members of miR-130b-301b polycistron. Gene expression patterns by (A) induction of pre-miR-130b or pre-miR-301b or (B) after endogenous levels blocking. The analyses were conducted 72h post transfections. All data is presented as mean of three independent experiments ± s.d. (*p<0.05, **p<0.01, ***p<0.001).

**Supplementary Figure 5** – Morphological alterations in PC3 cells after miR-130b or miR-301b overexpression. Transfection of (A) pre-miR-NC, (B) pre-miR-130b, or (C) pre-miR-301b. The restoration of miR-130b or miR-301b expression induced cell polarization and epithelial-like phenotype, suggesting a mesenchymal to epithelial transition.

**Supplementary Figure 6** – Morphological alterations in PC3 cells upon miR-130b or miR-301b knockdown. (A) PC3 cells transfected with anti-miR-NC, (B) anti-miR-130b or (C) anti-miR-301b. Inhibition of endogenous miR-130b or miR-301b caused PC3 cells to acquire a more pronounced fibroblast-like morphology, compatible with a mesenchymal-type phenotype.

**Supplementary Figure 7 –** *LMNB1* 3’UTR putative binding sites for miR-130b and miR-301b.
