# Supplementary material for: Downregulation of miR-130b~301b cluster is mediated by aberrant promoter methylation and impairs cellular senescence in prostate cancer
Source: J Hematol Oncol. 2017 Feb 6;10:43. doi: 10.1186/s13045-017-0415-1 (PMC5294724; doi:10.1186/s13045-017-0415-1)
Supplement: Additional file 2: Figure S1. — DNA methylation changes in microRNAs´ promoters in prostate cancer (PCa), determined by Infinium HumanMethylation450 BeadChip in 25 PCa tissues and 5 morphologically normal prostate tissue (MNPT) samples. (A) Schematic representation of the DNA methylation mapping approach used to identify new aberrantly methylated miRNAs. (B) β values from representative microRNAs. (C) Genomic location of the differentially methylated microRNAs. (D) DNA methylation levels determined by pyrosequencing in PCa cell lines, showing that all cell lines tested display aberrant DNA methylation in the promoter of miR-130b~301b cluster. (*p < 0.05, **p < 0.01, ***p < 0.001). Figure S2. Confirmation of miR-130b and miR-301b expression levels by RT-qPCR. (A–C) miR-130b or miR-301b expression levels after transfection with anti-miR-NC, anti-miR-130b and, anti-miR-301b in LNCaP, DU145 and, PC3, respectively. (D) Overexpression of miR-130b and miR-310b in PC3 cells. The analyses were conducted 72 h post-transfections. All data are presented as mean of three independent experiments ± s.d. (*p < 0.05, **p < 0.01, ***p < 0.001). Figure S3. Cross-validation of deregulated genes upon cluster miR-130b-301b manipulation in the TCGA cohort. Boxplot depiction of the cancer versus normal differentially expressed mRNAs among the TCGA prostate RNA-seq cohort. Green and red squares refer to down-regulated and overexpressed genes in PCa versus NAT samples, respectively. Each point represents one RNA-seq tissue sample. Figure S4. RT-qPCR expression changes in multiple genes involved in invasion and epithelial to mesenchymal transition (EMT), suggesting functional specialization among members of miR-130b-301b polycistron. Gene expression patterns by (A) induction of pre-miR-130b or pre-miR-301b or (B) after endogenous levels blocking. The analyses were conducted 72 h post-transfections. All data is presented as mean of three independent experiments ± s.d. (*p < 0.05, **p < 0.01, ***p < 0.001). Figure S5. Morphol [file 13045_2017_415_MOESM2_ESM.pdf]

Supplementary Figure 1

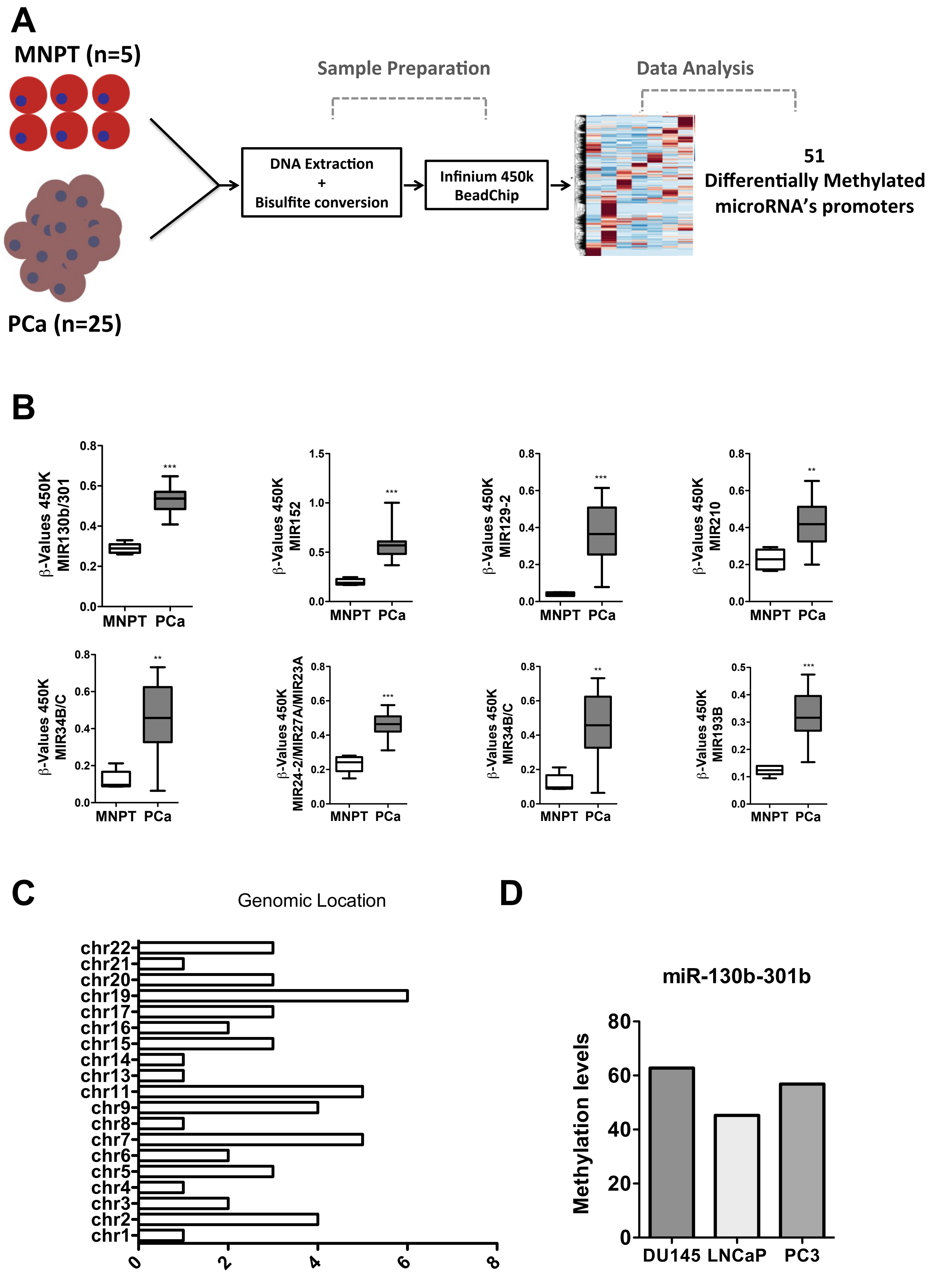

Supplementary Figure 2

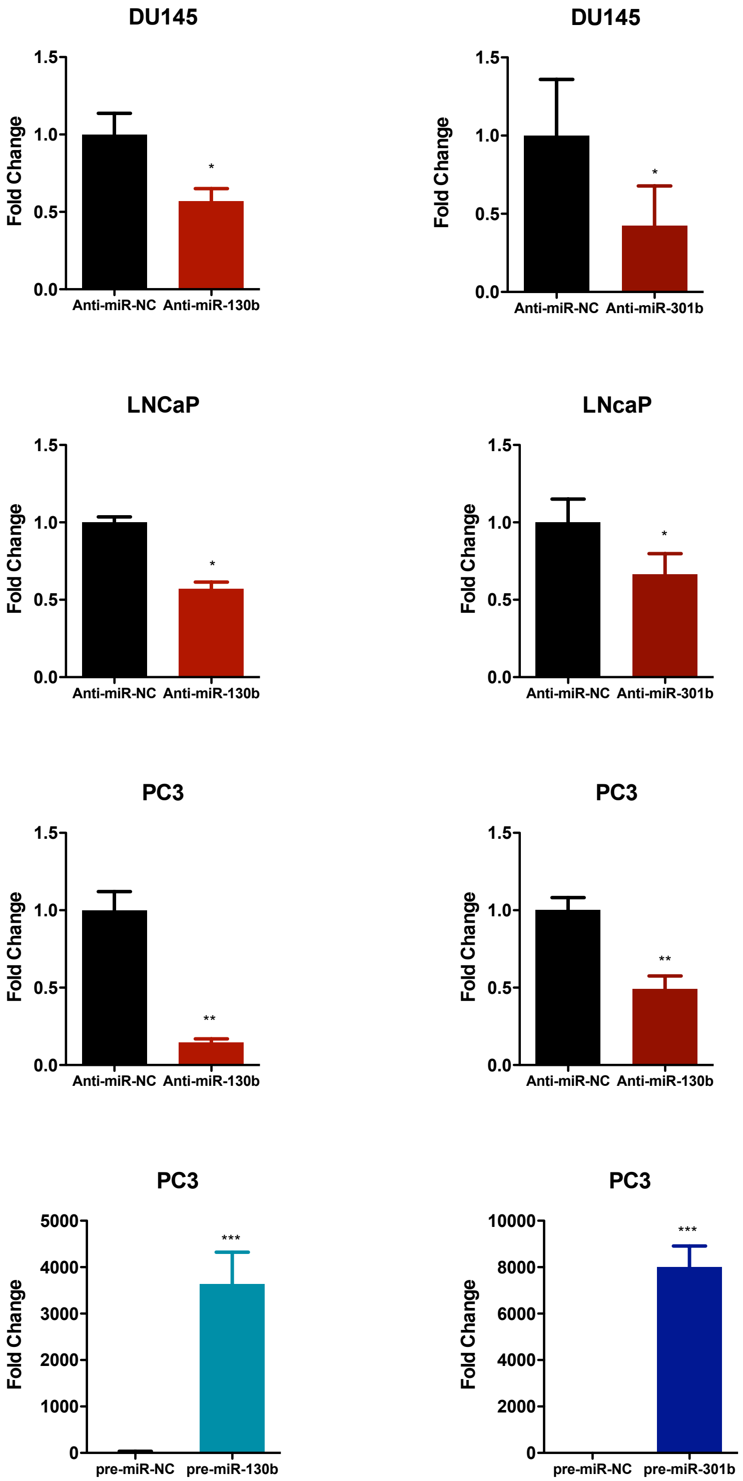

Supplementary Figure 3

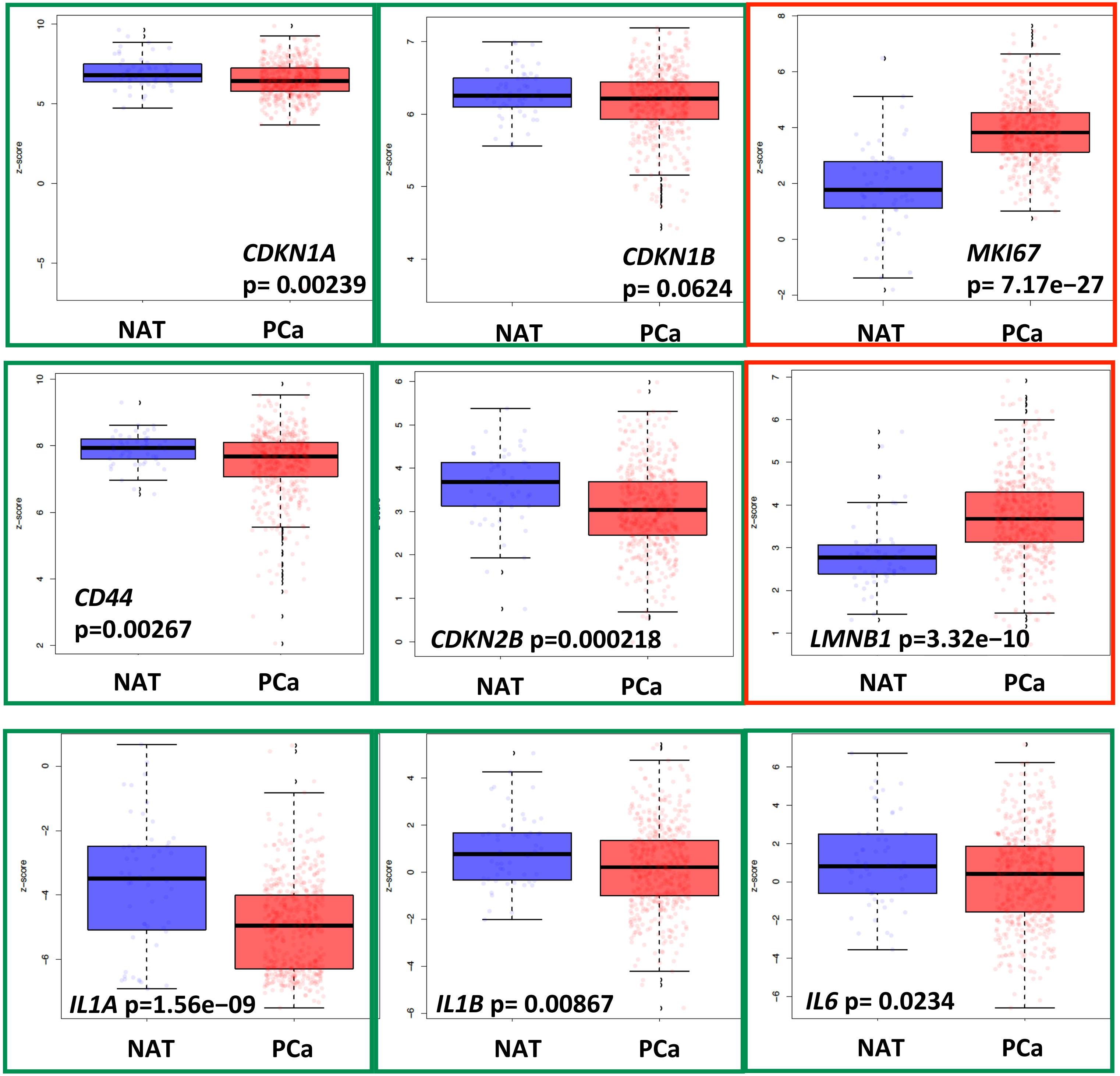

Supplementary Figure 4

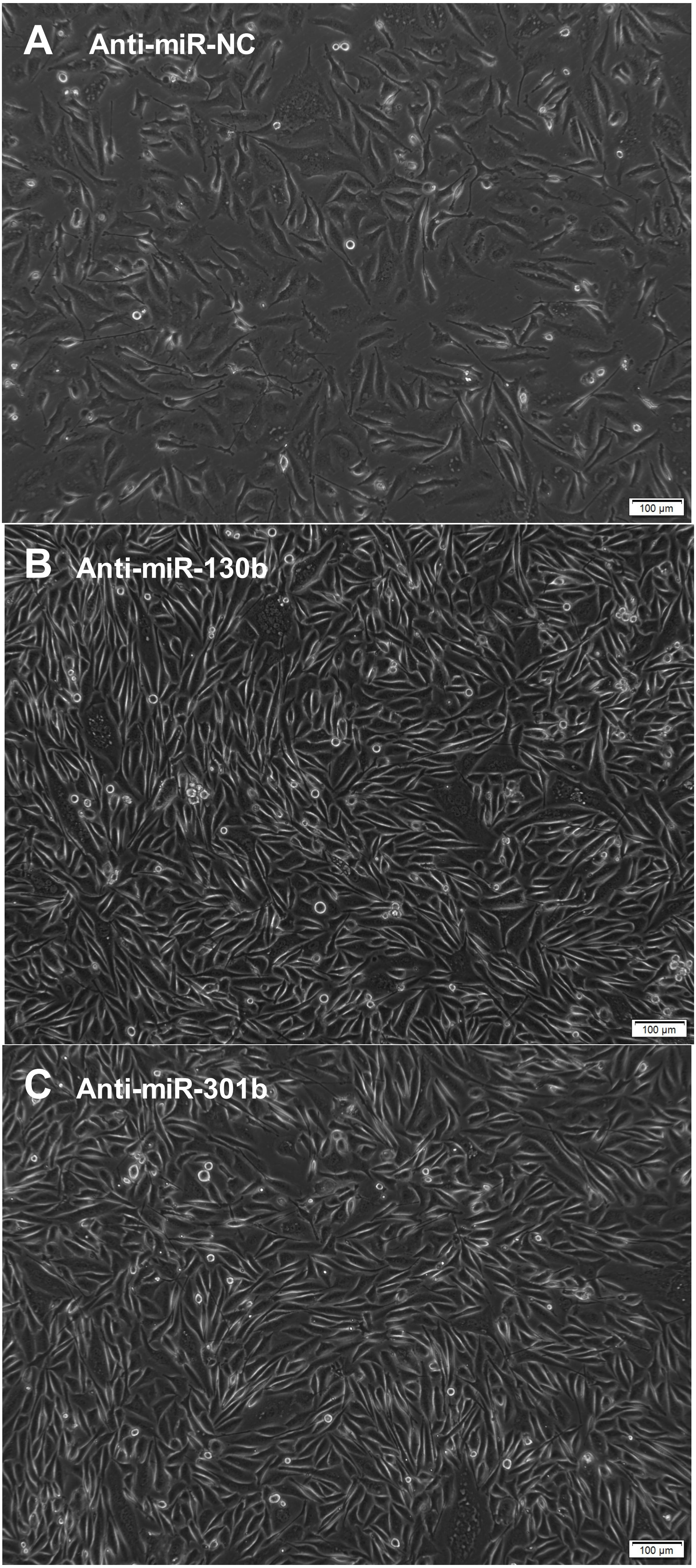

Supplementary Figure 5

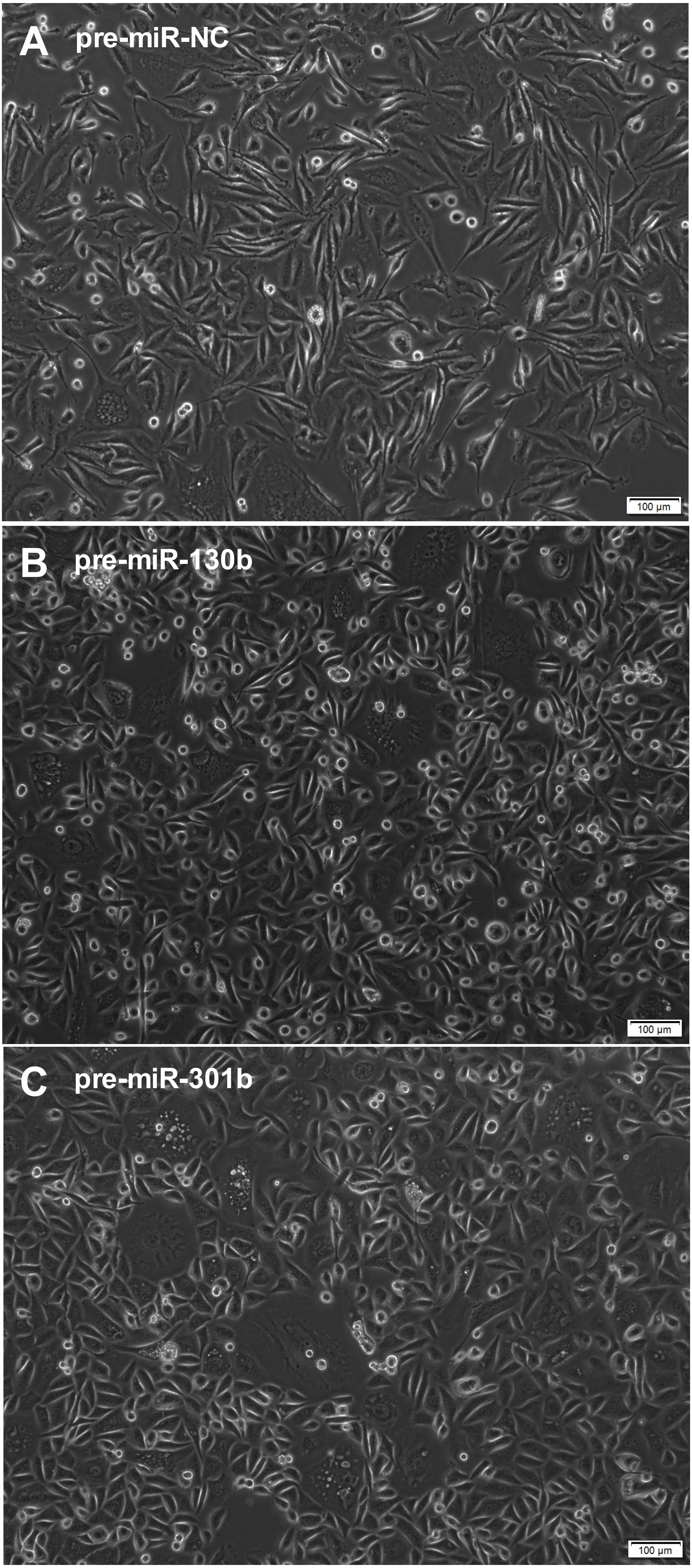

Supplementary Figure 6

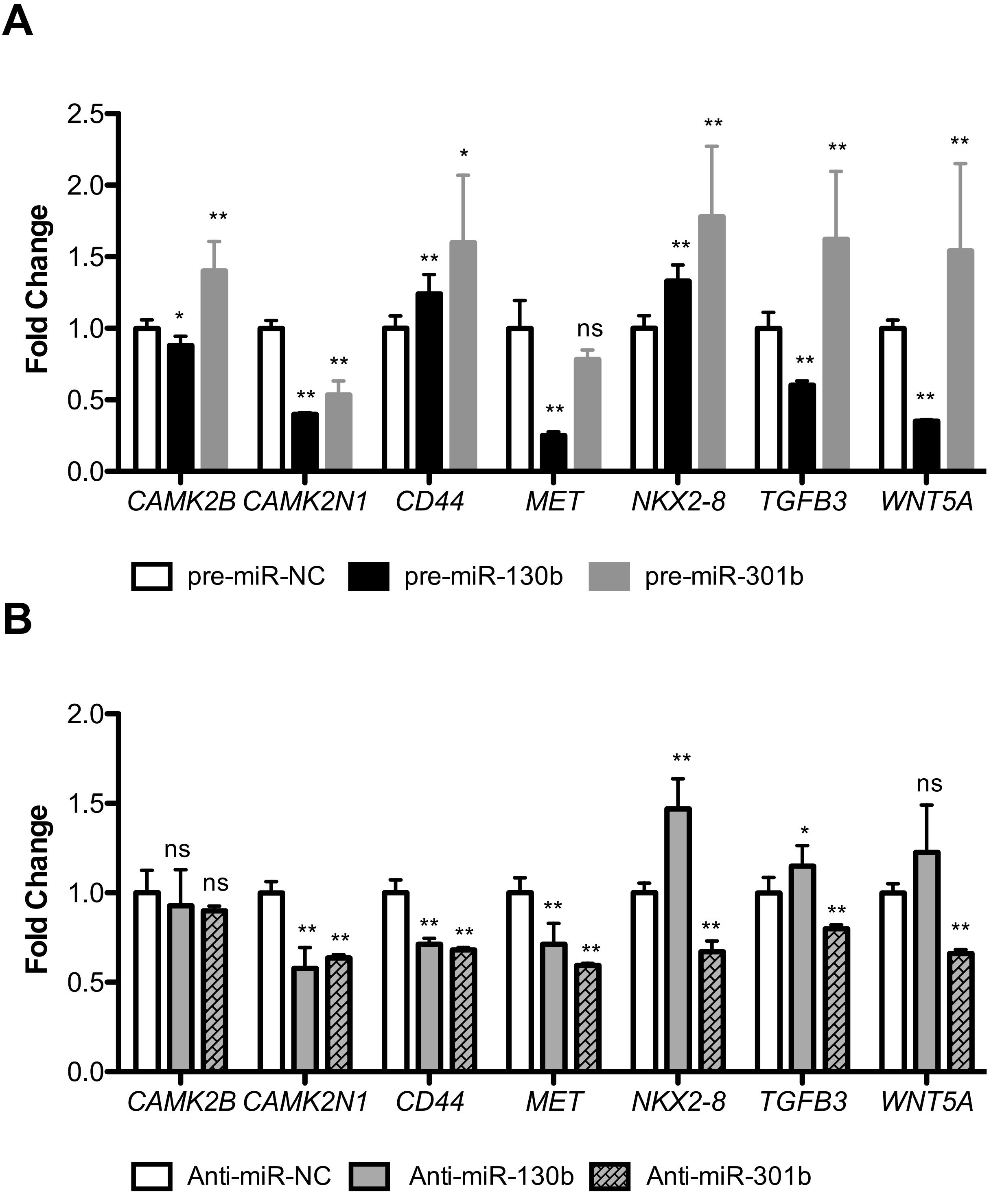

Supplementary Figure 7

```
      3' uacgggAAAGUAGUAAC-GUGAc 5' hsa-miR-130b
             ||||  |:   ||| ||||
132:5' ccuuaaUUUCCUUUUUGACACUg 3' LMNB1

      3' uacgggaaagUAGUAAC-GUGAc 5' hsa-miR-130b
                 ||:||||  ||||
194:5' caagaugugaAUUAUUGACACUg 3' LMNB1

      3’ cgaaacuguuauAGUAAC-GUGAc 5’ hsa-miR-301b
                  |:   |||  ||||
132:5' ccuuaaUUUCCUUUUUGACACUg 3' LMNB1

      3’ cgaaacuguuaUAGUAAC-GUGAc 5’ hsa-miR-301b
                 ||:||||  ||||
194:5' caagaugugaAUUAUUGACACUg 3' LMNB1
```
